# Multivalency Beats Complexity: A Study on the Cell Uptake of Carbohydrate Functionalized Nanocarriers to Dendritic Cells

**DOI:** 10.3390/cells9092087

**Published:** 2020-09-12

**Authors:** Matthias Krumb, Marie-Luise Frey, Jens Langhanki, Robert Forster, Danuta Kowalczyk, Volker Mailänder, Katharina Landfester, Till Opatz

**Affiliations:** 1 Department of Chemistry, Johannes Gutenberg-University Mainz, Duesbergweg 10–14, 55128 Mainz, Germany; krumb@uni-mainz.de (M.K.); jens-gerald.langhanki@basf.com (J.L.); rforster@students.uni-mainz.de (R.F.); kowalda@uni-mainz.de (D.K.); 2 Max Planck Institute for Polymer Research (MPIP), Ackermannweg 10, 55128 Mainz, Germany; frey@mpip-mainz.mpg.de (M.-L.F.); volker.mailaender@unimedizin-mainz.de (V.M.); 3 Dermatology Clinic, University Medical Center of the Johannes Gutenberg-University Mainz, Langenbeckstr. 1, 55131 Mainz, Germany

**Keywords:** carbohydrates, glycodendrons, dendritic cells, nanocapsules, cell targeting

## Abstract

Herein, we report the synthesis of carbohydrate and glycodendron structures for dendritic cell targeting, which were subsequently bound to hydroxyethyl starch (HES) nanocapsules prepared by the inverse miniemulsion technique. The uptake of the carbohydrate-functionalized HES nanocapsules into immature human dendritic cells (hDCs) revealed a strong dependence on the used carbohydrate. A multivalent mannose-terminated dendron was found to be far superior in uptake compared to the structurally more complex oligosaccharides used.

## 1. Introduction

The synthesis and characterization of nanocarrier systems is an emerging discipline in modern nanomedicine, where bioavailability, biodegradability, adequate release, efficient coupling of the drug to the nanocarrier (e.g., by encapsulation) and also targeted delivery of the drug to its destination play key roles and need to be finely tuned [1]. In particular, targeted drug delivery to certain cell types is a highly desirable strategy and the targeting of dendritic cells (DCs) in terms of tumor immunotherapy has gained wide attention in recent years [2]. Due to their ability to activate the adaptive immune response, DCs play a key role in delivering information to the immune system. Therefore, they can serve as important mediators in nanomedicine to deliver antigens upon targeting and cell uptake of antigen-loaded nanocarriers [3]. The targeting of DCs is possible through their surface receptor dendritic cell-specific intercellular adhesion molecule-3-grabbing non-integrin (DC-SIGN), which is known to bind carbohydrate recognition structures through a C-terminal carbohydrate recognition domain (CRD) [4,5,6]. The DC-SIGN receptor is a tetramer that carries four of these CRD domains and can therefore theoretically bind up to four ligands simultaneously [7]. As a consequence, DC-SIGN specifically and strongly binds high-mannose-type oligosaccharides, in which mannose units are presented in a multivalent manner. It can therefore be deduced that multivalency might be a universal promising strategy to maximize the carbohydrate lectin interactions in terms of DC-SIGN targeting with nanocarriers.

In general, multivalency is a key strategy within nature to achieve strong but yet reversible interactions [8]. Nature uses multivalency in key biological processes such as cell–cell-communication or the recognition of pathogens, like viruses, on the cell surface to just name a few examples [9]. It appears particularly remarkable that nature has orchestrated such highly complex biological processes which are traced back to rather weak single physicochemical interactions. For example, the binding of a single carbohydrate to a lectin is often weak, while simultaneous binding of many, often branched and complex oligosaccharides to lectins creates strong signals [10]. The binding pocket of lectins is located on the surface of the protein, where the binding of carbohydrates results from the complexation of bound metal ions or amino acid residues. As a result, this binding does not occur deep inside the protein, which makes it difficult to achieve a strong binding in tight, deep pockets. [11,12]. However, since biological processes run in a highly orchestrated manner, the minor influence of single signals in comparison to the major influence of multivalent signals makes sense [13,14]. Another explanation is that cell surface receptors seldom operate alone but rather form clusters at the cell surface to permit signal transduction [14]. A multivalent ligand could therefore help to achieve such a clustering process at the cell surface. As previously mentioned, some cell surface receptors like DC-SIGN exhibit multiple CRDs, in which a multivalent ligand could occupy more than one pocket to amplify the binding signal [12]. Secondly, a statistical rebinding of a single ligand is increasingly probable if a multivalent ligand is present, which increases the effective concentration against the receptor [15]. Over the years, the synthesis of glycodendrimers and dendrons has greatly evolved and a variety of platforms were produced that serve as a basis for multivalent glyco-targeting [16,17,18]. These systems become more useful if they are applied for the surface functionalization of nanocarriers, which can be used to protect and safely transport therapeutics to specific sites of action, such as specific cell populations. In particular, biopolymers such as hydroxyethyl starch (HES) are particularly suitable candidates for transport because they are biocompatible, degradable and many of them are already medically approved [19]. 

The goal of this project was therefore to synthesize a series of carbohydrate-based ligands suitable for DC-SIGN targeting and to compare them to a multivalent ligand in form of a glycodendron. With this series in hand, we analyzed whether the carbohydrate lectin binding is enhanced by a multivalent ligand, thus enabling effective targeting. The targeting ability of these ligands to DCs was subsequently explored through conjugation to a carrier system, where HES nanocapsules were chosen as an ideal carrier platform for the latter since they are produced from medically approved material, but also shown to be robust and biocompatible in earlier studies [19,20,21]. Afterwards, the targeting ability of the carbohydrate HES conjugates was explored in cell uptake experiments and confirmed through follow up blocking studies with antibodies.

## 2. Materials and Methods

### 2.1. Solvents and Reagents

Unless stated otherwise, all chemicals were obtained from commercial suppliers such as Sigma Aldrich (St. Louis, MO, USA), Thermo Fisher Scientific (Waltham, MS, USA), TCI (Tokyo, Japan), ABCR (Karlsruhe, Germany) and used without purification. Deuterated solvents were purchased from Deutero GmbH (Kastellaun, Germany). Anhydrous acetonitrile and dichloromethane were distilled from calcium hydride under nitrogen atmosphere. Extra dry dimethylformamide (DMF) was purchased from Acros Organics (AcroSeal^®^ glass bottle, part of Thermo Fisher, Waltham, MS, USA). The eluents for column chromatography (cyclohexane (^c^Hex) and ethyl acetate (EtOAc)) were purchased in technical grade and distilled prior to use. Chloroform-d was stored over alumina (Brockmann activity I). Ampuwa^®^ water and hydroxyethyl starch (HES, M_w_: 200 kDa, 0.5, 3%), dissolved in an isotonic sodium chloride solution, was obtained from Fresenius Kabi (Bad Homburg, Germany) DBCO-PEG_4_-NHS ester was purchased from Jena Bioscience GmbH (Jena, Germany). The block copolymer poly((ethylene-*co*-butylene)-*b*-(ethylene oxide)) P(E/B)-*b*-EO) used as surfactant was synthesized according to previously published procedures from Schlaad et al. [22]. 

### 2.2. Reaction Conditions

All reactions involving air- or moisture-sensitive reagents or intermediates were performed under an inert atmosphere of argon in glassware that was oven dried using standard Schlenk techniques. Reaction temperatures referred to the temperature of the particular cooling/heating bath.

### 2.3. Chromatography

Chromatographic purification of products was performed using flash column chromatography of the indicated solvent system on silica gel (35–70 µm, Acros Organics unless otherwise noted. Alternatively, purification was performed on an Isolera^TM^ Flash Purification System, Biotage, Uppsala, Sweden) with an integrated diode array detector.

Thin-layer chromatography (TLC) was carried out on silica plates (TLC Silica 60 F254 by Merck, Darmstadt, Germany). UV active compounds were visualized using UV light (*λ* = 254 nm). Oxidation labile compounds were visualized by immersion in a solution of cerium(IV) sulfate (1.00 g) and phosphomolybdic acid (2.50 g) in water (95.0 mL) and concentrated sulfuric acid (4.00 mL) followed by heating. Alternatively, the TLC plates were immersed in a solution of *m*-methoxyphenol (0.10 mL) in ethanol (95.00 mL) and sulfuric acid (2.00 mL) followed by heating to stain saccharides specifically.

Analytical HPLC analysis was performed on an Agilent Technologies (Santa Clara, CA, USA) 1260 Infinity II system equipped with a binary pump and a diode array detector. An ACE (Aberdeen, UK) C18 PFP column (150 × 4.6 mm, 3 µm, 40 °C) with a flow rate of 1 mL/min was used.

Preparative reverse phase separation was carried out on a Smartline HPLC system (Knauer, Berlin, Germany) with mixtures of acetonitrile (HPLC grade) and water (HPLC grade) as eluents on an ACE 5 C18 PFP 150 × 30 mm column, at a flow rate of 37.5 mL/min. The eluents were degassed prior to use by means of ultrasonication for 30 min. Two Smartline K 1800 pumps (pump head size: 100 mL each, high pressure gradient; Knauer) and an S 2600 diode array detector (Knauer) were used. 

### 2.4. NMR Spectra

NMR spectra were recorded on a Bruker (Billerica, MA, USA) Avance-III HD (^1^H-NMR: 300 MHz, ^13^C-NMR: 75.5 MHz), a Bruker Avance-II (^1^H-NMR: 400 MHz, ^13^C-NMR: 100.6 MHz) or a Bruker Avance-III (^1^H-NMR: 600 MHz, ^13^C-NMR: 151.1 MHz) spectrometer. Chemical shifts are referenced to residual solvent signals (CDCl_3_: 7.26 ppm and 77.16 ppm, DMSO-d_6_: 2.50 ppm and 39.52 ppm for ^1^H-NMR and ^13^C-NMR respectively) and reported in parts per million (ppm) relative to tetramethylsilane (TMS). Multiplicities of NMR signals are abbreviated as follows: br = broad, s = singlet, d = doublet, t = triplet, q = quartet, m = multiplet and combinations thereof, app = apparent. The spectrometer was calibrated using α,α,α-trifluorotoluene in CDCl_3_ (−63.9 ppm).

### 2.5. Infrared Spectra

Infrared (IR-) spectra were recorded on a FTIR-spectrometer (Bruker, Billerica, MA, USA; Tensor 27) equipped with a diamond ATR unit and are reported in terms of frequency of absorption (cm^−1^).

### 2.6. UV-Vis Spectra

UV-Vis spectra were recorded on an Evolution 201 UV-Visible Spectrophotometer from Thermo Scientific.

### 2.7. Mass Spectra

Electron spray ionization (ESI) mass spectra were recorded on a 1200-series HPLC-system or a 1260-series Infinity II HPLC-system (Agilent-Technologies) with binary pump and integrated diode array detector coupled to a LC/MSD-Trap-XTC-mass spectrometer (Agilent-Technologies) or a LC/MSD Infinitylab LC/MSD (G6125B LC/MSD). High-resolution mass spectra were recorded on a Micromass-Q-TOF-Ultima-3-mass spectrometer (Waters, Milford, MA, USA) with LockSpray-interface and a suitable external calibrant. The reported high-resolution masses refer to the neutral molecules since the mass of the single electron (which is removed to form the cationic species) was considered during calibration. LIFDI mass spectra were recorded on a double focusing LIFDI sector field mass spectrometer (Thermo Fischer DFS).

### 2.8. Melting Points

Melting points were determined in open capillary tubes using a Krüss-Optronic (Hamburg, Germany) KSP 1 N thermoelectric melting point meter.

### 2.9. Optical Rotations

Optical rotation of chiral compounds was determined on a Perkin-Elmer (Waltham, MS, USA) 241 MC polarimeter. Compounds were dissolved in chloroform or methanol. Concentrations are given in g/100 mL. 

### 2.10. Sonication

For ultrasonication a Branson Sonifier W-450-Digital (Branson Ultrasonics Corporation, Danbury, CT, USA) was used with a 1/2′ tip. For ultrasonication in a sonication bath, a Bandelin Sonorex RK52H from Bandelin electronic (Berlin, Germany) was used. 

### 2.11. Light Scattering and Zeta Potential 

DLS measurements have been performed in technical triplicates on a Malvern Zetasizer Nano S90 (Malvern Panalytical GmbH, Kassel, Germany) at an angle of 90°.

Zeta potential has been measured in technical triplicates on a Malvern Zetasizer NanoZ (Malvern Panalytical GmbH). Nanocapsule dispersions were diluted in a potassium chloride solution (1 mM, 25 °C, pH 6.8) prior to measurement. 

### 2.12. Transmission Electron Microscopy (TEM)

TEM was performed by placing a drop of diluted nanocapsule dispersion onto a 300-mesh carbon-coated copper grid and drying under ambient conditions. TEM measurements were operated on a Jeol 1400 transmission electron microscope from Jeol GmbH (Freising, Germany).

### 2.13. Fluorescence Intensity Measurements

Fluorescence intensity measurements were operated in 96-well plates on the Infinite M1000 plate reader from Tecan (Männedorf, Switzerland). Measurements were performed with two dilutions in duplicates each. 

### 2.14. Flow Cytometry and Confocal Laser Scanning Microscopy

For flow cytometry analysis, cells were detached and measurements were performed on an Attune NxT flow cytometer (Thermo Fisher). Confocal laser scanning microscopy was performed on a Zeiss LSM 710 NLO from Carl Zeiss Microscopy GmbH (Jena, Germany).

### 2.15. Experimental Procedures and Compound Characterization

Detailed synthetic procedures are given within the Supplementary Material.

### 2.16. Generation of Immature or Mature Dendritic Cells

All experiments were performed in compliance with the relevant laws and institutional guidelines. The institutional ethics committee approved the study (Landesärztekammer Rheinland-Pfalz, 837.439.12 (8540-F), approved on 02.01.2017). Written informed consent was obtained for any experimentation we carried out when using samples from human subjects. As mentioned above, the studies were conducted in full accordance with the Declaration of Helsinki.

Dendritic cells were generated from monocytes using plastic adherence. PBMC were seeded at a density of 5 × 10^6^ cells per well of a 24-well plate. Cells were allowed to adhere in a humidified environment at 37 °C with 5% CO_2_ for 1 h in 1 mL per well of DC-medium (RMPI with 2% heat-inactivated pooled human serum (HS), 100 U/mL penicillin (Gibco™, Fisher Scientific GmbH, Schwerte, Germany), and 100 mg/mL streptomycin (Gibco). By multiple washing with phosphate-buffered saline (PBS, Invitrogen™, Fisher Scientific GmbH, Schwerte, Germany), non-adhering cells were removed. Monocytes were kept in DC-medium containing 100 ng/mL granulocyte-macrophage-colony stimulating factor (GM-CSF, PeproTech, Cranbury, NJ, USA) and 100 ng/mL interleukin-4 (IL-4, PeproTech, Cranbury, NJ, USA) at 37 °C/5% CO_2_ for two days. On day 3, immature dendritic cells (iDC) were harvested by washing once with PBS and incubating cells with PBS/EDTA (2 mM) at 4 °C for 10 min. Prior to experiments, cells were examined for the expression of CD11c and CD86 by employing flow cytometry.

### 2.17. Loading of DC with Nanocapsules

1 × 10^5^ DCs were incubated with NC at 37 °C/5% CO_2_ in DC medium supplemented with 1600 U/mL GM-CSF and 500 U/mL IL-4. Two different nanocapsule concentrations (37.5 or 75 μg/mL) were analyzed after an incubation period of 2 h. Afterwards, cells were collected using multiple washing steps with cold PBS. The nanocapsule containing supernatant was removed by centrifugation for 5 min at 470 g.

### 2.18. Flow Cytometry Analysis of NC-Loaded DC

Pelleted DCs were stained with CD11c-FITC (Miltenyi Biotec B.V. & Co. KG, Bergisch Gladbach, Germany), CD45-APC-Cy7 (BD Biosciences, San Jose, CA, USA) and CD45-V450 (BD Biosciences, San Jose, CA, USA). Cells were allowed to incubate with the various antibodies for 15 min at 4 °C. After a washing step with PBS/0.1 % BSA, the cells were centrifuged and resuspended in 300 μL PBS/0.1% BSA. NC uptake by DC was detected using an Attune NxT flow cytometer (Thermo Fisher, Waltham, MS, USA), selecting the cells with an FSC/SSC plot, thereby excluding cell debris. Fluorescence of the NC dye sulforhodamine (SR101) was detected using a 586/15 nm bandpass filter with excitation by a 100 mW/561 nm laser.

### 2.19. Statistical Methods

For the cell uptake experiments, the program GraphPad Prism 5 (GraphPad Software, San Diego, CA, USA) was used. Data analysis was conducted using two-way analysis of variance (ANOVA). The cell uptake experiments were performed in duplicates. Two different nanocapsule batches were used, of which one batch is described in Figures 3 and 4 of the main manuscript and the second one in Figure S32 (Supplementary Material).

## 3. Results

**Figure 1:**
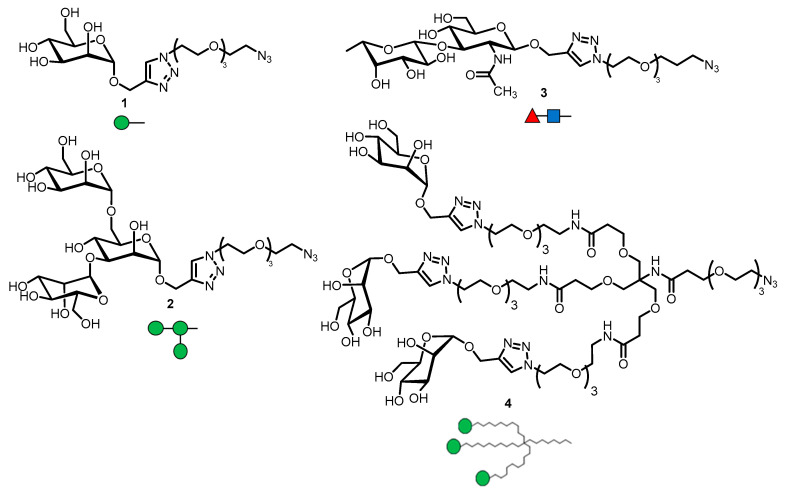
Chemical structures of selected carbohydrate ligands for receptor dendritic cell-specific intercellular adhesion molecule-3-grabbing non-integrin (DC-SIGN) targeting and corresponding symbol nomenclature adopted by the consortium for functional glycomics [28].

It is known that DCs recognize different carbohydrates and complex glycan structures through their DC-SIGN-receptor [23,24]. A key study by Holla and Skerra revealed that simple mannose-units, and especially branched oligomannosidic structures, in particular a trimannose structure (3,6-di-(α-d-mannopyranosyl)-α-d-mannopyranose) as well as fucose-containing saccharides, in particular a disaccharide (3-(β-l-fucopyranosyl)-2-acetamido-2-deoxy-β-d-glucopyranose), are recognized by DC-SIGN [25]. Based on these observations, we selected four different potential ligands for DC-SIGN that carry a poly(ethylene glycol) (PEG)-based, azide-terminated linker to enable covalent binding through azide-alkyne click reactions to nanocarriers as the target structures for total synthesis (Figure 1) A simple d-mannose monosaccharide **1** and the mentioned trimannose structure **2**, which were available from earlier studies [4,26,27], as well as a fucose containing disaccharide **3** and a mannose-terminated glycodendron **4**, were chosen as potential ligands. 

**Scheme 1:**
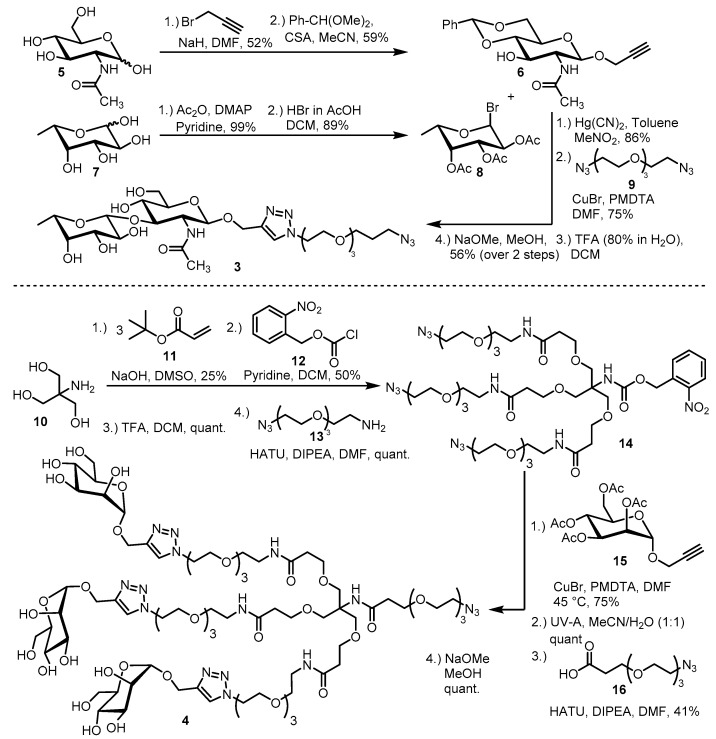
Synthesis of the disaccharide **3** from *N*-acetylglucosamine (**5**) and l-fucose (**7**) over eight steps with (top) and synthesis of the mannose glycodendron **4** from Tris (**10**) over seven steps (bottom).

The synthesis of the fucose disaccharide **3** started from *N*-acetylglucosamine (**5**), which was deprotonated at the most acidic 1-OH position and then was β-selectively alkylated with progargyl bromide in 52% yield (Scheme 1, top) [29]. The 4- and 6-positions were then benzylidene-protected in 59% yield through reaction with benzaldehyde dimethyl acetal to give derivative **6** over two steps. Next, l-fucose (**7**) was peracetylated in 99% yield and then transformed into the glycosyl bromide **8** in 89% using HBr in acetic acid. Building blocks **6** and **8** were then combined under Helferich conditions using Hg(CN)_2_ in toluene and MeNO_2_ to furnish the disaccharide in 86% yield [30]. This was followed by the introduction of the azide PEG-linker **9** in 86% yield using conditions for the CuAAC-reaction (copper-catalyzed alkyne-azide cycloadditon) with CuBr and PMDTA (*N*,*N*,*N’*,*N’’*,*N’’*-pentamethyldiethylenetriamine) as the ligand [31]. Finally, the benzylidene acetal was removed using TFA followed by global deprotection under Zemplén conditions to furnish the desired disaccharide **3** over six linear steps with 11% total yield.

After successful synthesis of disaccharide 3, the synthesis of the desired mannose glycodendron **4** was addressed. The dendron core was constructed starting from Tris (**10**, tris(hydroxymethyl)aminomethane), which was polyalkylated using *tert*-butyl acrylate (**11**) in 25% yield. Then, the amino group was protected in 50% yield using *o*-nitrobenzyl chloroformate, which was synthesized from *o*-nitrobenzyl alcohol upon reaction with triphosgene in situ (see the SI for more details). The ester moieties were cleaved using TFA and then functionalized with amine-azide linker **13** to give the dendron core **14** under standard amide coupling conditions in quantitative yield [32]. Mannose alkyne **15**, which was synthesized according to a previous report [26], was coupled to the dendron core **14** using the previously mentioned CuAAC conditions in 75% yield. The tertiary amine in the dendron core was then deprotected using a photochemical cleavage of the *o*-nitrobenzyl moiety upon irradiation with UV-A light [33]. Previous efforts with other protecting groups suffered from either poor reproducibility or poor yield upon deprotection, which underlines the mildness and efficiency of this light-induced protecting group cleavage. The obtained amine was then functionalized with another PEG-linker **16** in 41% yield followed by global deprotection to furnish the desired glycodendron **4** over eight linear steps in 4% overall yield.

**Figure 2:**
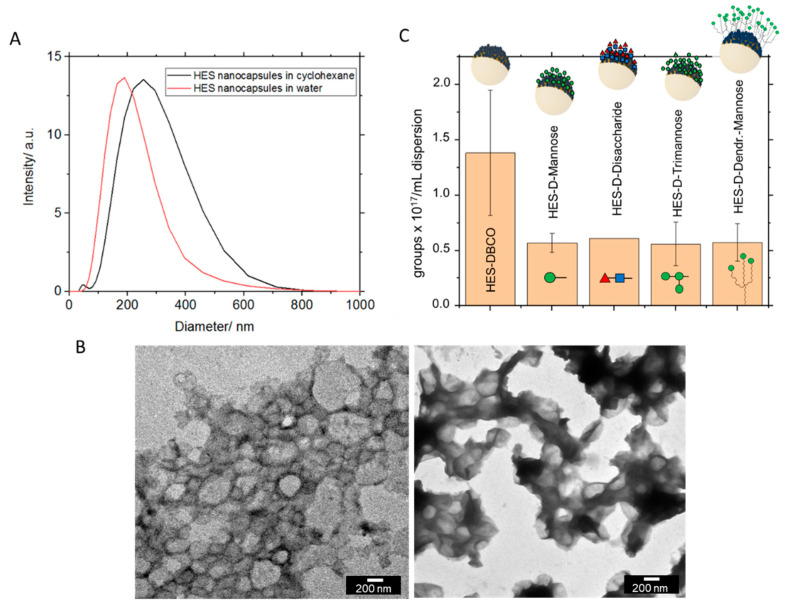
(**A**) Average size distribution of four hydroxyethyl starch (HES) nanocapsule batches in cyclohexane and in water measured at an angle of 90°. (**B**) TEM micrograph of HES nanocapsules in cyclohexane (left) and in water (right). (**C**) Quantification of free dibenzocycloocytne (DBCO) groups and DBCO groups clicked to functionalized carbohydrates per mL dispersion by a fluorescent assay.

After the synthesis of the DC-SIGN ligands had been accomplished, hydroxyethyl starch (HES) nanocapsules were prepared through interfacial crosslinking of toluene diisocyanate (TDI) with HES in inverse miniemulsion [23,34,35,36]. Isocyanate groups of TDI reacted in a polyaddition reaction with hydroxyl groups of HES, resulting in the formation of a polyurethane shell [37]. We obtained nanocapsules with a relative diameter of 250 nm in cyclohexane. High batch-to-batch reproducibility was ensured. For further modification and biological testing, the nanocapsules were redispersed in aqueous sodium dodecyl sulfate (SDS) solution. The hydrodynamic diameter of the nanocapsules in aqueous solution decreased to approximately 200 nm (Figure 2A). Furthermore, TEM micrographs proved the core-shell morphology of the obtained nanocapsules in cyclohexane (Figure 2B, left) as well as in aqueous medium (Figure 2B, right).

During redispersion of the nanocapsules in water, residual isocyanate groups hydrolyze to amino groups, which were then reacted with DBCO-PEG_4_-NHS ester. The so-attached dibenzocyclooctyne (DBCO)-groups were exploited for copper-free click reaction with the synthesized carbohydrate ligands **1**–**4**. Thus, a defined surface modification with DBCO groups can be achieved in order to covalently bind as many carbohydrate molecules as possible. This is important because it is known that single carbohydrates show rather low influence on the immune system, while multivalent presentation of carbohydrates exhibits strong immunological properties, as mentioned in the introduction [10,11]. For quantification of DBCO groups on the nanocapsules, a fluorescent assay was used and the average DBCO quantity was found to be 1.38 × 10^17^ DBCO groups per mL dispersion after coupling the DBCO-PEG_4_-NHS ester to the nanocapsules (10 mg nanocapsules per mL) [36]. Then, we added a three-fold molar excess of azide-containing carbohydrates with respect to previously detected DBCO groups to the DBCO-functionalized nanocapsules. After three days, the remaining DBCO groups were quantified (Figure 2C). We determined 20% of the initial DBCO groups. The decrease in the DBCO can be attributed to the reaction of the azide, but additionally to the chemical sensitivity of the DBCO molecule to other nucleophiles [37]. As a control, pure DBCO capsules showed a 36% decrease over time. Therefore, around 42% of the DBCO groups are coupled to carbohydrates. This is independent of the carbohydrate added.

The relative size of the nanocapsules with functionalization and the surface charge is shown in Table 1. The surface modification of the nanocapsules caused an increase in the hydrodynamic diameter of the capsule. This is particularly noticeable with the bulky dendron. All samples, after coupling, still showed a negative ζ-potential.

After successful binding of the carbohydrates to the nanocapsules, cell uptake experiments were performed. Therefore, human dendritic cells (hDCs) were incubated with DBCO-modified HES nanocapsules (DBCO), with HES-DBCO nanocapsules surface modified with four different carbohydrates (mannose 1, disaccharide 2, trimannose 3 and dendron-mannose 4) and with pure cell medium as negative control (NC). 

**Figure 3:**
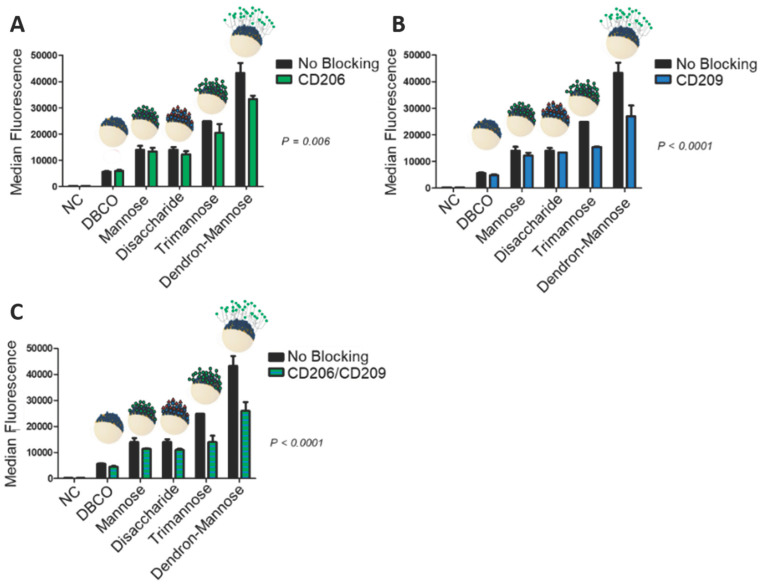
Uptake and blocking experiments of HES nanocapsules, decorated with carbohydrates into human dendritic cells (hDCs) in three different experimental setups. The experiments were performed in duplicates using ANOVA for data evaluation with a *p*-value = 0.006 (**A**), *p*-value < 0.0001 (**B**) and *p*-value < 0.0001 (**C**).

All nanocapsules were fluorescently labelled with the dye sulforhodamine. After incubation and washing, flow cytometry experiments were performed. The uptake behavior of the differently functionalized nanocapsules into hDCs is shown for the different experimental setups (see Figure 3).

**Figure 4:**
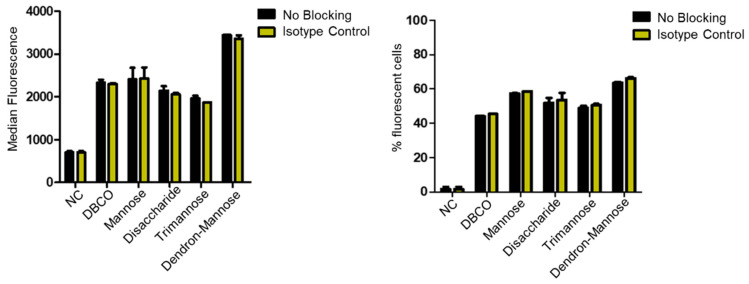
Flow cytometry analysis of hDCs which were treated with an IgG isotype control prior to incubation with differently modified HES nanocapsules. The experiments were performed in duplicates using ANOVA for data evaluation with a *p*-value = not significant.

Next, we performed surface expression control of the hDCs and an isotype control (Figure 4). hDCs were incubated with specific antibodies against CD206, CD209 and CD207 (langerin receptor), excess antibody was washed off, and the cells were analyzed by flow cytometry.

## 4. Discussion

In general, we observed an increased uptake for the carbohydrate functionalized HES nanocapsules into hDCs. In contrast, a desirably low unspecific uptake of DBCO-modified HES nanocapsules into the cells was detected. The amount of nanocapsules taken up into hDCs was found to be dependent on the structures of the respective carbohydrate presented on the nanocapsule surface. As can be seen from Figure 3, an increasing complexity of the carbohydrate ligand enhances the cell uptake of the surface-modified nanocapsules. Nanocapsules with attached mannose 1 were shown to have the weakest uptake. It was to our surprise that disaccharide 2 showed similar results, which was not expected from the literature, where an increased binding for this fucose disaccharide 3 over the mono-mannose 1 was found [25]. The most complex ligand, trimannose 2, showed the highest uptake tendency in this series. The comparison of trimannose 3 and dendron-mannose- 4 revealed a strong difference and a nearly doubled uptake for the dendron-mannose 4. It is important to note that according to the retrospective DBCO quantification after carbohydrate attachment, a similar amount of residual DBCO was found on the different samples, independent of the structure of the added carbohydrate (Figure 3C). It is assumed that the size and the steric nature of the respective carbohydrate has a significant influence on the binding to the nanocapsule. Therefore, we expect that more of the respective carbohydrate is needed for a complete coverage of the nanocapsule with mannose 1 than for the coverage with dendron-mannose 4. Despite the associated molecular excess of mannose 1 on the nanocapsule, compared to dendron-mannose 4, the dendron-mannose decorated nanocapsule appeared to be superior in uptake. Since the dendron presents three mannose units per dendron coupled to the nanocapsules, we also reduced the dendron density by a factor of three in order to have the same mannose units per capsules as in the mono-mannose case (nanocapsules with 1/3 of dendron-mannose, *d* = 364 nm, *ζ* potential = 13 ± 2.6 mV). Independent of the amount of the dendron-mannose, we found an increased uptake into the cells (see Supporting Information). In the next step, we investigated if indeed specific receptors mediate the cell uptake. Therefore, we first treated hDCs with antibodies against CD206 and CD209 to block the receptors. In this case, we found a decrease in the uptake of carbohydrate-modified HES nanocapsules in comparison to the non-blocked scenario (Figure 2). We observed the strongest decrease in uptake for the trimannose 3 and dendron-mannose 4 modified nanocapsules, especially when the DC-SIGN receptor (CD209) was blocked or the blocking included both the mannose-receptor (CD206) and DC-SIGN. These findings suggest that the surface-modified nanocapsules undergo a receptor-mediated uptake into hDCs. This is remarkable since it has been postulated in the literature that DCs recognize mannose units through the mannose receptor CD206 [38], while branched oligomannosidic structures, in particular trimannose 2, are internalized through DC-SIGN (CD209) [39,40]. However, the observation that simple mono-mannose containing glycodendron 4 showed a strong decrease in uptake through blocking of DC-SIGN contradicts this hypothesis and indicates that a multivalent binding of ligands to DC-SIGN might play a bigger role than the (branched) structural complexity of oligosaccharides. This finding underlines the initially mentioned importance of multivalent addressing of lectins through carbohydrates.

The possibility of a receptor-mediated uptake mechanism was further investigated using a surface expression control of the hDCs and an isotype control (Figure 4). We found that approximately 77% of the hDCs population expressed CD206, 73% expressed CD209 and 98% expressed CD207 (see the SI for more details). These results verify that a sufficient number of targeted receptors were present on the surface of the hDCs. Furthermore, it was observed that when incubation of the cells was performed with an unspecific IgG antibody (isotype control), the uptake of the different nanocapsules did not differ from the non-blocked cells (Figure 4). The results demonstrate that there is a high probability for receptor-mediated uptake of carbohydrate functionalized HES nanocapsules.

## 5. Conclusions

In summary, a series of mono- and oligosaccharide ligands for DC-SIGN were prepared and covalently coupled to hydroxyethyl starch nanocapsules through copper-free click chemistry. The cell uptake in human dendritic cells was investigated and revealed that a multivalent glycodendron is superior in cell uptake to the 3,6-branched trimannose. Additionally, blocking experiments revealed that mannose as well as the mannose glycodendron addressed DCs preferentially through CD209 rather than through CD206, which underlines that the selectivity for branched mannose oligosaccharides, might be traced back to multivalent presentation of saccharides in these structures rather than to structural complexity. This initial discovery opens the way to the development of simpler multivalent ligands rather than branched and structurally complex oligosaccharides to achieve effective DC-SIGN targeting in future work.

## Figures and Tables

**Table 1:** Physicochemical properties of carbohydrate-modified HES nanocapsules. Average diameters were determined at a scattering angle of 90°.

Sample	Average Diameter/nm	ζ-Potential/mV
HES-DBCO	289 ± 26	−7.9 ± 6.8
HES-DBCO-Mannose	418 ± 105	−14.9 ± 4.1
HES-DBCO-Disaccharide	392 ± 41	−11.9 ± 3.2
HES-DBCO-Trimannose	514 ± 135	−10.1 ± 6.0
HES-DBCO-Dendron-Mannose	544 ± 54	−13.8 ± 1.1

## Supplementary Materials

The following are available online at https://www.mdpi.com/2073-4409/9/9/2087/s1. Part I: Experimental Procedures & Compound Characterization; Part II: Nanocapsule synthesis and functionalization; Part III: NMR Spectra; Part IV: Flow Cytometry Data; Part V: Confocal Laser Scanning Microscopy (cLSM).
