# Supplementary material for: Multivalency Beats Complexity: A Study on the Cell Uptake of Carbohydrate Functionalized Nanocarriers to Dendritic Cells
Source: Cells. 2020 Sep 12;9(9):2087. doi: 10.3390/cells9092087 (PMC7564404; doi:10.3390/cells9092087)
Supplement: Supplementary file 1 [file cells-09-02087-s001.pdf]

Supplementary Material

I. Experimental Procedures & Compound Characterization

II.1 Preparation of the clickable dendrimer core

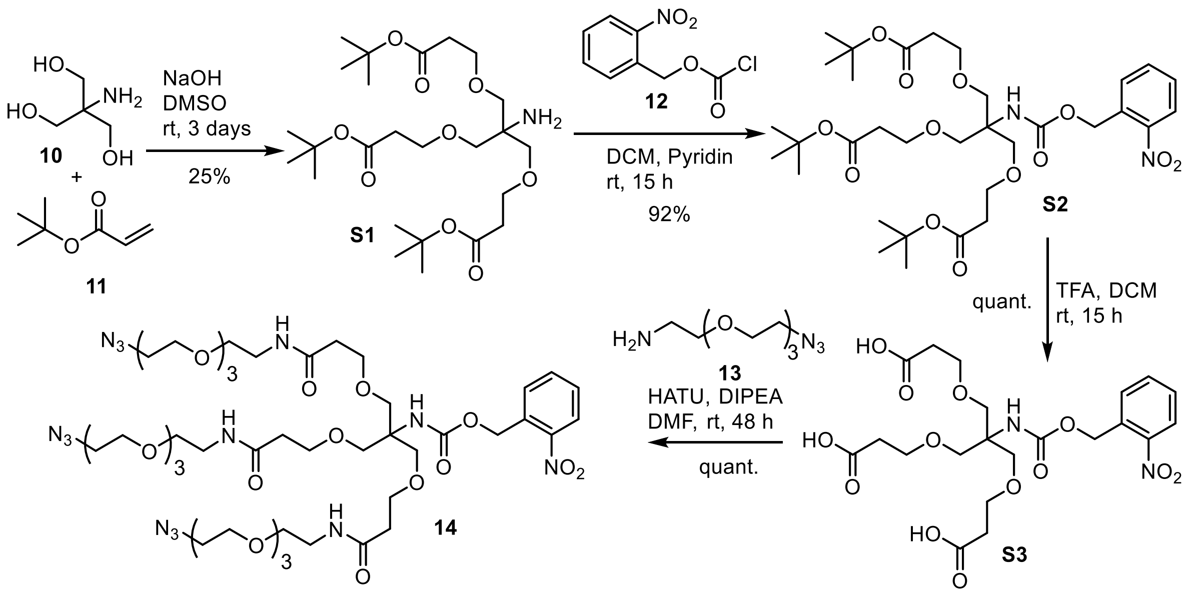

Figure S1: Synthesis overview of the clickable dendrimer core **14**.

Tris((2-(tert-butoxycarbonyl)ethoxyl)methyl)methylamine (S1)

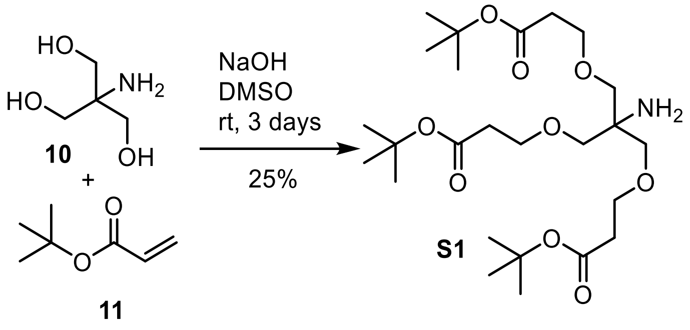

Figure S2: Synthesis of 6-Amino-6-(1-*tert*-butyl-4-oxapentanoat-5-yl)-4,8-dioxaundecan-1,11-dicarboxylicacid-di-*tert*-butylester (**S1**).

This compound was synthesized after a modified procedure by *Appel et al.*[1]

To a solution of tris(hydroxymethyl)aminoethane (**10**, 7.50 g, 0.06 mol, 1.0 eq.) in DMSO (12.50 mL) was added a solution of sodium hydroxide ($5M_{aq}$, 2.00 mL). To the resulting suspension was added dropwise *tert*-butyl acrylate (**11**, 30.8 mL, 0.21 mol, 3.5 eq.) while cooling. The reaction vessel was covered with aluminum foil to shield the reaction mixture from light, as described by *Appel et al*. The reaction mixture was stirred for 2 days at room

temperature. Reaction control by LC-MS revealed complete conversion of the starting material. All volatiles were removed in vacuo and the residue was taken up in diethyl ether and extracted with water and brine. The aqueous layer was extracted with diethyl ether (2x). The organic layers were combined, dried over Na_2SO_4, filtered and all volatiles were removed in vacuo. The crude product was purified by column chromatography (^{c}Hex/EtOAc + 5%$_{v/v}$ Et_3N). The product was obtained as a yellow oil.

Yield: 13.8 g (0.03 mol, 44 %), (Lit.)[1]: 33 %, yellow oil.

***R$_f$*:** = 0.33 (^{c}Hex/EtOAc 3:1 + 5%$_{v/v}$ NEt_3).

$C_{25}H_{47}NO_9$ (M = 505.33 g/mol).

IR(ATR): $\tilde{\nu}$ (cm^{-1}) = 2978, 2869, 1727, 1479, 1458, 1366, 1253, 1154, 1107, 1068, 847, 755, 733.

ESI-MS: m/z (%) = 506.4 (100) $[M+H]^+$.

^{1}H-NMR, COSY (300 MHz, $CDCl_3$): δ (ppm) = 3.55 (t, *J* = 6.4 Hz, 6H, H-3), 3.22 (s, 6H, H-2), 2.36 (t, *J* = 6.4 Hz, 6H, H-4), 1.35 (s, 27H, H-7).

^{13}C-NMR, HSQC, HMBC (75 MHz, $CDCl_3$): δ (ppm) = 170.8 (C-5), 80.3 (C-6), 72.8 (C-2), 67.1 (C-3), 55.9 (C-1), 36.3 (C-4), 28.1 (C-7).

The analytical data are consistent with those reported in the literature.[1]

o-Nitrobenzyl-(6-(1-*tert*-butyl-4-oxapentanoat-5-yl)-4,8-dioxa-1,11-di-*tert*-butylundecanoat-6-yl)-carbamate (S2)

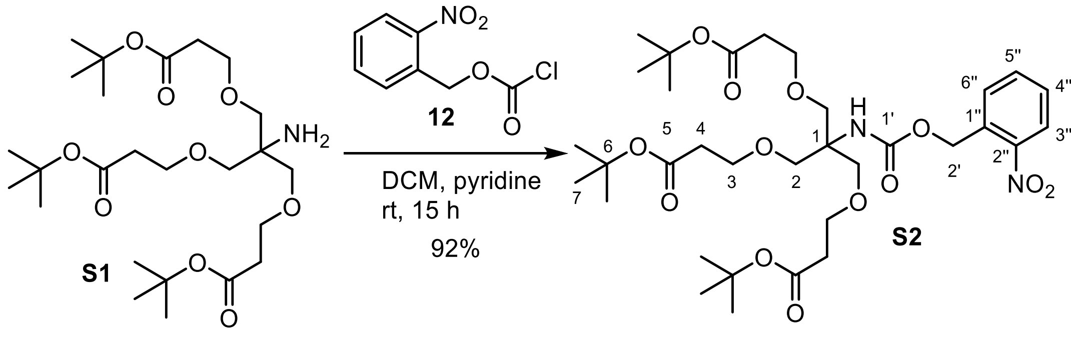

Figure S3: Synthesis of *o*-Nitrobenzyl-(6-(1-*tert*-butyl-4-oxapentanoat-5-yl)-4,8-dioxa-1,11-di-*tert*-butylundecanoat-6-yl)-carbamate (**S2**).

This compound was synthesized after a modified procedure by *Tsai et al.*[2].

The amine **S1** (4.00 g, 7.92 mmol, 1.00 eq.) was dissolved in DCM (25.0 mL) and pyridine (3.00 mL) under argon atmosphere. *o*-Nitro-benzylchloroformate (**12**) was prepared by dissolving 2-nitrobenzyl alcohol (2.00 g, 13.1 mmol, 1.65 eq.) in DCM (20.0 mL) and pyridine (1.00 mL) under argon atmosphere. The yellow solution was cooled to -78 °C and a solution of

triphosgene (1.28 g, 4.37 mmol, 1.00 eq.) in DCM (30.0 mL) was added. The reaction mixture was stirred for 2 days. The crude solution of *o*-nitrobenzylchloroformate was taken up by syringe and added dropwise to the solution of amine **3**. The reaction was stirred for 14 h. While stirring precipitation of a colorless solid was observed. After completion of the reaction all volatiles were removed in vacuo. The crude product was purified by column chromatography (^{c}Hex/EtOAc, Isolera Flash Purification System). The product was obtained as a yellow oil.

Yield: 2.69 g (3.93·mmol, 50 %), (Lit.)[2]: 55 %.

***R**$_f$*: = 0.23 (^{c}Hex/EtOAc 5:1).

$C_{33}H_{52}N_2O_{13}$ (M = 684.35 g/mol).

IR(ATR): ṽ (cm^{-1}) = 2978, 2932, 2876, 1728, 1529, 1367, 1253, 1158, 1111, 1079, 874, 732.

ESI-MS: m/z (%) = 707.4 (61.5) $[M+Na]^+$.

HR-MS: m/z = calculated for $[C_{33}H_{52}N_2O_{13}+Na]^+$: 707.3367, found: 707.3360.

^{1}H-NMR, COSY (300 MHz, $CDCl_3$): δ (ppm) = 8.12–8.09 (m, 1H, H-3''), 7.68–7.62 (m, 2H, H-5'',H-6''), 7.49–7.38 (m, 1H, H-4''), 5.51 (s, 1H, N-*H*), 5.46 (s, 2H, H-2'), 3.68–3.60 (m, 12H, H-2, H-3), 2.44 (t, *J* = 6.3 Hz, 6H, H-4), 1.43 (s, 27H, H-7).

^{13}C-NMR, HSQC, HMBC (75 MHz, $CDCl_3$): δ (ppm) = 171.0 (C-5), 171.0 (C-1'), 154.6 (C-1'), 147.1 (C-2''), 134.1 (C-1''), 133.9 (C-5''), 128.4 (C-6''), 128.3 (C-4''), 125.0 (C-3''), 80.6 (C-6), 69.4 (C-2), 67.2 (C-3), 62.8 (C-2'), 58.9 (C-1), 36.3 (C-4), 28.2 (C-7).

o-Nitrobenzyl-(6-(1-carboxy-4-oxapentan-5-yl)-4,8-dioxa-1-11-dicarboxyundecan-6-yl)-carbamate (S3)

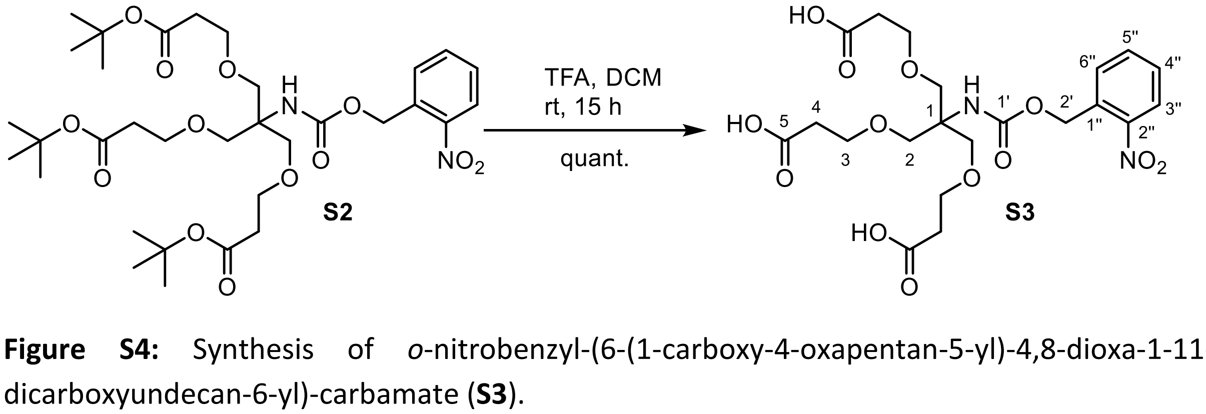

Figure S4: Synthesis of *o*-nitrobenzyl-(6-(1-carboxy-4-oxapentan-5-yl)-4,8-dioxa-1-11-dicarboxyundecan-6-yl)-carbamate (**S3**).

This compound was synthesized after a modified procedure by *Landeros et al*.[3]

Ester **S2** (2.16 g, 3.16 mmol, 1.00 eq.) was dissolved in DCM (11.0 mL) under argon atmosphere. TFA (1.95 mL, 0.03 mmol, 10.0 eq.) was added dropwise to the solution. The reaction mixture was stirred for 2 days at room temperature. Reaction control by TLC and

LC-MS indicated complete conversion of the starting material. All volatiles were removed in vacuo and the crude product was purified by column chromatography (^{c}Hex/EtOAc + 1%$_{v/v}$ HCOOH). The product was obtained as a light-yellow oil.

Yield: 1.63 g (3.16 mmol, quant.), (Lit.)[3]: 94 %, light yellow oil.

R$_f$: = 0.31 (^{c}Hex/EtOAc 1:2 + 1% HCOOH).

$C_{21}H_{28}N_2O_{13}$ (M = 516.16 g/mol).

IR(ATR): $\tilde{\nu}$ (cm^{-1}) = 2924, 2882, 1713, 1525, 1343, 1238, 1194, 1097, 1071, 909, 731.

ESI-MS: m/z (%) = 517.2 (100) [M+H]$^+$.

HR-MS: m/z = calculated for [$C_{21}H_{28}N_2O_{13}$+H]$^+$: 517.1669, found: 517.1660.

^{1}H-NMR, COSY (600 MHz, DMSO-d$_6$): δ (ppm) = 8.14–8.12 (m, 1H, H-3''), 7.82–7.79 (m, 1H, H-5''), 7.68–7.67 (m, 1H, H-6''), 7.61–7.58 (m, 1H, H-4''), 6.89 (s, 1H, N-*H*), 5.34 (s, 2H, H-2'), 3.56 (t, *J* = 6.3 Hz, 6H, H-3), 3.49 (s, 6H, H-2), 2.42 (t, *J* = 6.3 Hz, 6H, H-4).

^{13}C-NMR, HSQC, HMBC (151 MHz, DMSO-d$_6$): δ (ppm) = 172.8 (C-5), 154.2 (C-1'), 146.8 (C-2''), 134.2 (C-5''), 133.4 (C-1''), 128.8 (C-4''), 128.4 (C-6''), 124.9 (C-3''), 68.0 (C-2), 66.8 (C-3), 61.7 (C-2'), 59.0 (C-1), 34.7 (C-4).

o-Nitrobenzyl-(1,35-diazido-18-(17-azido-5-oxo-2,9,12,15-tetraoxa-6-azaheptadecyl)-13,23-dioxo-3,6,9,16,20,27,30,33-octaoxa-12,24-diazapentatriacontan-18-yl)-carbamate (14)

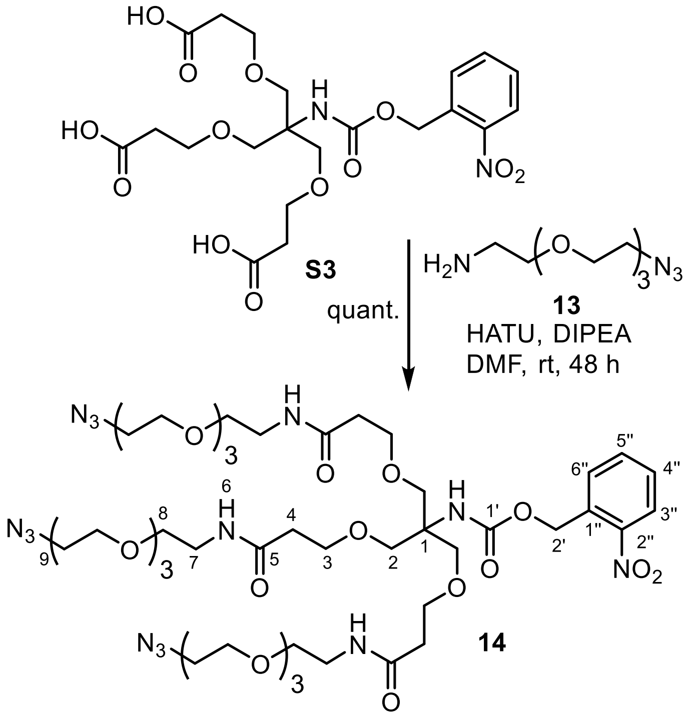

Figure S5: Synthesis of *o*-nitrobenzyl-(1,35-diazido-18-(17-azido-5-oxo-2,9,12,15-tetraoxa-6-azaheptadecyl)-13,23-dioxo-3,6,9,16,20,27,30,33-octaoxa-12,24-diazapentatriacontan-18-yl)-carbamate (**14**).

The synthesis was performed after a modified procedure by *Appel et al.*[1]

The carboxylic acid **S3** (1.60 g, 3.10 mmol, 1.00 eq.) was dissolved in anhydrous DMF (40.0 mL) under an atmosphere of argon. HATU (3.45 g, 9.30 mmol, 3.00 eq.) and DIPEA (2.12 mL, 12.4 mmol, 4.00 eq.) was added and the reaction mixture was stirred for 30 min at room temperature. Afterwards the amine **13** (2.71 g, 12.4 mmol, 4.00 eq.) dissolved in anhydrous DMF (8.00 mL) was added dropwise while stirring. The reaction mixture was stirred at room temperature for 2 days. Reaction control by TLC and LC-MS indicated complete conversion of the starting material. All volatiles were removed in vacuo and the residue was co-distilled with toluene (3x). The residue was purified by column chromatography (EtOAc/MeOH 7:3).

Yield: 3.46 g (3.16 mmol, quant.), (Lit.)[1]: 61 %, colorless oil.

***R_f*:** = 0.45 (EtOAc/MeOH 7:3).

$C_{45}H_{76}N_{14}O_{19}$ (M = 1116.54 g/mol).

IR(ATR): $\tilde{\nu}$ (cm^{-1}) = 2875, 2108, 1725, 1653, 1528, 1472, 1346, 1286, 1095, 843, 558.

ESI-MS: m/z (%) = 559.4 (100) $[M/2+H]^+$,1117.5 (49.5) $[M+H]^+$.

HR-MS: m/z = calculated for $[C_{45}H_{76}N_{14}O_{19}+Na]^+$: 1139.5308, found: 1139.5283.

^{1}H-NMR, COSY (400 MHz, $CDCl_3$): δ (ppm) = 8.12–8.06 (m, 1H, H-3''), 7.69–7.62 (m, 2H, H-5'', H-6''), 7.46–7.38 (m, 1H, H-4''), 6.68 (t, *J* = 5.5 Hz, 3H, -(C-5=O)-N*H*-), 5.69 (s, 1H, -(C-1'=O)-N*H*-), 5.45 (s, 2H, H-2'), 3.78–3.57 (m, 42H, H-2, H-3, H-8, -$CH_{2\text{-PEG}}$), 3.54 (t, *J* = 5.5 Hz, 6H, H-8), 3.42 (q, *J* = 5.5 Hz, 6H, H-7), 3.39 (t, 6H, H-9), 2.41 (t, *J* = 5.9 Hz, 6H, H-4).

^{13}C-NMR, HSQC, HMBC (101 MHz, $CDCl_3$): δ (ppm) = 171.3 (C-5), 154.7 (C-1'), 147.4 (C-2''), 134.0 (C-1''), 128.9 (C-6''), 128.6 (C-4''), 125.1 (C-2''), 70.8, 70.7, 70.6, 70.3 (each -$CH_{2\text{-PEG}}$), 70.1 (C-8), 70.0, 69.4 (-CH_2-PEG), 67.6 (C-3), 63.0 (C-2'), 59.1 (C-1), 50.8 (C-9), 39.3 (C-7), 36.8 (C-4).

II.2 Synthesis of the PEG spacers

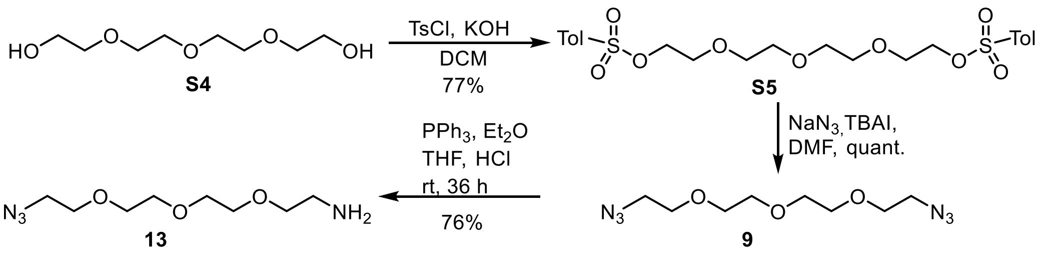

Figure S6: Synthesis of 1-azido-2-(2-(2-(2-azidoethoxy)ethoxy)ethoxy)ethane (**13**).

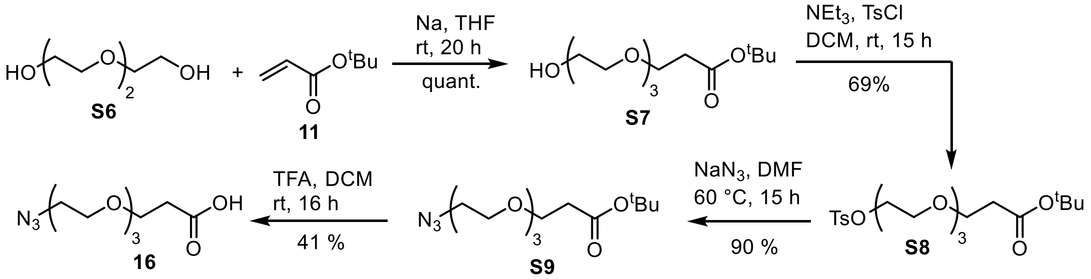

Figure S7: Synthesis of 12-azido-4,7,10-trioxadodecanic acid (**16**) over four steps.

1-Methanesulfonyl-2-(2-(2-(2-methanesulfonylethoxy)ethoxy)ethoxy)ethane (S5)

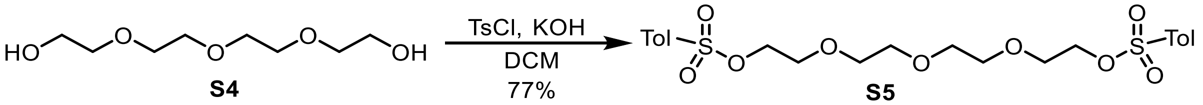

Figure S8: Synthesis of 1-methanesulfonyl-2-(2-(2-(2-methanosulfonylethoxy)ethoxy)-ethoxy)ethane (**S5**).

A solution of tetraethylene glycol (**S4**, 26.7 mL, 0.16 mol, 1.0 eq.) in abs. DCM (150.0 mL) was cooled to 0 °C. *p*-Toluenesulfonylchloride (58.9 g, 0.31 mol, 2.0 eq.) was added. The reaction mixture was stirred for 45 min until all starting material was completely dissolved. Finely ground potassium hydroxide (69.4 g, 1.24 mol, 7.8 eq.) was added in small portions over 10 min. The reaction mixture was stirred at room temperature for 4 h and reaction control via TLC indicated complete conversion. The reaction mixture was diluted with DCM and extracted with water (ice-cold) and brine. The organic layer was dried over Na_2SO_4, filtered and all volatiles were removed in vacuo. The crude product was purified by column chromatography (^{c}Hex/EtOAc 5:1 to 0:1) and the product was obtained as a colorless oil.

Yield: 58.385 g (123.03 mmol, 77%), (Lit.)[4] quant., colorless oil.

R_f: = 0.28 (^{c}Hex/EtOAc 1:1).

$C_{20}H_{26}O_9S_2$ (M = 474.54 g/mol).

IR(ATR): $\tilde{\nu}$ (cm^{-1}) = 2872, 1597, 1451, 1351, 1292, 1188, 1173, 1095, 1011, 913, 814, 772, 689.

ESI-MS: m/z (%) = 503.3 (100) $[M+H]^+$, 526.3 (12.4) $[M+Na]^+$.

^{1}H-NMR, COSY (300 MHz, $CDCl_3$): δ (ppm) = 7.81-7.75 (m, 2H, H-2'), 7.36–7.30 (m, 2H, H-3'), 4.17-4.11 (m, 2H, H-1), 3.70-3.64 (m, 2H, H-2), 3.57-3.53 (m, 4H, H-3 und H-4), 2.43 (s, 3H, H-5').

^{13}C-NMR, HSQC, HMBC (75 MHz, $CDCl_3$): δ (ppm) = 144.9 (C-4'), 133.1 (C-1'), 130.0 (C-3'), 128.1 (C-2'), 70.8 (C-4), 70.7 (C-3), 69.4 (C-1), 68.9 (C-2), 21.8 (C-5').

The spectral data are in accordance with the literature.[4]

1-Azido-2-(2-(2-(2-azidoethoxy)ethoxy)ethoxy)ethane (9)

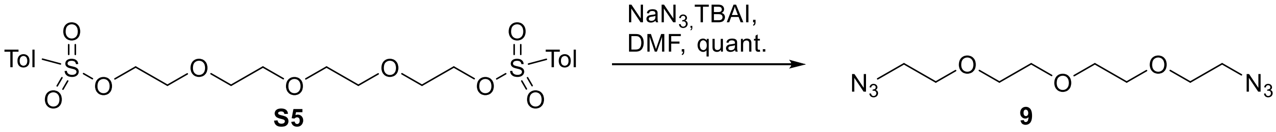

Figure S9: Synthesis of 1-azido-2-(2-(2-(2-azidoethoxy)ethoxy)ethoxy)ethane (**9**).

Tosylate (**S5**, 58.20 g, 115.80 mmol, 1.0 eq.), NaN_3 (18.82 g, 463.20 mmol, 2.5 eq.) and TBAI (2.13 g, 5.79 mmol, 5 mol%) were dissolved under argon atmosphere in anhydrous DMF (300.0 mL). The reaction mixture was stirred for 4 h at 80 °C. TLC indicated complete conversion and the reaction mixture was filtered over celite and all volatiles were removed *in vauo*. The residue was dissolved in Et_2O and filtered over celite. All volatiles were removed *in vauo* and the product **S5** was obtained as a yellow oil.

Yield: 28.343 g (116.04 mmol, quant.), yellow oil.

***Rf*:** = 0.60 (^{c}Hex/EtOAc 4:2).

$C_8H_{16}N_6O_3$ (M = 244.26 g/mol).

IR(ATR): $\tilde{\nu}$ (cm^{-1}) =2868, 2094, 1644, 1443, 1395, 1283, 1175, 1115, 925, 851, 831.

ESI-MS: m/z (%) = 267.2 (100) $[M+Na]^+$.

^{1}H-NMR, COSY (300 MHz, $CDCl_3$): δ (ppm) = 3.72–3.62 (m, 12H, 2 × N_3–CH_2–C*H*$_2$, 2 × C*H*$_2$–C*H*$_2$–O), 3.39 (t, ^3J = 5.1 Hz, 4H, N_3–C*H*$_2$).

^{13}C-NMR, HSQC, HMBC (75 MHz, $CDCl_3$): δ (ppm) = 70.9 (2 × *C*H$_2$–*C*H$_2$–O), 70.2 (2 × N_3–CH_2–*C*H$_2$), 50.8 (2 × N_3–*C*H$_2$).

The spectral data are in accordance with the literature.[5]

11-Amino-1-azido-3,6,9-trioxaundecane (13)

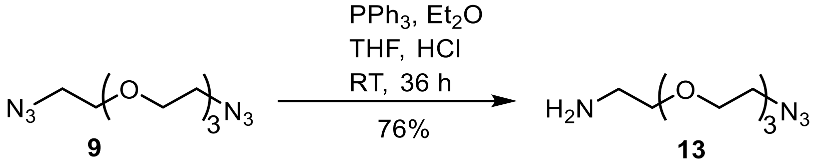

Figure S10: Synthesis of 11-amino-1-azido-3,6,9-trioxaundecane (**13**).

This compound was prepared after a modified procedure by *Davila et al.*[6] The diazide **9** (10.0 g, 0.04 mol, 1.00 eq.) was dissolved in a mixture of diethyl ether (72.3 mL), THF (15.0 mL) and HCl (1M, 60.0 mL). PPh_3 (10.8 g, 0.04 mol, 1.00 eq.) was dissolved in diethyl ether (60.0 mL) and added dropwise to the reaction mixture over 2 h while stirring was set to high rpm. The reaction mixture was stirred for 2 h at room temperature. Reaction control by TLC and LC-MS indicated complete conversion of the starting material. The reaction mixture was transferred into a separatory funnel and the layers were separated. The aqueous phase was extracted with diethyl ether (4x). The aqueous phase was treated with sodium hydroxide until the pH was basic. The aqueous phase was extracted with DCM (4x). The organic layer was dried over Na_2SO_4, filtered and all volatiles were removed in vacuo. The crude product was

purified by column chromatography (DCM/MeOH 20:1 + 1%$_{v/v}$ Et_3N). The product was obtained as a yellow oil.
Yield: 3.65 g (0.02 mol, 41 %), (Lit.)[6]: 81%, yellow oil.

***Rf*:** = 0.27 (DCM/MeOH 20:1 + 1%$_{v/v}$ NEt_3).

$C_8H_{18}N_4O_3$ (M = 218.14 g/mol).

IR(ATR): $\tilde{\nu}$ (cm^{-1}) = 2868, 2102, 1576, 1472, 1348, 1302, 1119, 1037, 938, 851, 821, 558.

ESI-MS: m/z (%) = 241.3 (100) $[M+Na]^+$.

^{1}H-NMR, COSY (300 MHz, $CDCl_3$) δ (ppm) = 3.80–3.55 (m, 10H, H-3, H-4, H-5, H-6, H-7), 3.49 (dd, *J* = 5.6, 4.8 Hz, 2H, H-1), 3.36 (dd, *J* = 5.6, 4.5 Hz, 2H, H-8), 2.85 (dd, *J* = 5.6, 4.8 Hz, 2H, H-2), 1.95 (s, 2H, -N*H*$_2$).

^{13}C-NMR, HSQC, HMBC (75 MHz, $CDCl_3$) δ (ppm) = 73.1 (C-1), 70.8 (-*C*H$_2$-), 70.7 (-*C*H$_2$-), 70.7 (-*C*H$_2$-), 70.3 (-*C*H$_2$-), 70.1 (-*C*H$_2$-), 50.7 (C-8), 41.7 (C-2).

The analytical data is in accordance with the literature.[6]

12-Hydroxy-4,7,10-trioxadodecansäure-*tert*-butylester (S7)

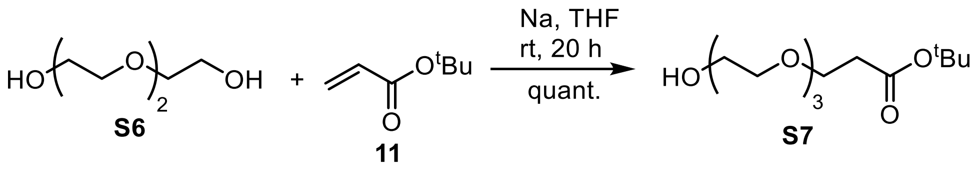

Figure S11: Synthesis of 12-Hydroxy-4,7,10-trioxadodecansäure-*tert*-butylester (**S7**).

This compound was synthesized after a modified procedure by *Tavernaro et al.*[7] Triethylene glycol (**S6**, 1.79 mL, 13.3 mmol, 3.30 eq.) was dried in vacuo for 2 h. Afterwards it was dissolved in anhydrous THF (10.0 mL) and sodium (3.00 mg, 0.13 mmol, 3mol%) and *tert*-butylacrylate (**11**, 0.68 mL, 4.00 mmol, 1.00 eq.) was added dropwise to the mixture. The reaction was stirred for 20 h at room temperature. Afterwards, HCl (1M, 0.5 mL) was added and the all volatiles were removed in vacuo. The residue was taken up in brine and extracted with EtOAc (4x). The organic layers were combined, dried over Na_2SO_4, filtered and all volatiles were removed in vacuo. The product was obtained as a colorless oil.

Yield: 1.11 g (4.00 mmol, quant.), (Lit.)[7]: 86 %, colorless oil.

***R$_f$*:** = 0.48 (^{c}Hex/EtOAc 1:1).

$C_{13}H_{26}O_6$ (M = 278.17 g/mol).

IR(ATR): $\tilde{\nu}$ (cm^{-1}) = 2249, 2124, 1053, 1025, 1006, 820, 758, 623.

ESI-MS: m/z (%) = 279.2 (100) $[M+H]^+$.

^{1}H-NMR, COSY (300 MHz, $CDCl_3$): δ (ppm) = 4.60-4.55 (m, 1H, -O*H*), 3.58 (t, *J* = 6.2 Hz, 2H, H-3), 3.53-3.44 (m, 10H, H-4, H-5, H-6, H-7, H-8), 3.43-3.38 (m, 2H, H-9), 2.41 (t, *J* = 6.2 Hz, 2H, H-2), 1.39 (s, 9H, H-11).

^{13}C-NMR, HSQC, HMBC (75 MHz, $CDCl_3$): δ (ppm) = 170.4 (C-1), 79.7 (C-10), 72.4 (C-9), 69.8 (C-7), 69.8 (C-6), 69.7 (C-5), 69.7 (C-4), 66.2 (C-3), 60.2 (C-8), 35.8 (C-2), 27.8 (C-11).

The analytical data are in accordance with the literature.[7]

12-(*p*-Toluenesulfonyl)-4,7,10-trioxadodecanic acid *tert*-butyl ester (S8)

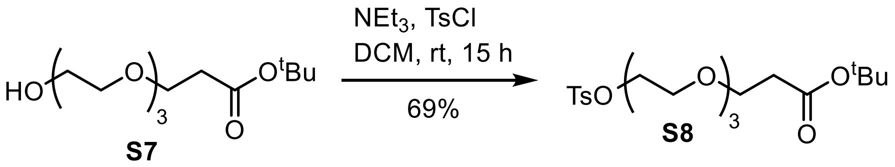

Figure S12: Synthesis of 12-(*p*-toluenesulfonyl)-4,7,10-trioxadodecanic acid *tert*-butyl ester (**S8**).

This compound was synthesized after a modified procedure by *Tavernaro et al.*[7] Alcohol **S7** (1.16 g, 4.16 mmol, 1.00 eq.) was dissolved in anhydrous DCM (5.00 mL) under argon atmosphere. Triethylamine (2.0 mL, 0.01 mol, 2.40 eq.) was added dropwise by syringe. The reaction mixture was cooled with cold water bath and *p*-toluenesulfonyl chloride (1.59 g, 8.32 mmol, 2.00 eq.) was added. The reaction was stirred for 12 h at room temperature. A color change to orange and formation of a colorless precipitate was visible during the reaction. Reaction control by TLC and LC-MS indicated complete conversion of the starting material. The precipitate was filtered off and the organic layer was extracted with water (3x) and brine (3x). The combined aqueous layers were extracted with DCM (2x). The organic layers were dried over Na_2SO_4, filtered and all volatiles were removed in vacuo. The crude product was purified by column chromatography (^{c}Hex/EtOAc, Isolera Flash Purification System). The product was obtained as a light-yellow oil.

Yield: 1.23 g (2.85 mmol, 69 %), (Lit.)[8]: 69 %, light yellow oil.

***R$_f$*:** = 0.26 (^{c}Hex/EtOAc 2:1).

$C_{20}H_{32}O_8S$ (M = 432.18 g/mol).

IR(ATR): $\tilde{\nu}$ (cm^{-1}) = 2978, 2874, 1728, 1365, 1289, 1254, 1177, 1118, 1019, 913, 817, 732, 664, 555.

ESI-MS: m/z (%) = 455.2 (33) $[M+Na]^+$.

^{1}H-NMR, COSY (300 MHz, $CDCl_3$): δ (ppm) = 7.81–7.77 (m, 2H, H-2’), 7.41–7.29 (m, 2H, H-3’),4.21–4.11 (m, 2H, H-9), 3.73–3.57 (m, 12H, H-3, H-4, H-5, H-6, H-7, H-8), 2.49 (t, *J* = 6.6 Hz, 2H, H-2), 2.44 (s, 3H, H-5’), 1.44 (s, 9H, H-11).

^{13}C-NMR, HSQC, HMBC (75 MHz, $CDCl_3$): δ (ppm) = 171.0 (C-1), 144.9 (C-4’), 133.1 (C-1’), 130.0 (C-3’), 128.1 (C-2’), 80.7 (C-10), 70.9 (-*C*H_2-), 70.7 (-*C*H_2-), 70.5 (-*C*H_2-), 69.4 (C-9), 69.3 (-*C*H_2-), 68.8 (C-8), 67.0 (C-3), 36.4 (C-2), 28.2 (C-11), 21.8 (C-5’).

The analytical data are in accordance with the literature.[8]

12-Azido-4,7,10-trioxadodecanic acid tert-butyl ester (S9)

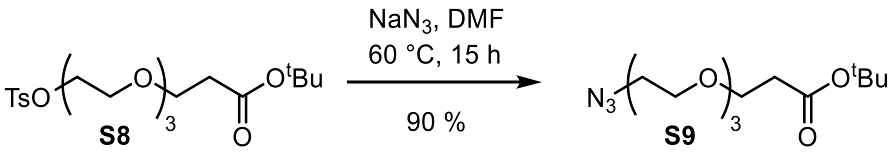

Figure S13: Synthesis of 12-azido-4,7,10-trioxadodecanic acid tert-butyl ester (**S9**).

PEG spacer **S8** (1.13 g, 2.61 mmol, 1.00 eq.) was dissolved in anhydrous DMF (10.0 mL) under argon atmosphere. Sodium azide (0.21 g, 3.27 mmol, 1.30 eq.) was added and the reaction mixture was stirred at 60 °C for 15 h. During the reaction precipitation of a colorless solid was visible. The solid was filtered off and all volatiles were removed in vacuo. The residue was co-distilled with toluene (3x). The residue was dissolved in water and extracted with diethyl ether (3x). The organic layers were combined, dried over Na_2SO_4, filtered and all volatiles were removed in vacuo. The crude product was obtained as a colorless oil.

Yield: 0.72 g (2.37 mmol, 91 %), (Lit.)[7]: 57 % (over 3 steps), colorless oil.

***R$_f$*:** = 0.25 (^{c}Hex/EtOAc 3:1).

$C_{13}H_{25}N_3O_5$ (M = 303.18 g/mol).

IR(ATR): $\tilde{\nu}$ (cm^{-1}) = 2977, 2871, 2105, 1730, 1456, 1367, 1282, 1255, 159, 1120, 908, 848, 731, 649.

ESI-MS: m/z (%) = 304.2 (100) $[M+H]^+$.

^{1}H-NMR, COSY (300 MHz, $CDCl_3$): δ (ppm) = 3.77–3.53 (m, 12H, H-3, H-4, H-5, H-6, H-7, H-8), 3.39 (t, *J* = 5.1 Hz, 2H, H-9), 2.50 (t, *J* = 6.6 Hz, 2H, H-2), 1.44 (s, 9H, H-11).

^{13}C-NMR, HSQC, HMBC (75 MHz, $CDCl_3$): δ (ppm) =170.9 (C-1), 80.5 (C-10), 70.7 (-*C*H_2-), 70.7 (-*C*H_2-), 70.6 (-*C*H_2-), 70.4 (-*C*H_2-), 70.1 (C-8), 66.9 (C-3), 50.7 (C-9), 36.4 (C-2), 28.1 (C-11).

The analytical data are in accordance with the literature.[7]

12-Azido-4,7,10-trioxadodecanic acid (16)

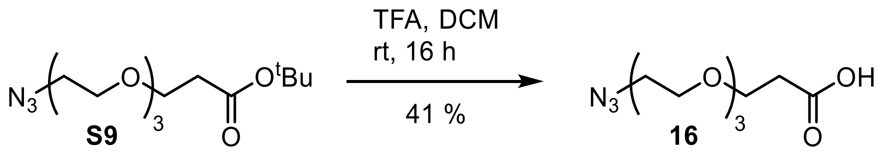

Figure S14: Synthesis of 12-Azido-4,7,10-trioxadodecanic acid (**16**).

PEG spacer **S9** (2.00 g, 6.60 mmol, 1.00 eq.) was dissolved in anhydrous DCM (11.0 mL) under argon atmosphere. TFA (2.00 mL, 26.4 mmol, 4.00 eq.) was added dropwise by syringe. The reaction was stirred for 24 h at room temperature. Reaction control by TLC and LC-MS indicated complete conversion of the starting material. All volatiles were removed in vacuo and the residue was co-distilled with toluene (3x). The crude product was purified by column chromatography (^{c}Hex/EtOAc 1:3 + 1% HCOOH). The product was obtained as a colorless oil.

Yield: 0.67 g (2.71 mmol,41 %), (Lit.)[3] : 94 %, colorless oil.

R$_f$: = 0.29 (^{c}Hex/EtOAc 1:3 + 1 % HCOOH).

$C_9H_{17}N_3O_5$ (M = 247.12 g/mol).

IR(ATR): $\tilde{\nu}$ (cm^{-1}) = 2876, 2106, 1718, 1351, 1288, 1191, 1121, 903, 723, 650.

ESI-MS: m/z (%) = 270.1 (38) $[M+Na]^+$.

HR-MS: m/z = calculated for $[C_9H_{17}N_3O_5+Na]^+$: 270.1065, 270.1053.

^{1}H-NMR, COSY (300 MHz, $CDCl_3$): δ (ppm) = 3.78 (t, *J* = 6.1 Hz, 2H, H-3), 3.73–3.49 (m, 10H, H-4, H-5, H-6, H-7, H-8), 3.40 (t, *J* = 5.6 Hz, 2H, H-9), 2.65 (t, *J* = 6.1 Hz, 2H, H-2).

^{13}C-NMR, HSQC, HMBC (75 MHz, $CDCl_3$): δ (ppm) = 174.9 (C-1), 70.6 (-*C*H$_2$-), 70.6 (-*C*H$_2$-), 70.4 (-*C*H$_2$-), 70.3 (-*C*H$_2$-), 70.0 (C-8), 66.3 (C-3), 50.7 (C-9), 34.7 (C-2).

The analytical data is in accordance with the literature.[9]

II.3 Synthesis of the clickable mannose

Clickable mannose **1** was synthesized as previously described (see Figure S15).[10]

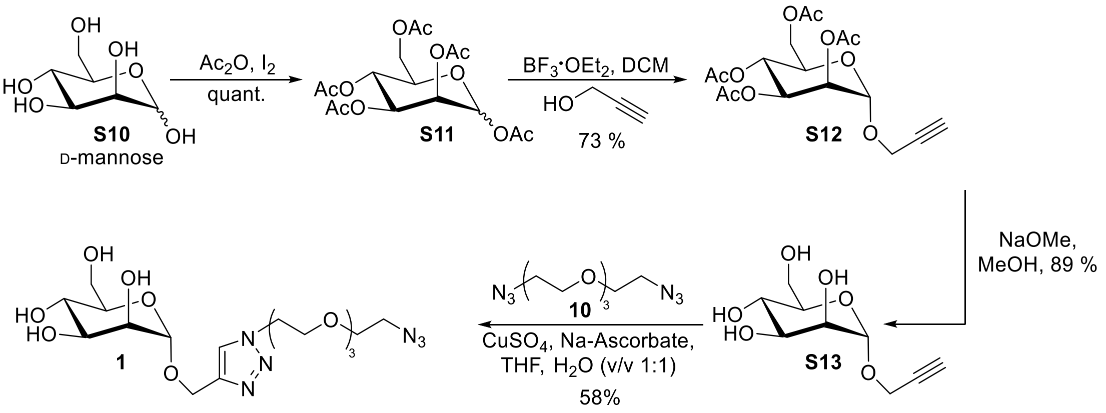

Figure S15: Synthesis of (1-(2-(2-(2-(2-azidoethoxy)ethoxy)ethoxy)ethyl)-1*H*-1,2,3-triazol-4-yl)methoxy-α-D-mannopyranoside (**1**).

II.4 Synthesis of the clickable fucose-*N*-acetyl-glucosamine-disaccharide

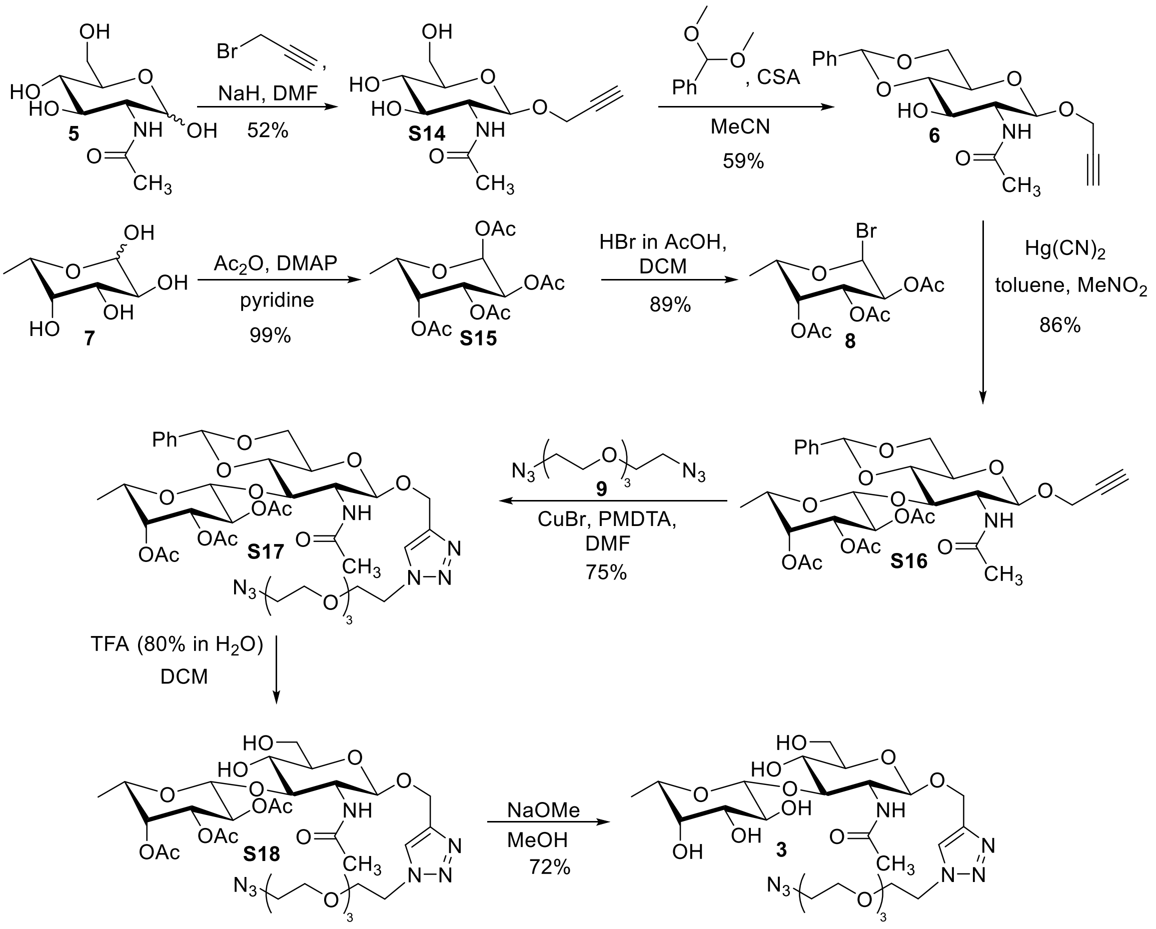

Figure S16: Synthesis of (1-(2-(2-(2-(2-azidoethoxy)ethoxy)ethoxy)ethyl)-1*H*-1,2,3-triazol-4-yl)methyl-(β-L-fucopyranosyl)-(1→3)-2-acetamido-2-deoxy-β-D-glucopyranoside (**3**).

Propargyl 2-acetamido-2-deoxy-β-D-glucopyranoside (S14)

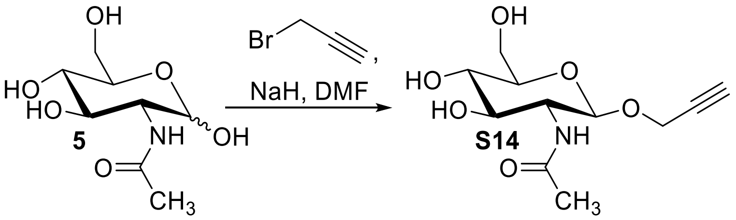

Figure S17: Synthesis of propargyl 2-acetamido-2-deoxy-β-D-glucopyranoside (**S14**).

The synthesis was performed after a modified procedure by *Beau et al.*[11]

A solution of *N*-acetylglucosamine (**5**, 3.00 g, 13.6 mmol, 1.0 eq.) was dissolved in DMF (14 mL) under argon atmosphere. NaH (60% dispersion on mineral oil, 700 mg, 17.7 mmol, 1.3 eq.) was added. Afterwards propargyl bromide (80% in toluene, 3.88 mL, 40.8 mmol, 2.3 eq.) was added at room temperature. The reaction was stirred for 4 h at room temperature and afterwards water (2.0 mL) was added. All volatiles were removed under reduced pressure and the residue was purified by column chromatography (DCM/MeOH 4:1). The product was obtained as a yellow oil.

Yield: 1.83 g (7.04 mmol, 52%), yellow oil.

$\boldsymbol{R_f}$ = 0.35 (DCM/MeOH 4:1).

$C_{11}H_{17}NO_6$ (259.26 g/mol).

IR (ATR) $\tilde{\nu}$ (cm^{-1}) = 3378, 3320, 3286, 3258, 2933, 2872, 1741, 1667, 1620, 1548, 1528, 1376, 1312, 1276, 1093, 1057, 1018, 912, 682, 646, 619.

$[\boldsymbol{\alpha}]_{\boldsymbol{D}}^{\boldsymbol{23}}$ = –39.3° (c = 1.00, Methanol).

ESI-MS: *m/z* (%) = 282.2 (100) $[M + Na]^+$, 283.1 (5) $[M(^{13}C_1) + Na]^+$.

^{1}H-NMR, COSY (400 MHz, CD_3OD) δ (ppm) = 4.56 (d, ^3J = 8.4 Hz, 1H, H-1), 4.33 (*pseudo* dd, ^4J = 2.5 Hz, J = 1.4 Hz, 2H, $-CH_2-C{\equiv}C-$), 3.85 (dd, ^2J = 12.0 Hz, ^3J = 2.0 Hz, 1H, H-6_a), 3.69–3.60 (m, 2H, H-2, H-6_b), 3.45 (dd, ^3J = 10.3 Hz, ^3J = 8.2 Hz, 1H, H-3), 3.29–3.24 (m, 2H, H-4, H-5), 2.82 (t, ^3J = 2.4 Hz, 1H, C≡C*H*), 1.95 (s, 3H, $COCH_3$).

^{13}C-NMR, HSQC, HMBC (100.6 MHz, CD_3OD) δ (ppm) = 172.50 (C=O), 99.1 (C-1, $^1J_{C,H}$ = 157 Hz), 78.6 (*C*≡CH), 76.6 (C-5), 74.8 (C≡*C*H), 74.5 (C-3), 70.6 (C-4), 61.3 (C-6), 55.6 (C-2), 55.1 (CH_2–C≡C), 21.6 ($COCH_3$).

The analytical data is accordance with the literature.[12]

Propargyl 2-acetamido-2-deoxy-4,6-*O*-benzylidene-β-D-glucopyranoside (6)

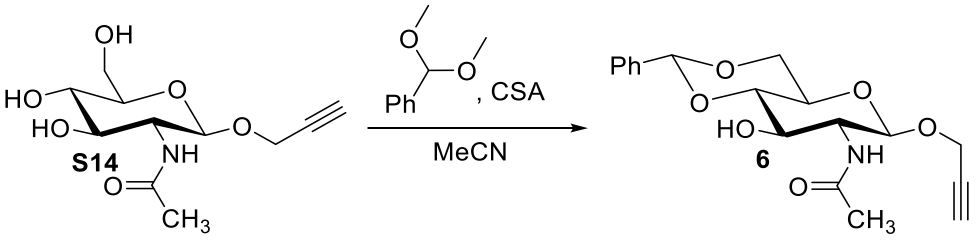

Figure S18: Synthesis of propargyl 2-acetamido-2-deoxy-4,6-*O*-benzylidene-β-D-glucopyranoside (**6**).

The synthesis was performed after a modified procedure by *McGuigan et al.*[13]

To a suspension of propargyl 2-acetamido-2-deoxy-β-D-glucopyranoside (**S14**, 1.00 g, 3.86 mmol, 1.00 eq.) and camphor-10-sulfonic acid (63 mg, 0.27 mmol, 7 mol%) in acetonitrile (11.0 mL) under argon atmosphere was added benzaldehyde dimethyl acetal (2.04 mL, 13.6 mmol, 3.5 eq.). The reaction was stirred for 2 h at room temperature. Triethylamine (100 μL) was added and all volatiles were removed in vacuo. The residue was dissolved in ethanol (90%$_{aq}$) under reflux and slowly allowed to cool to room temperature. The resulting precipitate was filtered off and washed with cold ethanol (2x). The product was obtained as a colorless solid.

Yield: 788 mg (2.27 mmol, 59%), colorless solid.

$C_{18}H_{21}NO_6$ (347.37 g/mol).

R$_f$ = 0.40 (EtOAc).

IR (ATR) $\tilde{\nu}$ (cm^{-1}) = 3280, 2877, 1656, 1556, 1374, 1315, 1085, 1046, 1012, 748, 698.

$[\alpha]_D^{23}$ = –86.5° (c = 0.26, Methanol).

ESI-MS: *m/z* (%) = 370.5 (100) [M + Na]$^+$, 349.3 (45) [M + H]$^+$, 293.0 (10) [M–O–CH_2–C≡CH]$^+$.

^{1}H-NMR, COSY (400 MHz, DMSO-*d$_6$*) *δ* (ppm) = 7.85 (d, ^3J = 8.9 Hz, 1H, NH), 7.49–7.41 (m, 2H, H-2–Ar, H-6–Ar), 7.41–7.35 (m, 3H, H-4–Ar, H-3–Ar, H-5–Ar), 5.60 (s, 1H, Ph–C*H*–O), 5.32 (s, 1H, *H*O–C-3), 4.61 (d, ^3J = 8.3 Hz, 1H, H-1), 4.34 (dd, ^3J = 15.9 Hz, ^4J = 2.5 Hz, 1H, CH_{2a}–C≡C), 4.26–4.17 (m, 2H, H-6$_a$, CH_{2b}–C≡C), 3.78–3.63 (m, 2H, H-3, H-6$_b$), 3.57–3.40 (m, 3H, H-2, H-4, C≡C*H*), 3.34–3.29 (m, 1H, H-5), 1.82 (s, 3H, COCH_3).

^{13}C-NMR, HSQC, HMBC (100.6 MHz, DMSO-*d$_6$*) *δ* (ppm) = 169.2 (*C*OCH$_3$), 137.7 (*C*–CH–O), 128.9 (C-4–Ar), 128.0 (C-3–Ar, C-5–Ar), 126.4 (C-2–Ar, C-6–Ar), 100.7 (Ph–*C*H–O), 99.9 (C-1), 81.1 (C-4), 79.6 (*C*≡CH), 77.6 (C≡*C*H), 70.2 (C-3), 67.8 (C-6), 66.0 (C-5), 56.0 (C-2), 55.5 (*C*H$_2$–C≡C), 23.1 (CO*C*H$_3$).

The analytical data is in accordance with the literature.[13]

1,2,3,4-Tetra-*O*-acetyl-α-L-fucopyranoside (S15)

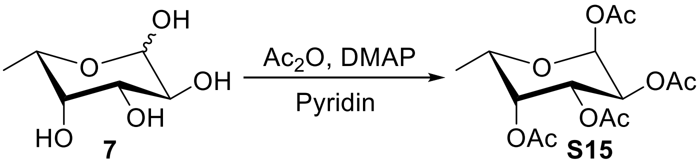

Figure S19: Synthesis of 1,2,3,4-tetra-*O*-acetyl-α-L-fucopyranoside (**S15**).

This compound was synthesized after a modified procedure by *Singh et al.*[14]

A solution of L-fucose (**7**, 1.31 g, 8.0 mmol, 1.00 eq.), DMAP (cat., 40 mg) and acetic anhydride (19.7 mL, 209.0 mmol, 24.00 eq.) in pyridine (20.0 mL) under argon atmosphere was stirred for 16 h at room temperature. The reaction mixture was diluted with EtOAc (100 mL) and extracted with 1N HCl (4x), sat. $NaHCO_3$ solution (2x) and brine (1x). The organic layers were combined and dried over Na_2SO_4, filtered and all volatiles were removed in vacuo. The product was obtained as a yellow oil.

Yield: 2.63 g (7.91 mmol, 99%), yellow oil.

$C_{14}H_{20}O_9$ (332.31 g/mol).

$\boldsymbol{R_f}$ = 0.23 (^{c}Hex/EtOAc 3:1).

IR (ATR) $\tilde{\nu}$ (cm^{-1}) = 2988, 1746, 1434, 1371, 1252, 1217, 1138, 1074, 1045, 1013, 971, 935, 819, 749.

$[\boldsymbol{\alpha}]_{\boldsymbol{D}}^{\boldsymbol{23}}$ = –100.5° (c = 1.00, $CHCl_3$).

ESI-MS: *m/z* (%) = 355.5 (100) $[M + Na]^+$, 356.3 (4) $[M(^{13}C_1) + Na]^+$, 273.5 (23) $[M–OAc]^+$.

^{1}H-NMR, COSY (400 MHz, $CDCl_3$) *δ* (ppm) = 6.34 (d, ^3J = 3.1 Hz, 1H, H-1), 5.35–5.30 (m, 3H, H-2, H-3, H-4), 4.27 (q, ^3J = 6.5 Hz 1H, H-5), 2.17, 2.14, 2.01, 1.99 (4 × s, je 3H, 4 × $COCH_3$), 1.15 (d, ^3J = 6.5 Hz, 3H, H-6)

^{13}C-NMR, HSQC, HMBC (100.6 MHz, $CDCl_3$) *δ* (ppm) = 170.7, 170.3, 170.1, 169.3 (4 × $COCH_3$), 90.1 (C-1, $^1J_{C,H}$ = 178 Hz), 70.7 (C-2), 68.0 (C-3), 67.4 (C-4), 66.6 (C-5), 21.1, 20.8, 20.8, 20.7 (4 × $COCH_3$), 16.1 (C-6).

The analytical data are in accordance with the literature.[14]

1,2,3-Tri-*O*-acetyl-α-L-fucopyranosyl bromide (8)

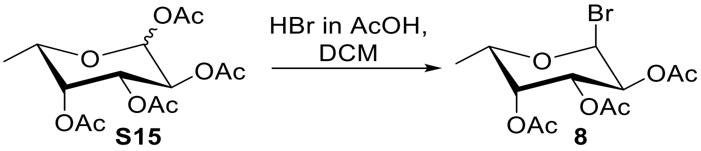

Figure S20: Synthesis of 1,2,3-tri-*O*-acetyl-α-L-fucopyranosyl bromide (**8**).

This compound was synthesized after a modified by procedure by *Singh et al.*[14]

A solution of protected fucose **S15** (1.15 g, 3.45 mmol, 1.00 eq.) in DCM (5.0 mL) was cooled to 0 °C. HBr (40% in acetic acid, 4.58 mL, 26.5 mmol, 7.7 eq.) was added dropwise. The reaction was stirred for 4 h at 0 °C. After complete consumption of the starting material, DCM was added. The organic layer was extracted with water, sat. $NaHCO_3$ solution (4x) and brine. The organic layer was dried over Na_2SO_4, filtered and all volatiles were removed in vacuo. The product was obtained as a yellow oil.

Yield: 1.08 g (3.07 mmol, 89%), yellow oil.

$C_{12}H_{17}BrO_7$ (353.17 g/mol).

$\boldsymbol{R_f}$ = 0.44 (cyclohexane/ethyl acetate 1:1).

IR (ATR) $\tilde{\nu}$ (cm^{-1}) = 2988, 1744, 1432, 1371, 1239, 1218, 1165, 1132, 1104, 1077, 1020, 914, 876, 825.

$[\boldsymbol{a}]_{\boldsymbol{D}}^{\boldsymbol{23}}$ = –212.8° (c = 2.00, $CHCl_3$).

ESI-MS: *m/z* (%) = 313.4 (100) [M–Br + OH + Na]$^+$, 314.3 (7) [M($^{13}C_1$)–Br + OH + Na]$^+$, 274.0 (18) [M–Br]$^+$.

^{1}H-NMR, COSY (400 MHz, $CDCl_3$) δ (ppm) = 6.69 (d, ^3J = 4.1 Hz, 1H, H-1), 5.41 (dd, ^3J = 10.6 Hz, ^3J = 3.3 Hz, 1H, H-3), 5.36 (dd, ^3J = 3.3 Hz, ^3J = 1.2 Hz, 1H, H-4), 5.02 (dd, ^3J = 10.6 Hz, ^3J = 3.9 Hz, 1H, H-2), 4.44 – 4.36 (m, 1H, H-5), 2.17, 2.11, 2.01 (3 × s, je 3H, 3 × COC*H*$_3$), 1.21 (d, ^3J = 6.4 Hz, 3H, C*H*$_3$).

^{13}C-NMR, HSQC, HMBC (100.6 MHz, $CDCl_3$) δ (ppm) = 170.4, 170.3, 170.0 (3 × *C*OCH$_3$), 89.4 (C-1, $^1J_{C,H}$ = 189 Hz), 70.1 (C-4), 70.0 (C-5), 68.6 (C-3), 68.0 (C-2), 20.9, 20.8, 20.7 (3 × CO*C*H$_3$), 15.7 (C-6).

The analytical data is in accordance with the literature.[14]

Propargyl (2,3,4-tri-*O*-acetyl-β-L-fucopyranosyl)-(1→3)-2-acetamido-2-deoxy-4,6-*O*-benzylidene-β-D-glucopyranoside (S16)

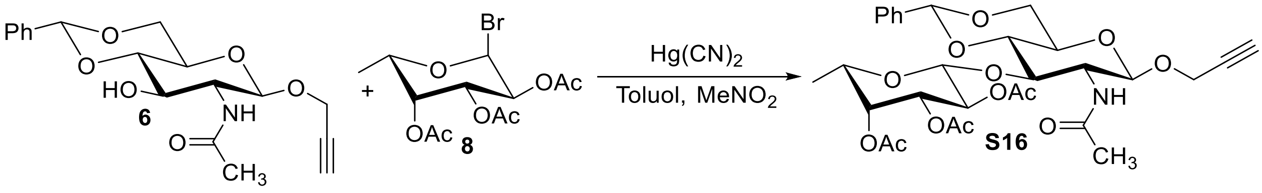

Figure S21: Synthesis of propargyl (2,3,4-tri-*O*-acetyl-β-L-fucopyranosyl)-(1→3)-2-acetamido-2-deoxy-4,6-*O*-benzylidene-β-D-glucopyranoside (**S16**).

The synthesis was performed after a modified procedure by *Hindsgaul et al.*[15]

A suspension of glycosyl donor **8** (2.43 g, 6.91 mmol, 1.6 eq.) and glycosyl acceptor **6** (1.50 g, 4.32 mmol, 1.0 eq.) in toluene (62 mL) and nitromethane (47 mL) with molecular sieves (4 Å) was stirred at room temperature for 15 min. $Hg(CN)_2$ (1.31 g, 5.18 mmol, 1.2 eq.) was added and the reaction mixture was heated to 70 °C. After completion of the reaction, the mixture was allowed to cool to room temperature. The mixture was filtered over celite and the organic layer was extracted with water (2x), 2M KI solution (3x) and brine (2x), dried over Na_2SO_4, filtered and all volatiles were removed in vacuo. The crude product was purified by column chromatography (^{c}Hex/EtOAc, 1:1). The product was isolated as a yellow, amorphous solid.

Yield: 2.29 g (3.69 mmol, 86%), yellow, amorphous solid.

$C_{30}H_{37}NO_{13}$ (619.62 g/mol).

$\boldsymbol{R_f}$ = 0.15 (^{c}Hex/EtOAc 1:1).

IR (ATR) $\tilde{\nu}$ (cm^{-1}) = 3288, 2983, 2871, 1746, 1661, 1557, 1369, 1181, 1035, 1013, 912, 731, 698.

$[\alpha]_D^{22}$ = –29.4° (c 1.00, $CHCl_3$).

ESI-MS: *m/z* (%) = 642.3 (100) [M + Na]$^+$, 623.2 (30) [M + Na]$^+$, 620.2 (17) [M + H]$^+$, 564.3 (16) [M–O–CH_2–C≡CH]$^+$, 348.3 (26) [M–Fuc + H]$^+$.

HRMS (ESI): Calculated for [$C_{30}H_{37}NO_{13}$ + Na]$^+$: 642.2163, found: 642.2180.

^{1}H-NMR, COSY (400 MHz,$CDCl_3$) δ (ppm) = 7.51–7.46 (m, 2H, H–Ar), 7.43–7.36 (m, 3H, H–Ar), 5.90–5.84 (m, 1H, N*H*), 5.48 (s, 1H, Ph–C*H*–O), 5.35 (d, ^3J = 8.2 Hz, 1H, H_{GlcNAc}-1), 5.20 (dd, ^3J = 3.5 Hz, ^3J = 1.1 Hz, 1H, H_{Fuc}-4), 5.13 (dd, ^3J = 10.4 Hz, ^3J = 8.0 Hz, 1H, H_{Fuc}-2), 4.97 (dd, ^3J = 10.4 Hz, ^3J = 3.5 Hz, 1H, H_{Fuc}-3), 4.67 (d, ^3J = 8.0 Hz, 1H, H_{Fuc}-1), 4.59 (dd, ^3J = 9.9 Hz, ^3J = 8.5 Hz, 1H, H_{GlcNAc}-3), 4.39 (dd, ^2J = 15.8 Hz, ^4J = 2.4 Hz, 1H, CH_{2a}–C≡C), 4.37–4.33 (m, 1H, H_{GlcNAc}-6$_a$), 4.33 (dd, ^2J = 15.7 Hz, ^4J = 2.4 Hz, 1H, CH_{2b}–C≡C), 3.79–3.71 (m, 2H, H_{Fuc}-5, H_{GlcNAc}-6$_b$), 3.58–3.48 (m, 2H, H_{GlcNAc}-4, H_{GlcNAc}-5), 3.11 (ddd, ^3J = 9.9 Hz, ^3J = 8.2 Hz, ^3J = 6.8 Hz, 1H, H_{GlcNAc}-2), 2.46 (t, ^4J = 2.4 Hz, 1H, C≡C*H*), 2.16, 1.98, 1.96, 1.81 (4 × s, je 3H, 4 × COCH_3), 1.18 (d, ^3J = 6.4 Hz, 3H, 3 × H_{Fuc}-6).

^{13}C-NMR, HSQC, HMBC (100.6 MHz, $CDCl_3$) δ (ppm) = 171.1 (NH*C*OCH_3), 170.7, 170.3, 169.8 (3 × *C*OCH_3), 137.2 (C_q–Ar), 129.3 (C-4–Ar), 128.5 (C-3–Ar, C-5–Ar), 126.0 (C-2–Ar, C-6–Ar), 101.9 (Ph–*C*H–O), 101.4 (C_{Fuc}-1, $^1J_{C,H}$ = 161 Hz), 98.5 (C_{GlcNAc}-1, $^1J_{C,H}$ = 165 Hz), 81.8 (C_{GlcNAc}-4), 78.8 (*C*≡CH), 76.0 (C_{GlcNAc}-3), 75.3 (C≡*C*H), 71.4 (C_{Fuc}-3), 70.3 (C_{Fuc}-4), 69.2 (C_{Fuc}-5), 69.1 (C_{Fuc}-2), 68.8 (C_{GlcNAc}-6), 65.8 (C_{GlcNAc}-5), 57.8 (C_{GlcNAc}-2), 56.5 (*C*H_2–C≡CH), 23.9, 20.8, 20.8, 20.7 (4 × CO*C*H_3), 16.3 (C_{Fuc}-6).

(1-(2-(2-(2-(2-Azidoethoxy)ethoxy)ethoxy)ethyl)-1*H*-1,2,3-triazol-4-yl)methyl (2,3,4-tri-*O*-acetyl-β-L-fucopyranosyl)-(1→3)-2-acetamido-2-deoxy-4,6-*O*-benzylidene-β-D-glucopyranoside (S17)

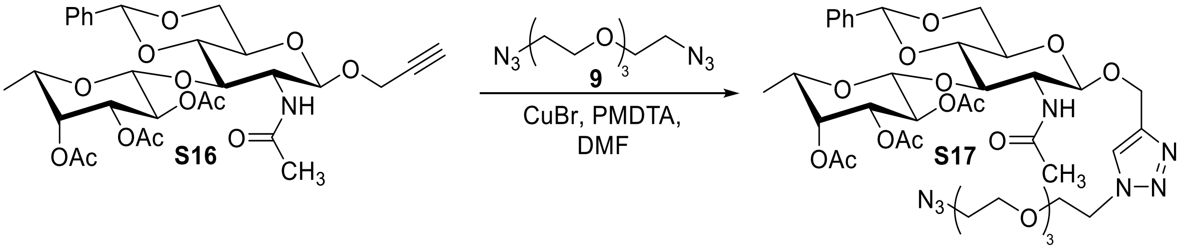

Figure S22: Synthesis of (1-(2-(2-(2-(2-azidoethoxy)ethoxy)ethoxy)ethyl)-1*H*-1,2,3-triazol-4-yl)methyl (2,3,4-tri-*O*-acetyl-β-L-fucopyranosyl)-(1→3)-2-acetamido-2-deoxy-4,6-*O*-benzyliden-β-D-glucopyranoside (**S17**).

This compound was synthesized after a modified procedure by *Kazuo et al.*[16]

A solution of disaccharide **S16** (500 mg, 0.81 mmol, 1.0 eq.), PEG spacer **9** (1.97 g, 8.03 mmol, 9.9 eq.), PMDETA (50 μL, 0.24 mmol, 30 mol%) in DMF (20.0 mL) was degassed using the freeze-pump thaw method (3x). The solution was heated under argon to 45 °C and CuBr (14.0 mg, 0.1 mmol, 12 mol%) was added. The reaction mixture was stirred for 1.5 h at 45 °C. The mixture was afterwards diluted with EtOAc (40 mL) and extracted with sat. NH_4Cl solution (2x). The aqueous phase was extracted with EtOAc. The organic layers were combined, dried over Na_2SO_4, filtered and all volatiles were removed in vacuo. The crude product was purified by column chromatography (DCM/MeOH, Isolera Flash Purification System). The product was obtained as a yellow oil.

Yield: 523 mg (0.60 mmol, 75%), yellow oil.

$C_{38}H_{53}N_7O_{16}$ (863.88 g/mol).

R_f = 0.28 (DCM/MeOH 9:1).

IR (ATR) $\tilde{\nu}$ (cm^{-1}) = 3012, 2938, 2873, 2105, 1747, 1666, 1549, 1369, 1179, 1070, 747, 700, 666.

$[\alpha]_D^{22}$ = –25.9° (c 1.00, $CHCl_3$).

ESI-MS: *m/z* (%) = 592.6 (100) [M–Fuc + H]$^+$, 565.4 (13) [M–O–CH_2–triazole–(CH_2–O)$_3$–CH_2–CH_2–N_3]$^+$, 864.4 (3) [M + H]$^+$, 886.3 (2) [M + Na]$^+$.

HRMS (ESI): Calculated for [$C_{38}H_{53}N_7O_{16}$ + Na]$^+$: 886.3446, found: 886.3441.

^{1}H-NMR, COSY (400 MHz,$CDCl_3$) δ (ppm) = 7.73 (s, 1H, $H_{triazol}$), 7.52–7.45 (m, 2H, H–Ar), 7.43–7.35 (m, 3H, H–Ar), 5.94 (d, ^3J = 7.0 Hz, 1H, N*H*), 5.49 (s, 1H, Ph–C*H*–O), 5.23 (d, ^3J = 8.2 Hz, 1H, H_{GlcNAc}-1), 5.19 (d, ^3J = 2.9 Hz, 1H, H_{Fuc}-4), 5.11 (dd, ^3J = 10.5 Hz, ^3J = 7.9 Hz, 1H, H_{Fuc}-2), 5.01–4.91 (m, 2H, O–C*H*$_{2a}$–C=C, H_{Fuc}-3), 4.77 (d, ^2J = 12.4 Hz, 1H, O–C*H*$_{2b}$–C=C), 4.66 (d, ^3J = 8.0 Hz,

1H, H_{Fuc}-1), 4.57–4.50 (m, 2H, triazole–CH_2), 4.47 (*pseudo* t, ^3J = 8.9 Hz, 1H, H_{GluNAc}-3), 4.38 (dd, ^2J = 10.8 Hz, ^3J = 3.9 Hz, 1H, H_{GlcNAc}-6$_a$), 3.87 (t, ^3J = 5.1 Hz, 2H, triazole–CH_2–CH_2), 3.81–3.70 (m, 2H, H_{GlcNAc}-6$_b$, H_{Fuc}-5), 3.71–3.60 (m, 10H, Alkyl–H), 3.57–3.49 (m, 2H, H_{GlcNAc}-5, H_{GlcNAc}-4), 3.44–3.36 (m, 2H, N_3–CH_2), 3.19 (q, ^3J = 8.2 Hz, 1H, H_{GlcNAc}-2), 2.15, 1.96, 1.91, 1.81 (4 × s, je 3H, 4 × COCH_3), 1.17 (d, ^3J = 6.4 Hz, 3H, 3 × H_{Fuc}-6).

^{13}C-NMR, HSQC, HMBC (100.6 MHz, $CDCl_3$) δ (ppm) = 171.0, 170.7, 170.3, 169.7 (4 × *C*OCH_3), 144.3 (C_q–triazole), 137.2 (C_q–Ar), 129.3 (C-4–Ar), 128.5 (C-3–Ar, C-5–Ar), 126.0 (C-2–Ar, C-6–Ar), 124.2 (*C*H–triazole), 101.8 (C_{Fuc}-1), 101.4 (Ph–*C*H–O), 99.9 (C_{GlcNAc}-1), 81.8 (C_{GlcNAc}-4), 76.3 (C_{GlcNAc}-3), 71.5 (C_{Fuc}-3), 70.9 (Alkyl *C*H_2), 70.7, 70.7, 70.7 (3x Alkyl *C*H_2), 70.3 (Alkyl *C*H_2), 70.3 (C_{Fuc}-4), 69.5 (triazole–CH_2–*C*H_2), 69.2 (C_{Fuc}-5), 69.1 (C_{Fuc}-2), 68.9 (C_{GlcNAc}-6), 65.9 (C_{GlcNAc}-5), 63.0 (*C*H_2–C=CH), 57.7 (C_{GlcNAc}-2), 50.8 (N_3–*C*H_2), 50.4 (triazole–*C*H_2), 23.8, 20.8, 20.8, 20.7 (4 × CO*C*H_3), 16.3 (C_{Fuc}-6).

(1-(2-(2-(2-(2-Azidoethoxy)ethoxy)ethoxy)ethyl)-1*H*-1,2,3-triazol-4-yl)methyl (2,3,4-tri-*O*-acetyl-β-L-fucopyranosyl)-(1→3)-2-acetamido-2-deoxy-β-D-glucopyranoside (S18)

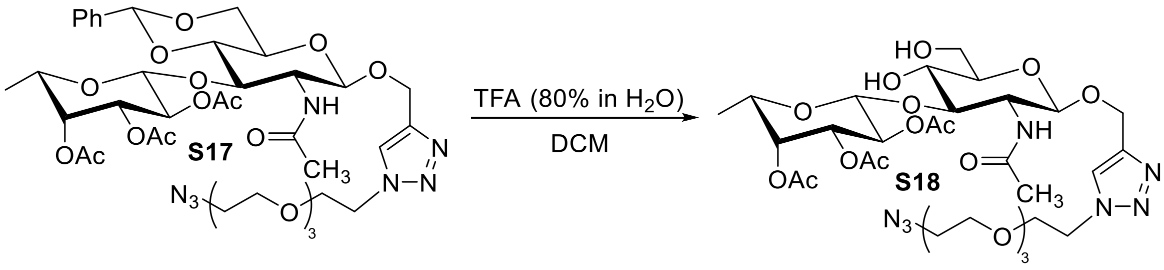

Figure S23: Synthesis of (1-(2-(2-(2-(2-azidoethoxy)ethoxy)ethoxy)ethyl)-1*H*-1,2,3-triazol-4-yl)methyl (2,3,4-tri-*O*-acetyl-β-L-fucopyranosyl)-(1→3)-2-acetamido-2-deoxy-β-D-glucopyranoside (**S18**).

This compound was synthesized after a modified procedure by *Chaikof et al.*[17]

A solution of disaccharide **S17** (400 mg, 0.52 mmol, 1.00 eq.) in DCM (10.0 mL) was treated with a mixture of trifluoroacetic acid (2.1 mL, 27.0 mmol, 52.00 eq.) and water (0.9 mL). The reaction was stirred for 2 h at room temperature. Afterwards, all volatiles were removed in vacuo. The residue was co-distilled with toluene (3x). The crude product was obtained as a colorless oil and was used without further purification. An analytical sample (40 mg, 0.05 mmol) was purified by column chromatography (DCM/MeOH 95:5), which yielded the product as a colorless oil.

Yield: 31 mg (0.04 mmol, 77%), colorless oil.

$C_{31}H_{49}N_7O_{16}$ (775.77 g/mol).

R$_f$ = 0.12 (DCM/MeOH 95:5).

IR (ATR) $\tilde{\nu}$ (cm^{-1}) = 3319, 2921, 2870, 2105, 1744, 1657, 1554, 1369, 1222, 1058, 932, 908.

$[\alpha]_D^{22}$ = –6.9° (c 1.00, $CHCl_3$).

ESI-MS: *m/z* (%) = 504.8 (100) [M –Fuc + H]$^+$, 505.6 (37) [M($^{13}C_1$)–Fuc + H]$^+$, 477.8 (63) [M–O–CH_2–triazol–(CH_2–O)$_3$–CH_2–CH_2–N_3]$^+$, 478.7 (24) [M($^{13}C_1$)–O–CH_2–triazol–(CH_2–O)$_3$–CH_2–CH_2–N_3], 798.3 (3) [M + Na]$^+$, 776.2 (2) [M + H]$^+$.

HRMS (ESI): Calculated for [$C_{31}H_{49}N_7O_{16}$ + Na]$^+$: 798.3133, found: 798.3115.

^{1}H-NMR, COSY (400 MHz,$CDCl_3$) δ (ppm) = 7.77 (s, 1H, $H_{triazole}$), 6.21 (d, ^3J = 7.1 Hz, 1H, N*H*), 5.19 (d, ^3J = 3.5 Hz, 1H, H_{Fuc}-4), 5.15–5.06 (m, 2H, H_{Fuc}-2, H_{GlcNAc}-1), 4.99 (dd, ^3J = 10.4 Hz, ^3J = 3.4 Hz, 1H, H_{Fuc}-3), 4.93 (d, ^2J = 12.4 Hz, 1H, O–CH_{2a}–C=C), 4.79–4.71 (m, 2H, H_{Fuc}-1, O–CH_{2b}–C=C), 4.53 (t, ^3J = 5.0 Hz, 2H, triazole–CH_2), 4.17 (*pseudo* t, ^3J = 9.6 Hz, 1H, H_{GlcNAc}-3), 3.91–3.75 (m, 5H, H_{GlcNAc}-6$_{a,b}$, H_{Fuc}-5, triazole–CH_2–CH_2), 3.70–3.56 (m, 11H, H_{GlcNAc}-4, 10x Alkyl–H), 3.46–3.41 (m, 1H, H_{GlcNAc}-5), 3.39 (t, ^3J = 5.0 Hz, 2H, N_3–CH_2), 3.22–3.13 (m, 1H, H_{GlcNAc}-2), 2.16, 1.96, 1.95, 1.92 (4 × s, je 3H, 4 × COCH_3), 1.18 (d, ^3J = 6.3 Hz, 3H, 3 × H_{Fuc}-6).

^{13}C-NMR, HSQC, HMBC (100.6 MHz, $CDCl_3$) δ (ppm) = 171.2, 170.7, 170.3, 170.2 (4 × *C*OCH_3), 144.3 (C_q–triazole), 124.5 (*C*H–triazole), 101.8 (C_{Fuc}-1), 99.9 (C_{GlcNAc}-1), 80.5 (C_{GlcNAc}-3), 75.3 (C_{GlcNAc}-5), 71.8 (C_{GlcNAc}-4), 71.4 (C_{Fuc}-3), 70.8 (Alkyl *C*H_2), 70.7, 70.6 (2x Alkyl *C*H_2), 70.3 (C_{Fuc}-4), 70.2 (Alkyl *C*H_2), 69.6 (triazole–CH_2–*C*H_2), 69.5 (C_{Fuc}-2), 69.2 (C_{Fuc}-5), 62.5 (C_{GlcNAc}-6), 62.5 (*C*H_2–C=CH), 56.7 (C_{GlcNAc}-2), 50.8 (N_3–*C*H_2), 50.4 (triazole–*C*H_2), 23.8, 20.9, 20.8, 20.7 (4 × CO*C*H_3), 16.3 (CH_3).

(1-(2-(2-(2-(2-Azidoethoxy)ethoxy)ethoxy)ethyl)-1*H*-1,2,3-triazol-4-yl)methyl (β-L-fucopyranosyl)-(1→3)-2-acetamido-2-deoxy-β-D-glucopyranoside (3)

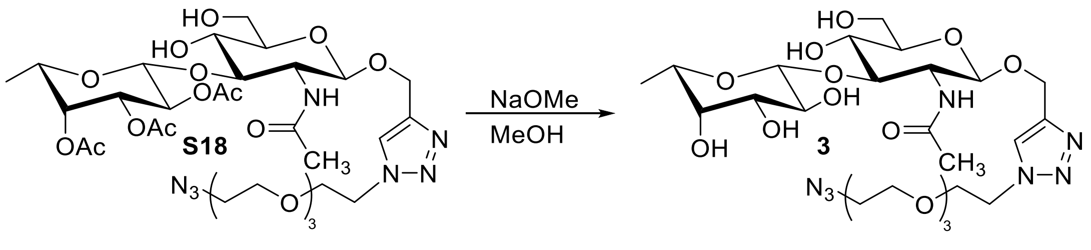

Figure S24: Synthesis of (1-(2-(2-(2-(2-azidoethoxy)ethoxy)ethoxy)ethyl)-1*H*-1,2,3-triazol-4-yl)methyl (β-L-fucopyranosyl)-(1→3)-2-acetamido-2-deoxy-β-D-glucopyranoside (**3**).

This compound was synthesized after a modified procedure by *Zemplén et al.*[18]

A solution of disaccharide **S18** (90 mg, 0.12 mmol) in MeOH (4.0 mL) was treated with NaOMe until a pH of 9–10 was reached (10 mg). The reaction mixture was stirred for 17 h at room temperature and afterwards neutralized with Amberlite IR 120 H^+-resin. The reaction mixture

was afterwards filtered over celite and all volatiles were removed in vacuo. The crude product was purified by preparative HPLC (MeCN/H_2O, gradient from 10-90% MeCN in 30 min, R_t=6.06 min). The product was obtained as a colorless amorph solid after freeze drying.

Yield: 56 mg (0.08 mmol, 72%), colorless amorph solid.

$C_{25}H_{43}N_7O_{13}$ (649.66 g/mol).

R_f = 0.24 (RP-Silica gel, MeCN/H_2O 4:1), 0.34 (DCM/MeOH/Et_3N 5:1.5:0.1)

IR (ATR) $\tilde{\nu}$ (cm^{-1}) = 3274, 2932, 2872, 2107, 1652, 1564, 1459, 1373, 1308, 1160, 1071, 941.

$[\alpha]_D^{22}$ = –28.8° (c 1.00, MeOH).

ESI-MS: *m/z* (%) = 650.8 (100) $[M + H]^+$, 651.8 (23) $[M(^{13}C_1) + H]^+$, 672.4 (80) $[M + Na]^+$, 673.3 (6) $[M(^{13}C_1) + Na]^+$.

HRMS (ESI): Calculated for $[C_{25}H_{43}N_7O_{13} + Na]^+$: 672.2817, found: 672.2828.

^{1}H-NMR, COSY (600 MHz, CD_3OD) δ (ppm) = 8.01 (s, 1H, $H_{triazole}$), 4.93–4.91 (m, 1H, O–CH_{2a}–C=C), 4.74 (d, ^2J = 12.4 Hz, 1H, O–CH_{2b}–C=C), 4.63 (d, ^3J = 8.3 Hz, 1H, H_{GlcNAc}-1), 4.60–4.55 (m, 2H, triazole–CH_2), 4.39 (d, ^3J = 7.6 Hz, 1H, H_{Fuc}-1), 3.93–3.87 (m, 3H, H_{GlcNAc}-6_a, triazole–CH_2–CH_2), 3.73–3.41 (m, 19H, H_{GlcNAc}-2, N*H*, H_{GlcNAc}-3, H_{GlcNAc}-4, H_{GlcNAc}-6_b, H_{Fuc}-2, H_{Fuc}-3, H_{Fuc}-4, H_{Fuc}-5, 10x Alkyl–H), 3.38–3.32 (m, 3H, N_3–CH_2, H_{GlcNAc}-5), 1.92 (s, 3H, COCH_3), 1.26 (d, ^3J = 6.4 Hz, 3H, CH_3).

^{13}C-NMR, HSQC, HMBC (150.9 MHz, CD_3OD) δ (ppm) = 173.8 (*C*OCH_3), 145.3 (C_q–Triazole), 126.2 (*C*H–Triazole), 106.6 (C_{Fuc}-1, $^1J_{C,H}$ = 154 Hz), 101.5 (C_{GlcNAc}-1, $^1J_{C,H}$ = 160 Hz), 85.3 (C_{GlcNAc}-3), 77.6 (C_{GlcNAc}-5), 75.2, 73.1, 73.1, 72.1, 72.1, 71.6, 71.6, 71.6, 71.4, 71.1 (C_{GlcNAc}-4, C_{Fuc}-2, C_{Fuc}-3, C_{Fuc}-4, C_{Fuc}-5, 5 × Alkyl *C*H_2), 70.4 (triazole–CH_2–*C*H_2), 62.7, 62.6 (C_{GlcNAc}-6, *C*H_2–C=CH), 56.0 (C_{GlcNAc}-2), 51.8 (N_3–*C*H_2), 51.4 (triazole–*C*H_2), 23.4 (CO*C*H_3), 17.1 (CH_3).

II.5 Synthesis of the clickable trimannose

The clickable trimannose was synthesized as previously described (see Figure S25).[10]

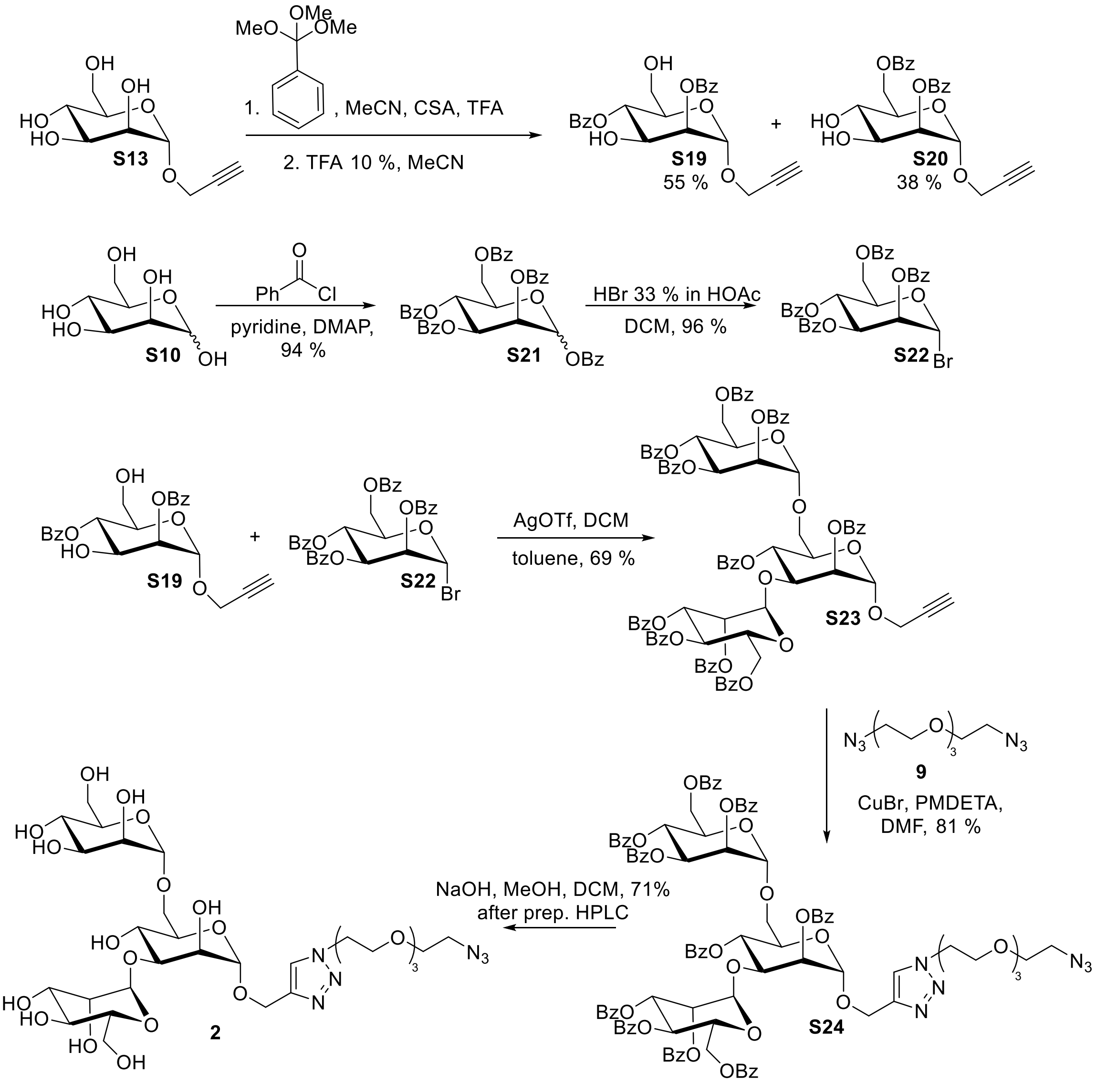

Figure S25: Synthesis of (1-(2-(2-(2-(2-Azidoethoxy)ethoxy)ethoxy)ethyl)-1*H*-1,2,3-triazol-4-yl)methoxy)-3,6-di-*O*-α-D-mannopyranosyl-α-D-mannopyranose (**2**).

II.6 Synthesis of clickable glycodendrons

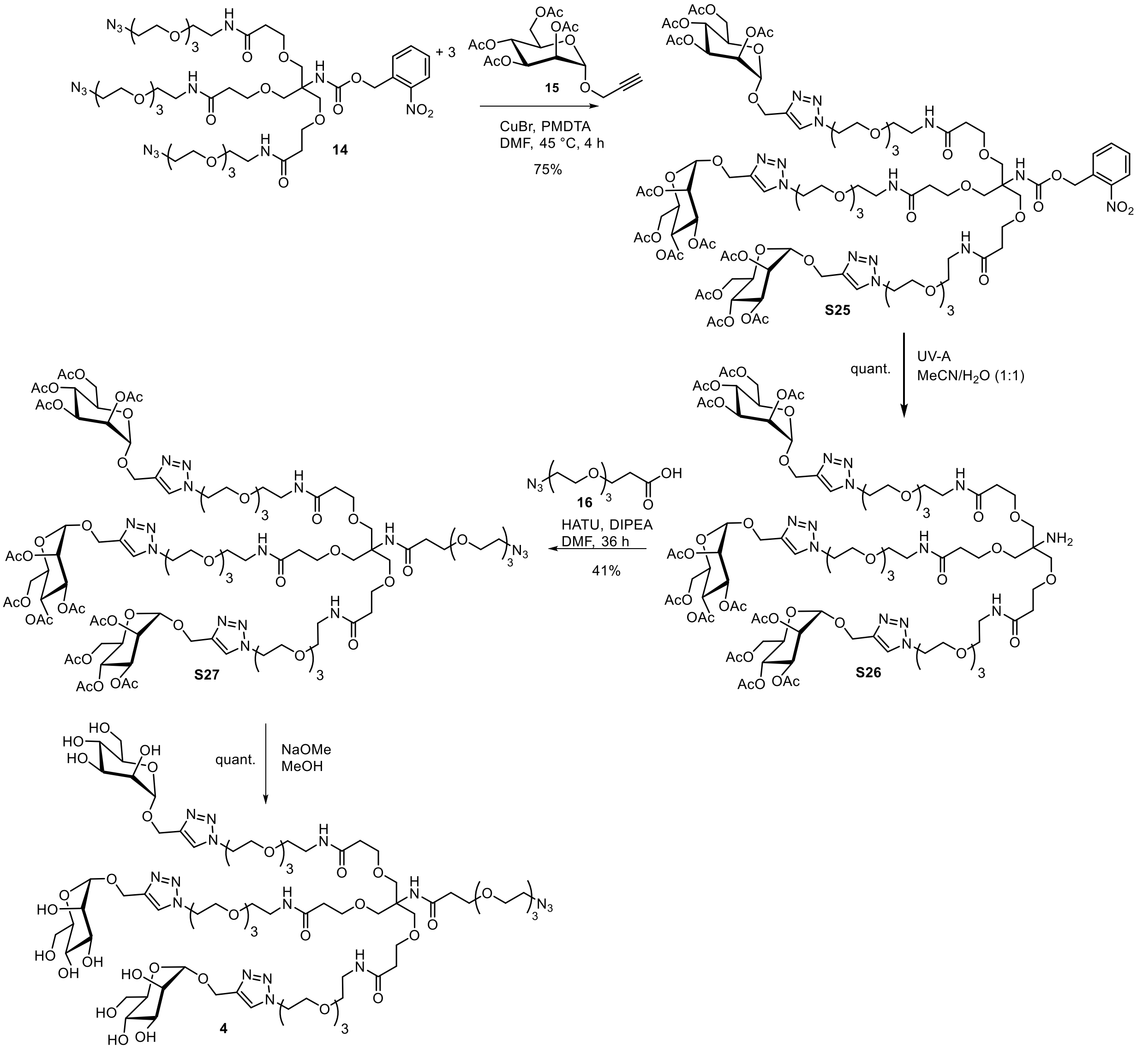

Figure S26: Synthesis of the mannose glycodendron **4**.

o-Nitrobenzyl (1,3,5-bis((4-(2,3,4,6-tetra-*O*-acetyl-α-D-mannopyranosyl)-methyl)-1H-1,2,3-triazol-1-yl)-18-(17-((4-(2,3,4,6-tetra-*O*-acetyl-α-D-mannopyranosyl))-methyl)-5-oxo-2,9,12,15-tetraoxo-6-azaheptadec-1-yl)-13,23-dioxo-3,6,9,16,20,27,30,33-octaoxa-12,24-diazapentatriacontan-18-yl)-carbamate (S25)

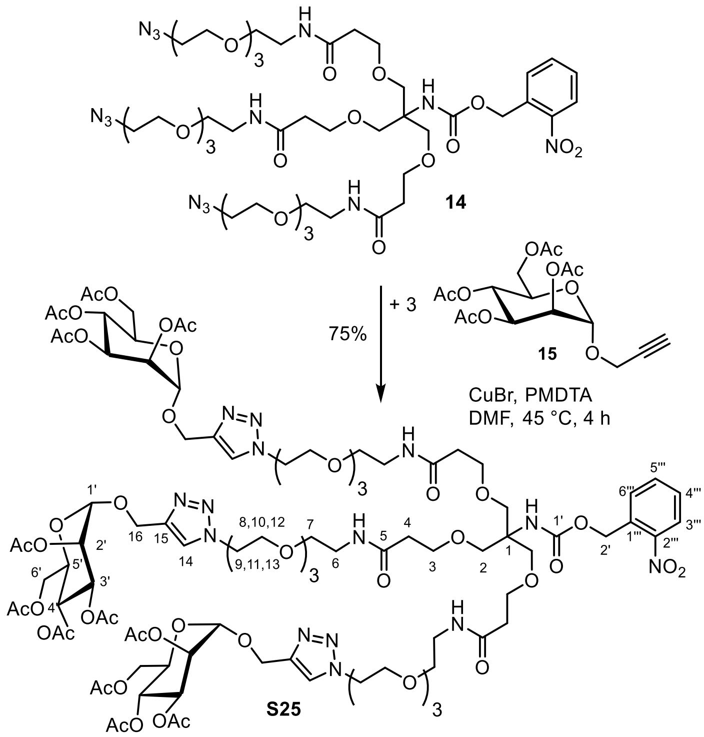

Figure S27: Synthesis of *o*-Nitrobenzyl (1,3,5-bis((4-(2,3,4,6-tetra-*O*-acetyl-α-D-mannopyranosyl)-methyl)-1H-1,2,3-triazol-1-yl)-18-(17-((4-(2,3,4,6-tetra-*O*-acetyl-α-D-mannopyranosyl))-methyl)-5-oxo-2,9,12,15-tetraoxo-6-azaheptadec-1-yl)-13,23-dioxo-3,6,9,16,20,27,30,33-octaoxa-12,24-diazapentatriacontan-18-yl)-carbamate (**S25**).

This compound was synthesized after a modified procedure by *Yamamoto et al.*[19]

Triazide **14** (50.0 mg, 0.04 mmol, 1.00 eq.) and mannose **15** (53.6 mg, 0.14 mmol, 3.50 eq.) were dissolved in anhydrous DMF (3.0 mL) under argon atmosphere. PMDTA (15.9 µL, 0.08 mmol, 2.00 eq.) was added. The reaction mixture was fitted with an argon balloon and degassed for 30 min with ultrasonication. The reaction mixture was heated to 45 °C and CuBr (2.00 mg, 0.01 mmol, 25mol%) was added. The reaction mixture was stirred for 2 h at 45 °C. Reaction control by TLC and LC-MS indicated complete conversion. The reaction mixture was diluted with EtOAc and extracted with sat. NH_4Cl solution (3x). The aqueous layers were combined and extracted with EtOAc (5x). The combined organic layers were dried over Na_2SO_4, filtered and all volatiles were removed in vacuo. The product was purified by preparative HPLC (MeCN/H_2O 45:55, R_T= 9.51 min.). The fractions containing the product were freeze dried and the product was obtained as a colorless oil.

Yield: 68.2 mg (0.03 mmol, 75 %), colorless oil.

R_f: = 0.29 (DCM/MeOH 15:1).

$C_{96}H_{142}N_{14}O_{49}$ (M = 2274.91 g/mol).

$[\alpha]_D$ = +57.1° (c = 1.25 °C, MeOH).

IR(ATR) $\tilde{\nu}$ (cm^{-1}) = 2254, 1749, 1372, 1227, 1090, 903, 724, 650.

ESI-MS: m/z (%) = 759.5 (100) $[M+3H]^{3+}$, 1139 (67.2) $[M+2H]^{2+}$, 1150 (9.03) $[M+Na]^{+}$

HR-MS: m/z =1160.4417 $[M+2Na]^{2+}$, calc. for $[C_{96}H_{142}N_{14}O_{49} + 2Na]^{2+}$ 1160.4417.

^{1}H-NMR, COSY, TOCSY (600 MHz, $CDCl_3$): δ (ppm) = 8.10-8.09 (m, 1H, H-3''), 7.77 (s, 3H, H-14), 7.68-7.65 (m, 2H, H-5'', H-6''), 7.50–7.45 (m, 1H, H-4''), 6.72 (t, *J* = 5.5 Hz, 3H, -(C-5=O)-N*H*-), 5.74 (s, 1H, -(C-1'=O)-N*H*-), 5.44 (s, 2H, H-2'), 5.35–5.26 (m, 6H, H-3', H-4'), 5.23 (t, *J* = 2.0 Hz, 3H, H-2'), 4.97 (d, *J* = 2.0 Hz, 3H, H-1'), 4.83 (d, *J* = 12.2 Hz, 3H, H-16), 4.67 (d, *J* = 12.2 Hz, 3H, H-16), 4.56 (t, *J* = 5.1 Hz, 6H, H-13), 4.31 (dd, *J* = 12.2, 5.0 Hz, 3H, H-6'), 4.11 (dd, *J* = 12.2, 2.6 Hz, 3H, H-6'), 4.08 (dd, *J* = 5.0, 2.6 Hz, 3H, H-5'), 3.89 (t, *J* = 5.1 Hz, 6H, H-12), 3.69 (t, *J* = 5.9 Hz, 6H, H-3), 3.64 (s, 6H, H-2), 3.63–3.58 (m, 24H, H-8, H-9, H-10, H-11), 3.54 (t, *J* = 5.5 Hz, 6H, H-7), 3.42 (q, *J* = 5.5 Hz, 6H, H-6), 2.41 (t, *J* = 5.9 Hz, 6H, H-4), 2.15 (s, 9H, -C-2'-O-(C=O)-CH_3), 2.12 (s, 9H, -C-6'-O-(C=O)-CH_3), 2.03 (s, 9H, -C-4'-O-(C=O)-CH_3), 1.97 (s, 9H, -C-3'-O-(C=O)-CH_3).

^{13}C-NMR, HSQC, HMBC (151 MHz, $CDCl_3$): δ (ppm) = 171.4 (C-5), 170.9 (-C-6'-O-(*C*=O)-CH_3), 170.3 (-C-2'O-(*C*=)-CH_3), 170.1 (-C-3'-O-(*C*=O)-CH_3), 169.9 (-C-4'-O-(*C*=O)-CH_3), 153.8 (C-1'), 147.3 (C-2''), 143.4 (C-15), 134.0 (C-5''), 134.1 (C-1''), 129.8 (C-6''), 128.9 (C-4''), 125.1 (C-3''), 124.4 (C-14), 96.9 (C-1'), 70.6 (C-11), 70.6 (C-10), 70.6 (C-9), 70.3 (C-8), 69.9 (C-7), 69.5 (C-12), 69.5 (C-2'), 69.4 (C-2), 69.2 (C-4'), 68.8 (C-5'), 67.5 (C-3), 66.1 (C-3'), 63.0 (C-2'), 62.5 (C-6'), 61.0 (C-16), 59.1 (C-1), 50.4 (C-13), 39.3 (C-6), 36.8 (C-4), 21.1 (-C-2'-O-(C=O)-*C*H_3), 21.0 (-C-6'-O-(C=O)-*C*H_3), 20.9 (-C-3'-O-(C=O)-*C*H_3), 20.9 (-C-4'-O-(C=O)-*C*H_3).

***N*-(1-((4-(2,3,4,6-tetra-*O*-acetyl-α-D-mannopyranosyl)-methyl)-1*H*-1,2,3-triazol-1-yl)-3,6,9-trioxa-undec-11-yl)-6-amino-6-(17-((4-(2,3,4,6-tetra-*O*-acetyl-α-D-mannopyranosyl)-methyl)-1*H*-1,2,3-triazol-1-yl)-5-oxo-2,9,12,15-tetraoxa-6-azaheptadec-1-yl)-11-oxo-4,8,15,18,21-pentaoxa-12-azatricosanamide (S26)**

C97H152N16O49 Na+

Na+

Figure S28: Synthesis of *N*-(1-((4-(2,3,4,6-tetra-*O*-acetyl-α-D-mannopyranosyl)-methyl)-1*H*-1,2,3-triazol-1-yl)-3,6,9-trioxa-undec-11-yl)-6-amino-6-(17-((4-(2,3,4,6-tetra-*O*-acetyl-α-D-mannopyranosyl)-methyl)-1*H*-1,2,3-triazol-1-yl)-5-oxo-2,9,12,15-tetraoxa-6-azaheptadec-1-yl)-11-oxo-4,8,15,18,21-pentaoxa-12-azatricosanamide (S26)

Glycodendron **S25** (24.0 mg, 0.01 mmol, 1.00 eq.) was dissolved in water (1.0 mL) and MeCN (1.0 mL). The solution was degassed with argon with ultrasonication. The reaction was afterwards fitted onto the Rayonet photoreactor (equipped with UV-A bulbs) and irradiated for 15 h. Reaction control by LC-MS indicated complete conversion to the product. The reaction mixture was freeze dried and the product was obtained as a brown oil.

Yield: 22.0 mg (0.01 mmol, quant.), (Lit.)[20]: quant., brown oil.

R_f: = 0.43 (DCM/MeOH 5:1).

$C_{88}H_{137}N_{13}O_{45}$ (M = 2095.88 g/mol).

[α]_D = not detectable, the compounds forms a colored solution, in which the optical rotation cannot be determined due to strong absoprtion.

IR(ATR): $\tilde{\nu}$ (cm^{-1}) = 2875, 1749, 1661, 1540, 1458, 1371, 1227, 1133, 1088, 1049, 981, 903, 726, 668, 650.

ESI-MS: m/z (%) = 1049.3 (100) $[M+2H]^{2+}$, 699.8 (55.4) $[M+3H]^{3+}$

HR-MS: m/z = 1070.9305 $[M+2Na]^{2+}$, calc. for $[C_{88}H_{137}N_{13}O_{45} + 2Na]^{2+}$ 1170.9308.

^{1}H-NMR, COSY, TOCSY (600 MHz, $CDCl_3$): δ (ppm) = 7.78 (s, 3H, H-14), 5.33–5.27 (m, 6H, H-3', H-4'), 5.23 (t, *J* = 2.0 Hz, 3H, H-2'), 4.97 (d, *J* = 2.0 Hz, 3H, H-1'), 4.83 (d, *J* = 12.2 Hz, 3H, H-16), 4.67 (d, *J* = 12.2 Hz, 3H, H-16), 4.56 (t, *J* = 5.2 Hz, 6H, H-13), 4.31 (dd, *J* = 12.2, 5.0 Hz, 3H, H-6'),4.11 (dd, *J* = 12.2, 2.5 Hz, 3H, H-6'), 4.08 (dd, *J* = 5.0, 2.5 Hz, 3H, H-5'), 3.91–3.85 (m, 6H, H-12),3.72-3.70 (m, 6H, H-3), 3.62-3.61 (m, 6H, H-2), 3.61–3.57 (m, 24H, H-8, H-9, H-10, H-11), 3.55 (t, *J* = 5.4 Hz, 6H, H-7), 3.43 (d, *J* = 5.4 Hz, 6H, H-6), 2.42 (s, 6H, H-4), 2.15 (s, 9H, -C-2'-O-(C=O)-CH_3), 2.12 (s, 9H, -C-6'-O-(C=O)-CH_3), 2.04 (s, 9H, -C-4'-O-(C=O)-CH_3), 1.98 (s, 9H, -C-3'-O-(C=O)-CH_3).

^{13}C-NMR, HSQC, HMBC (151 MHz, $CDCl_3$): δ (ppm) = 171.4 (C-5), 170.8 (-C-6'-O-(*C*=O)-CH_3), 170.1 (-C-2'-O-(*C*=O)-CH_3), 170.0 (-C-4'-O-(*C*=O)-CH_3), 169.8 (-C-3'-O-(*C*=O)-CH_3), 143.3 (C-15), 124.3 (C-14), 96.8 (C-1'), 70.5 (C-2), 70.5 (-*C*H_2-), 70.5 (-*C*H_2-), 70.4 (-*C*H_2-), 70.0 (-*C*H_2-), 69.8 (C-7), 69.4 (C-12), 69.4 (C-2'), 69.1 (C-4'), 68.7 (C-5'), 67.5 (C-3), 66.0 (C-3'), 62.4 (C-6'), 60.9 (C-16), 50.3 (C-13), 39.2 (C-6), 36.6 (C-4), 21.0 (-C-2'-O-(C=O)-CH_3), 20.9 (-C-6'-O-(C=O)-*C*H_3), 20.8 (-C-4'-O-(C=O)-*C*H_3), 20.8 (-C-3'-O-(C=O)-*C*H_3).

N-(1-bis(17-((4-(2,3,4,6-tetra-*O*-acetyl-α-D-mannopyranosyl)-methyl)-1*H*-1,2,3-triazol-1-yl)-5-oxo-2,9,12,15-tetraoxa-6-azaheptadec-1-yl)-18-((4-(2,3,4,6-tetra-*O*-acetyl-α-D-mannopyranosyl)-methyl)-1*H*-1,2,3-triazol-1-yl)-6-oxo-3,10,13,16-tetraoxa-7-azaoctadec-1-yl)-12-azido-4,7,10-pentaoxadodecanamide (S27)

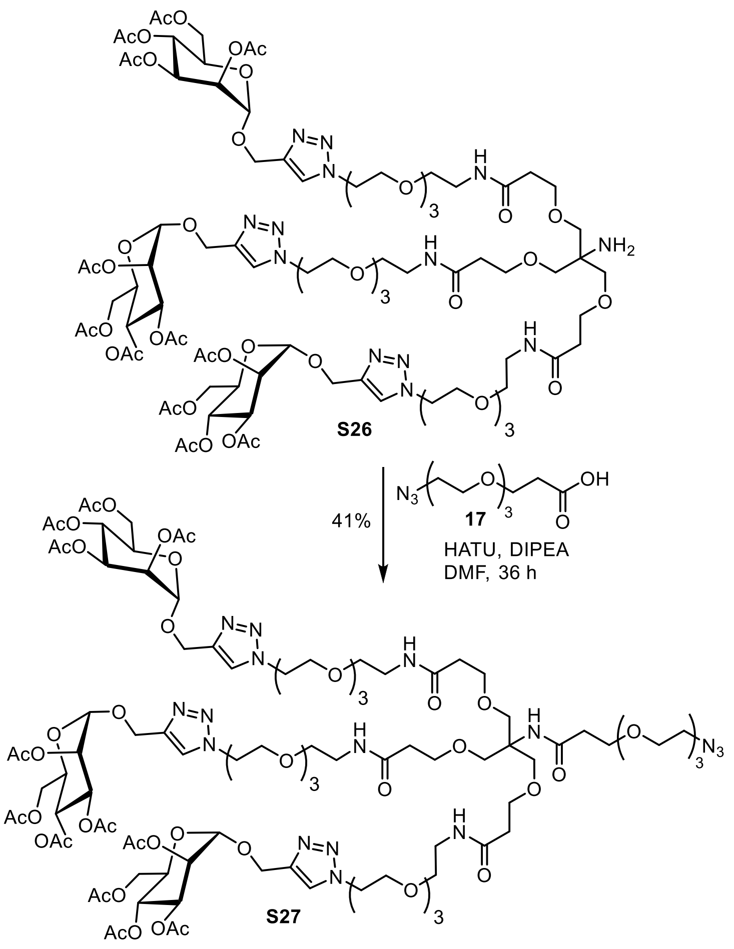

Figure S29: Synthesis of *N*-(1-bis(17-((4-(2,3,4,6-tetra-*O*-acetyl-α-D-mannopyranosyl)-methyl)-1*H*-1,2,3-triazol-1-yl)-5-oxo-2,9,12,15-tetraoxa-6-azaheptadec-1-yl)-18-((4-(2,3,4,6-tetra-*O*-acetyl-α-D-mannopyranosyl)-methyl)-1*H*-1,2,3-triazol-1-yl)-6-oxo-3,10,13,16-tetraoxa-7-azaoctadec-1-yl)-12-azido-4,7,10-pentaoxadodecanamide (**S27**).

Linker **17** (144 mg, 0.58 mmol, 10.00 eq.), HATU (222 mg, 0.58 mmol, 10.00 eq.) and DIPEA (0.14 mL, 0.75 mmol, 13.00 eq.) were dissolved in anhydrous DMF (1.00 mL) under argon atmosphere. The reaction mixture was stirred at room temperature for 30 min. Amine **S26** (122 mg, 0.06 mmol, 1.00 eq.) was dissolved in DMF (1.00 mL) under argon atmosphere, taken up by syringe and added to the first reaction mixture in a dropwise fashion. Reaction control by LC-MS indicated complete conversion. All volatiles were removed in vacuo and the residue was co-distilled with toluene (3x). The crude product was purified by preparative HPLC chromatography (MeCN/H_2O 60% isocratic). The fractions containing the product were freeze dried and the product was obtained as a colorless oil.

Yield: 57.2 mg ($24.6 \cdot 10^{-3}$ mmol, 41 %), (Lit.)[1]: 61 %, colorless oil.

R_f: = 0.25 (DCM/MeOH 15:1).

$C_{97}H_{152}N_{16}O_{49}$ (M = 2324.99 g/mol).

$[\alpha]_D$ = +24.0° (c = 1, 25 °C, MeOH).

IR(ATR): $\tilde{\nu}$ (cm^{-1}) = 3348, 2874, 2108, 1745, 1657, 1545, 1433, 1370, 1224, 1131, 1086, 1048, 980, 918, 732, 601.

ESI-MS: m/z (%) = 776.2 (100) $[M+3H]^{3+}$.

HR-MS: m/z = 1185.4801 $[M + 2Na]^{2+}$, calc. for $[C_{97}H_{152}N_{16}O_{49} + 2Na]^{2+}$ 1185.4839.

^{1}H-NMR, COSY, TOCSY (600 MHz, $CDCl_3$): δ (ppm) = 7.77 (s, 3H, H-14), 6.81 (t, *J* = 5.6 Hz, 3H, -(C-5=O)-N*H*-), 6.60 (s, 1H, -(C-1''=O)-N*H*-), 5.31–5.29 (m, 6H, H-3', H-4'), 5.23 (t, *J* = 2.3 Hz, 3H, H-2'), 4.96 (d, *J* = 1.7 Hz, 3H, H-1'), 4.83 (d, *J* = 12.2 Hz, 3H, H-16), 4.67 (d, *J* = 12.2 Hz, 3H, H-16), 4.58–4.54 (m, 6H, H-13), 4.30 (dd, *J* = 12.2, 5.0 Hz, 3H, H-6'), 4.11 (dd, *J* = 12.2, 2.6 Hz, 3H, H-6'), 4.09-4.07 (m, 3H, H-5'), 3.90 (t, *J* = 5.1 Hz, 6H, H-12), 3.71–3.59 (m, 48H, H-2, H-3, H-8, H-9, H-10, H-11, H-3'', H-4'', H-5'', H-6'', H-7'', H-8''), 3.54 (t, *J* = 5.6 Hz, 6H, H-7), 3.43 (q, *J* = 5.6 Hz, 6H, H-6), 3.40–3.37 (m, 2H, H-9''), 2.44 (t, *J* = 6.1 Hz, 2H, H-2''), 2.41 (t, *J* = 5.9 Hz, 6H, H-4), 2.15 (s, 9H, -C-2'-O-(C=O)-CH_3), 2.12 (s, 9H, -C-6'-O-(C=O)-CH_3), 2.03 (s, 9H, -C-3'-O-(C=O)-CH_3), 1.97 (s, 9H, -C-4'-O-(C=O)-CH_3).

^{13}C-NMR, HSQC, HMBC (151 MHz, $CDCl_3$): δ (ppm) = 171.8 (C-1''), 171.5 (C-5), 170.9 (-C-6'-O-(*C*=O)-CH_3), 170.2 (-C-2'-O-(*C*=O)-CH_3), 170.1 (-C-4'-O-(*C*=O)-CH_3), 169.9 (-C-3'-O-(*C*=O)-CH_3), 143.4 (C-15), 124.4 (C-14), 96.9 (C-1'), 70.8 (-*C*H_2-), 70.6 (-*C*H_2-), 70.6 (-*C*H_2-), 70.6 (-*C*H_2-), 70.6 (-*C*H_2-), 70.3 (-*C*H_2-), 70.3 (-*C*H_2-), 70.1 (-*C*H_2-), 70.0 (C-7), 69.5 (-CH_2-), 69.5 (C-2), 69.5 (C-12), 69.4 (C-2') , 69.2 (C-4'), 68.8 (C-5'), 67.5 (C-3), 67.4 (C-3''), 66.1 (C-3'), 62.5 (C-6'), 61.0 (C-16), 59.9 (C-1), 50.8 (C-9''), 50.4 (C-13), 39.3 (C-6), 37.5 (C-2''), 36.7 (C-4), 21.1 (-C-2'-O-(C=O)-*C*H_3), 21.0 (-C-6'-O-(C=O)-*C*H_3), 20.9 (-C-3'-O-(C=O)-*C*H_3), 20.9 (-C-4'-O-(C=O)-*C*H_3).

N-(1-bis(17-((4-(α-D-mannopyranosyl)-methyl)-1*H*-1,2,3-triazol-1-yl)-5-oxo-2,9,12,15-tetraoxa-6-azaheptadec-1-yl)-18-((4-(α-D-mannopyranosyl)-methyl)-1*H*-1,2,3-triazol-1-yl)-6-oxo-3,10,13,16-tetraoxa-7-azaoctadec-1-yl)-12-azido-4,7,10-pentaoxadodecanamide (4)

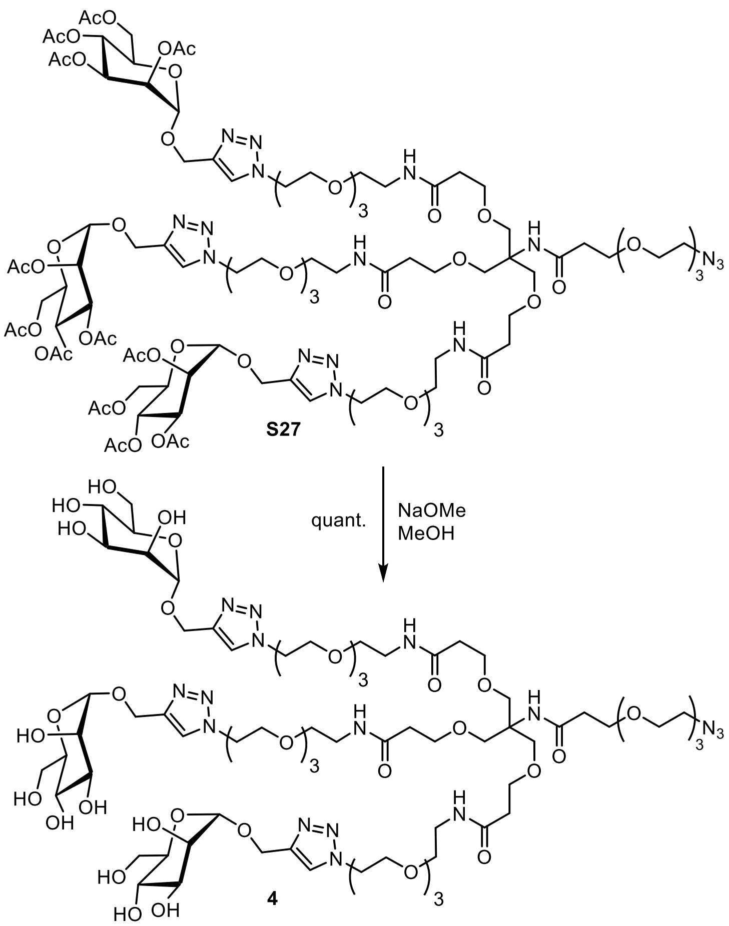

Figure S30: Synthesis of *N*-(1-bis(17-((4-(α-D-mannopyranosyl)-methyl)-1*H*-1,2,3-triazol-1-yl)-5-oxo-2,9,12,15-tetraoxa-6-azaheptadec-1-yl)-18-((4-(α-D-mannopyranosyl)-methyl)-1*H*-1,2,3-triazol-1-yl)-6-oxo-3,10,13,16-tetraoxa-7-azaoctadec-1-yl)-12-azido-4,7,10-pentaoxadodecanamide (**4**).

Glycodendron **S27** (57.2 mg, 0.003 mmol, 1.00 eq.) was dissolved in methanol (3.0 mL). A catalytic amount of sodium methoxide was added. The reaction was stirred at room temperature for 2 h until complete conversion was indicated by TLC. Amberlite H^+-resin was added until a neutral pH was detected. The reaction mixture was filtered over celite and all volatiles were removed in vacuo. The product was received as a colorless oil.

Yield: 44.8·mg (24.6·10^{-3} mmol, quant.), (Lit.)[21]: quant., colorless oil.

***Rf*:** = 0.0 (DCM/MeOH 10:1).

$C_{73}H_{128}N_{16}O_{37}$ (M = 1820,86 g/mol).

$[\alpha]_D$ = +19.3° (c = 1, 24 °C, MeOH).

IR(ATR): $\tilde{\nu}$ (cm^{-1}) = 3313, 2878, 2349, 1657,1598, 1572, 1562, 1478, 1099, 815, 671.

ESI-MS: m/z (%) = 933.8 (100) $[M+ 2Na]^{2+}$.

HR-MS: m/z = 933.4181 $[M + 2Na]^{2+}$, calc. for $[C_{73}H_{128}N_{16}O_{37} + 2Na]^{2+}$ 933.4206.

^{1}H-NMR, COSY, TOCSY (600 MHz, D_2O): δ (ppm) = 8.08 (s, 3H, H-14), 4.92 (d, *J* = 1.7 Hz, 3H, H-1'),4.77 (d, *J* = 12.5 Hz, 3H, H-16), 4.66 (d, *J* = 12.5 Hz, 3H, H-16), 4.59 (t, *J* = 5.0 Hz, 6H, H-13), 3.93 (t, *J* = 5.0 Hz, 6H, H-12), 3.87 (dd, *J* = 3.5, 1.7 Hz, 3H, H-4'), 3.82–3.77 (m, 3H, H-6'), 3.74–3.69 (m, 6H, H-5', H-6'), 3.68–3.52 (m, 60H, H-2, H-3, H-7, H-8, H-9, H-10, H-11, H-2', H-3', H-3'', H-4'', H-5'', H-6'', H-7'', H-8''), 3.44–3.42 (m, 2H, H-9''), 3.34 (t, *J* = 5.4 Hz, 6H, H-6), 2.45-2.44 (m, 8H, H-4, H-2'').

^{13}C-NMR, HSQC, HMBC (151 MHz, D_2O): δ (ppm) = 174.0 (C-5), 174.0 (C-1''), 143.4 (C-15), 125.6 (C-14), 99.3 (C-1'), 72.9 (C-2'), 70.4 (C-5'), 69.9 (C-4'), 69.5 (-*C*H2-), 69.5 (-*C*H2-), 69.5 (-*C*H2-),69.5 (-*C*H2-), 69.4 (-*C*H2-), 69.4 (-*C*H2-), 69.4 (-*C*H2-), 69.4 (C-2), 69.2 (-*C*H2-), 68.7 (-*C*H2-), 68.7 (C-12),68.4 (-*C*H2-), 67.4 (C-3), 66.6 (C-3'), 66.6 (-*C*H2-), 60.7 (C-6'), 60.2 (C-1), 59.6 (C-16), 50.4 (C-9''),50.0 (C-13), 38.9 (C-6), 36.0 (C-4), 36.3 (C-2'').

II. Nanocapsule synthesis and functionalization

Synthesis of HES nanocapsules

The nanocapsules were prepared adopting to previously published procedures from Landfester et al. using an inverse miniemulsion process[22]

1400 mg of an aqueous HES solution (200 kDa, 0.5, 3%) was mixed with 1 mg of SR101 and 20 mg of sodium chloride (dispersed phase). The solution was stirred for 5 min at room temperature until all components were completely dissolved. That followed, 100 mg of the surfactant P(E/B-*b*-EO) was dissolved in 7.5 g of dry cyclohexane under shaking and added at once to the dispersed phase. The mixture was pre-emulsified by vigorously stirring for 1 h at room temperature. Afterwards the emulsion was subjected to ultrasound for 3 min (70%, 20 s pulse, 10 s pause) and subsequently placed in an oil bath at 25 °C equipped with a stirrer. Under stirring at 700 rpm, a mixture of 30 mg P(E/B-*b*-EO) and 100 mg TDI in 5.0 g dry cyclohexane was added dropwise with a syringe. The emulsion was stirred at 25 °C for 24 h.

Redispersion of HES nanocapsules into aqueous SDS solution

5 mL of nanocapsule dispersion was split into three Eppendorf tubes, 1.667 mL each and centrifuged for 30 min at 4000 rpm. The supernatant was replaced with fresh dry cyclohexane and the procedure was repeated. The pellets were redispersed in 500 µL fresh cyclohexane and added dropwise in a time period of 5 min into 5 mL of aqueous SDS solution (0.1 wt%) under constant sonication in an ultrasonic bath. The aqueous dispersions were placed on a stirring plate and the cyclohexane was evaporated at room temperature under vigorous stirring at 1000 rpm.

Functionalization of HES nanocapsules with DBCO-PEG$_4$-NHS ester and quantification of the DBCO groups on the nanocapsule surface

The aqueous nanocapsule dispersion (solid content of ca. 0.1 wt%) was centrifuged at 4000 rpm for 30 min and the supernatant was replaced by Ampuwa water. The dispersion was then concentrated tenfold by centrifugation. 10 mg of DBCO-PEG$_4$-NHS ester was dissolved in 500 µL dry DMSO. 165 µL of DBCO-PEG$_4$-NHS ester solution (3.30 mg, $5.079 \cdot 10^{-6}$ mol) was added per mL nanocapsule dispersion (1 wt%) and the mixture stirred in a glass vial over night at room temperature. Afterwards the dispersion was centrifuged at 8000 rpm for 30 min and the supernatant was replaced with fresh Ampuwa® water. The procedure was repeated with 5000 rpm centrifugation speed.

After functionalization and purification of the nanocapsules, the attached DBCO groups were quantified by a fluorescent assay. Therefore, freshly dissolved anthracene azide in DMSO was mixed with the DBCO modified nanocapsule dispersion and subjected to reaction overnight

on a shaker at room temperature in the dark. The data were calculated according to previously reported procedures.[23]

Functionalization of DBCO-modified HES nanocapsules with clickable carbohydrate derivatives

According to the calculated amount of attached DBCO groups on the nanocapsules, different concentrations of carbohydrates were added to the nanocapsule dispersion. Therefore, the carbohydrates were dissolved in MilliQ water and a threefold molar excess was added to 300-400 µL (approx. $1.38 \cdot 10^{17}$ DBCO groups/mL) of nanocapsule dispersion each. The dispersions were stirred at room temperature for 3 days. Excess carbohydrates were removed by dialysis (MWCO 14 kDa) against Ampuwa water and the samples were concentrated by centrifugation (6000 rpm, 30 min).

III. NMR Spectra

IV.1 NMR Spectra for the clickable dendrimer core

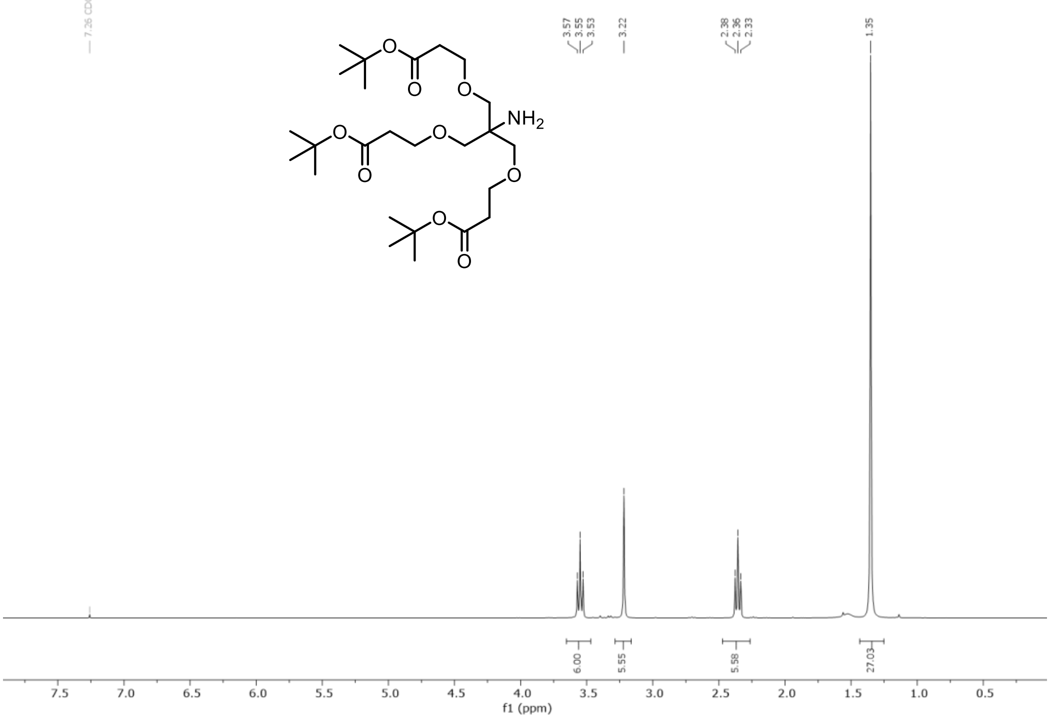

^{1}H-NMR (300 MHz, $CDCl_3$): Tris((2-(tert-butoxycarbonyl)ethoxyl)methyl)methylamine (**S1**).

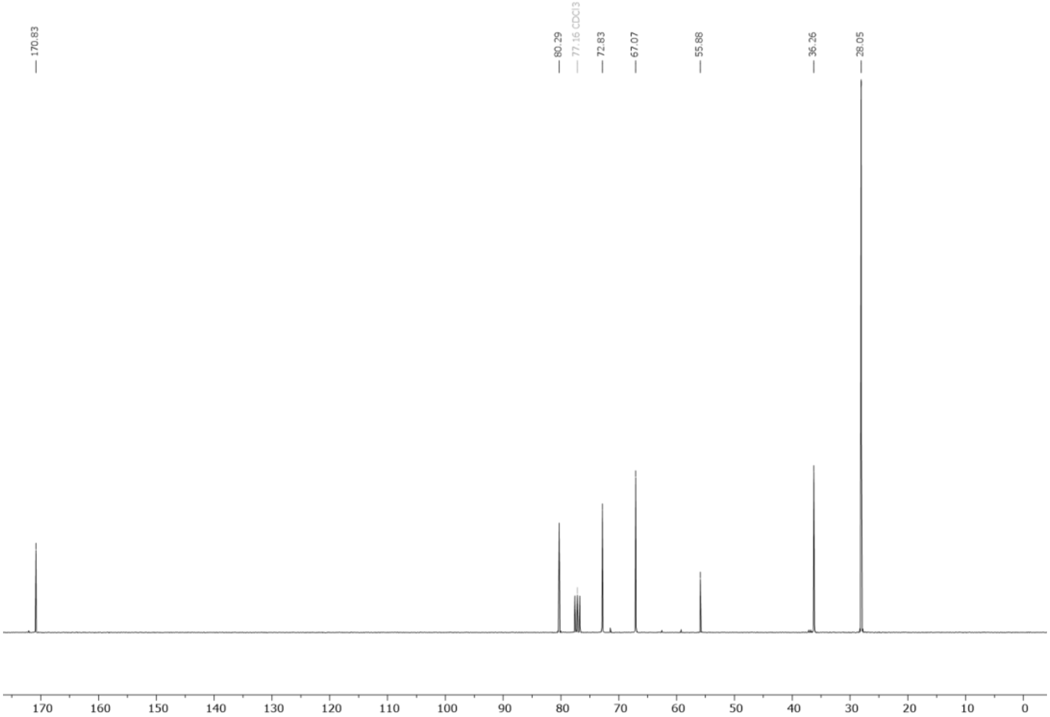

^{13}C-NMR (75 MHz, $CDCl_3$): Tris((2-(tert-butoxycarbonyl)ethoxyl)methyl)methylamine (**S1**).

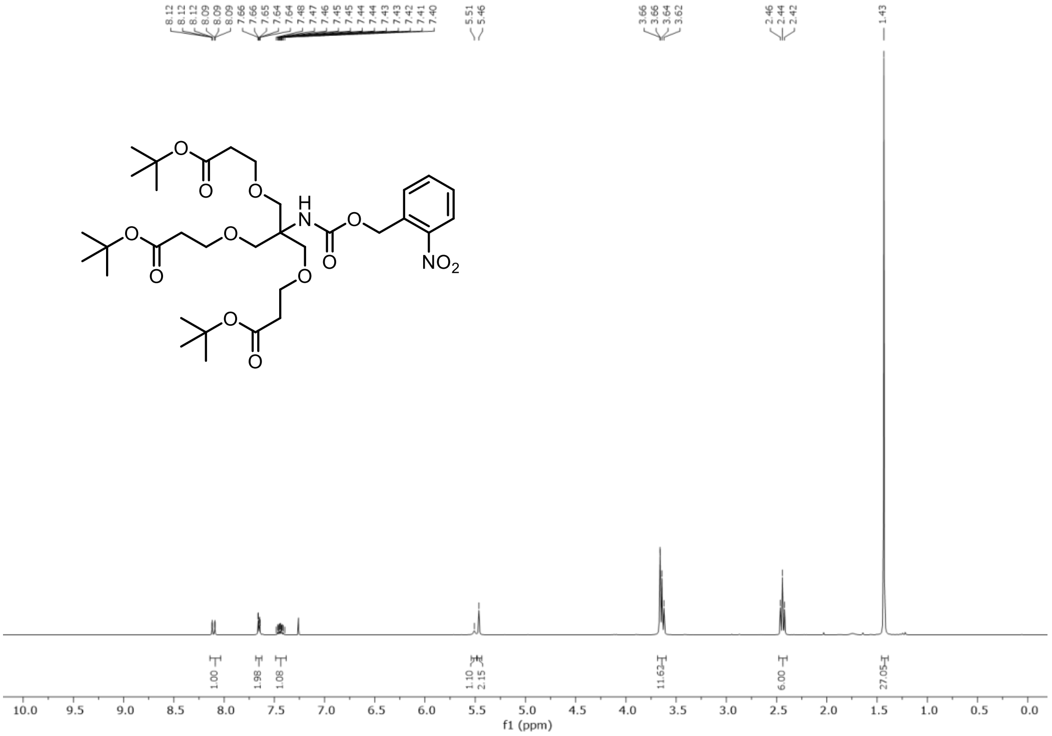

^{1}H-NMR (300 MHz, $CDCl_3$): *o*-Nitrobenzyl-(6-(1-*tert*-butyl-4-oxapentanoat-5-yl)-4,8-dioxa-1,11-di-*tert*-butylundecanoat-6-yl)-carbamate (**S2**).

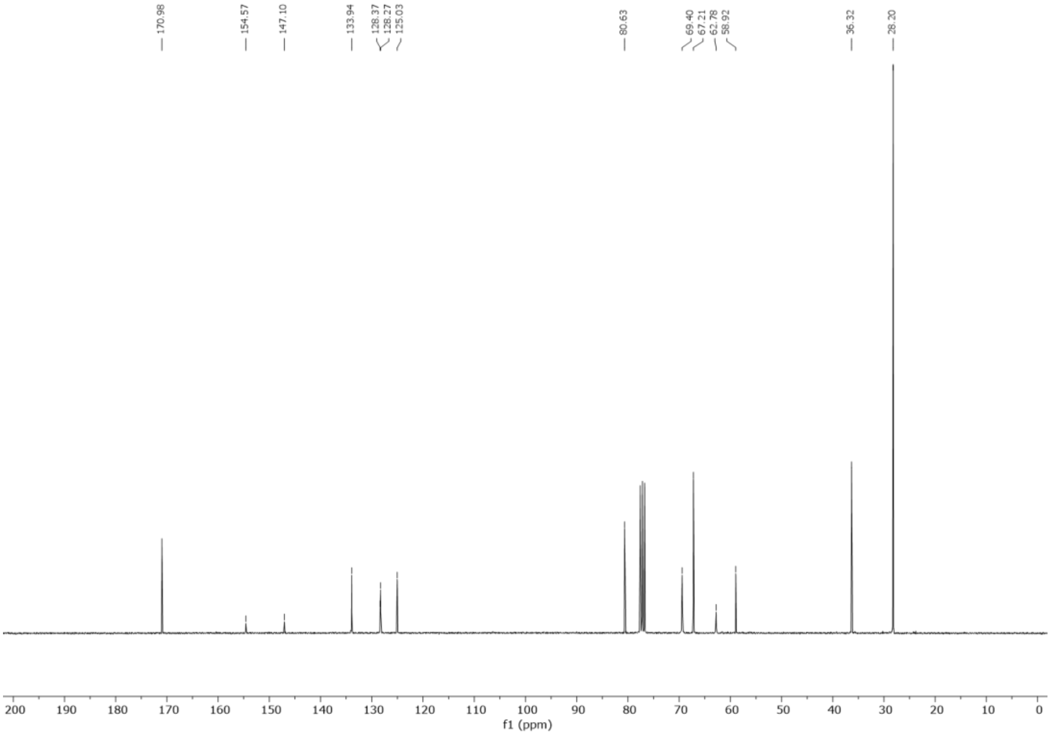

^{13}C-NMR (75 MHz, $CDCl_3$): *o*-Nitrobenzyl-(6-(1-*tert*-butyl-4-oxapentanoat-5-yl)-4,8-dioxa-1,11-di-*tert*-butylundecanoat-6-yl)-carbamate (**S2**).

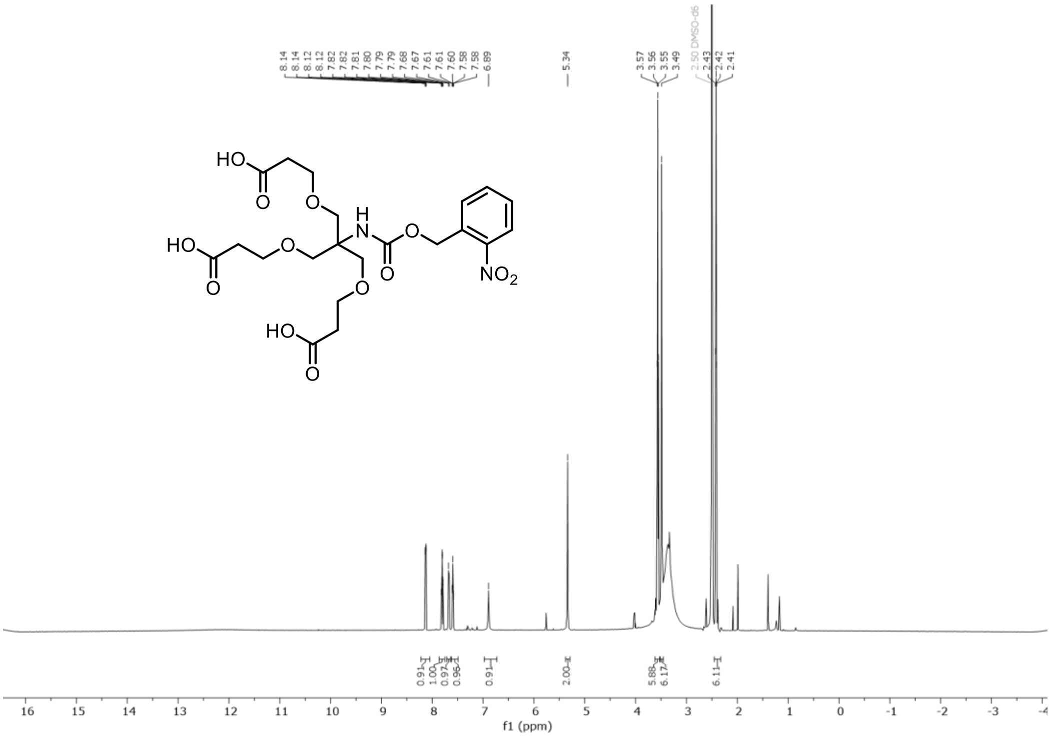

^{1}H-NMR (600 MHz, DMSO-d_6): *o*-Nitrobenzyl-(6-(1-carboxy-4-oxapentan-5-yl)-4,8-dioxa-1-11-dicarboxyundecan-6-yl)-carbamate (**S3**).

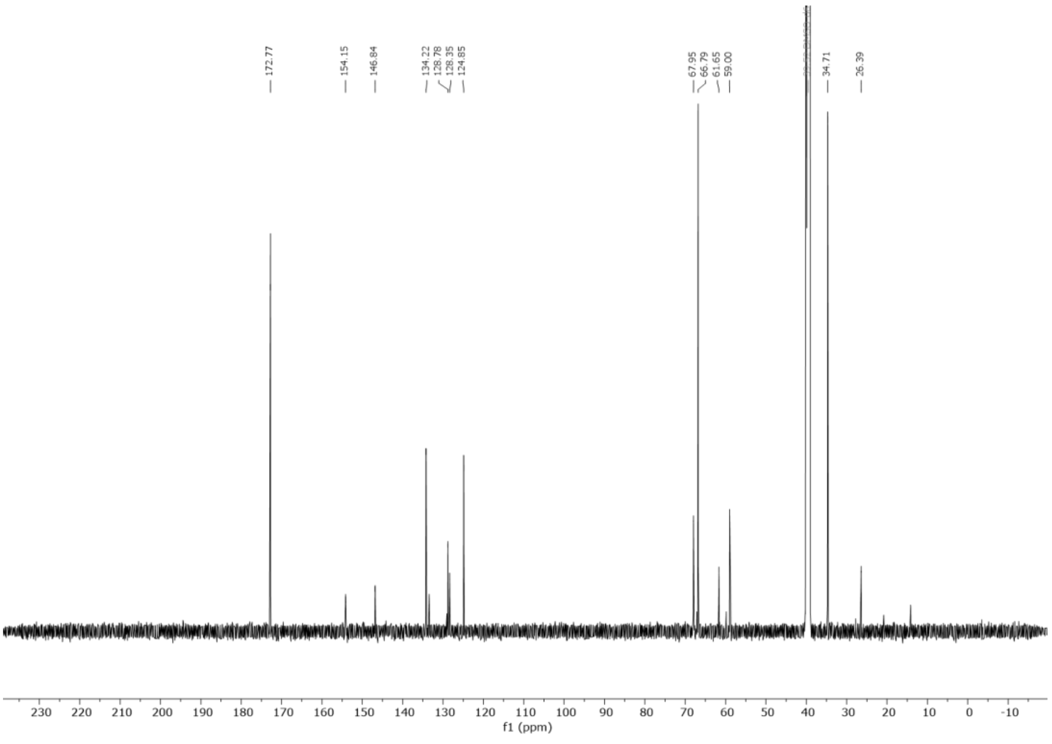

^{13}C-NMR (151 MHz, DMSO-d_6): *o*-Nitrobenzyl-(6-(1-carboxy-4-oxapentan-5-yl)-4,8-dioxa-1-11-dicarboxyundecan-6-yl)-carbamate (**S3**).

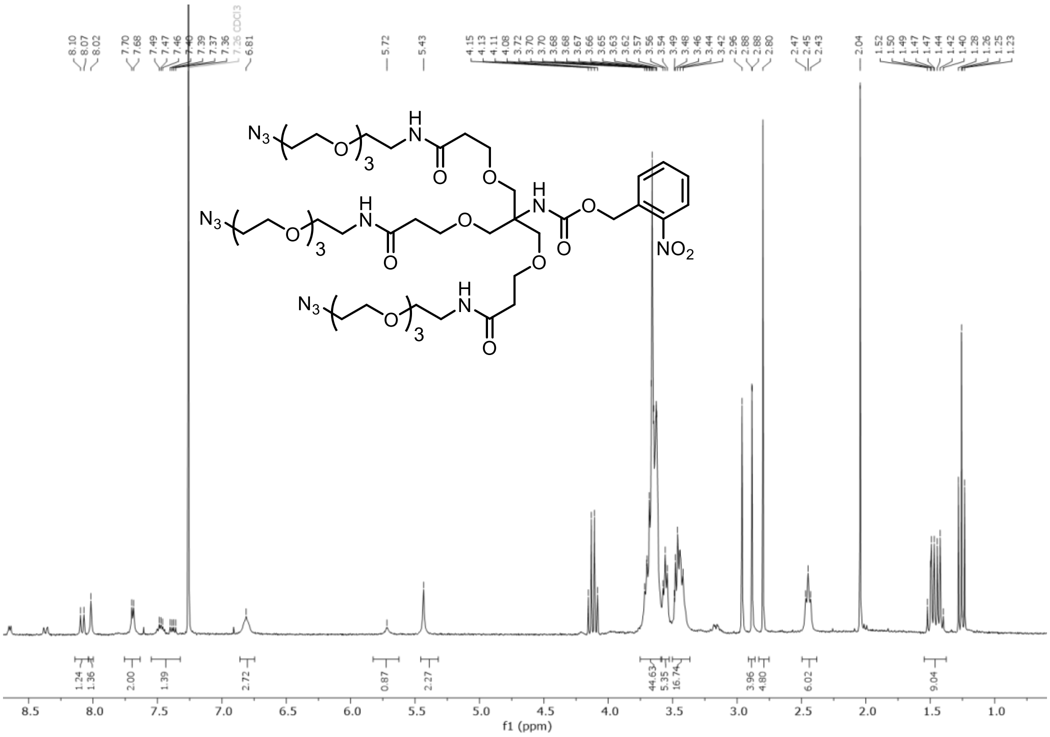

^{1}H-NMR (600 MHz, $CDCl_3$): *o*-Nitrobenzyl-(1,35-diazido-18-(17-azido-5-oxo-2,9,12,15-tetraoxa-6-azaheptadecyl)-13,23-dioxo-3,6,9,16,20,27,30,33-octaoxa-12,24-diazapentatriacontan-18-yl)-carbamate (**14**).

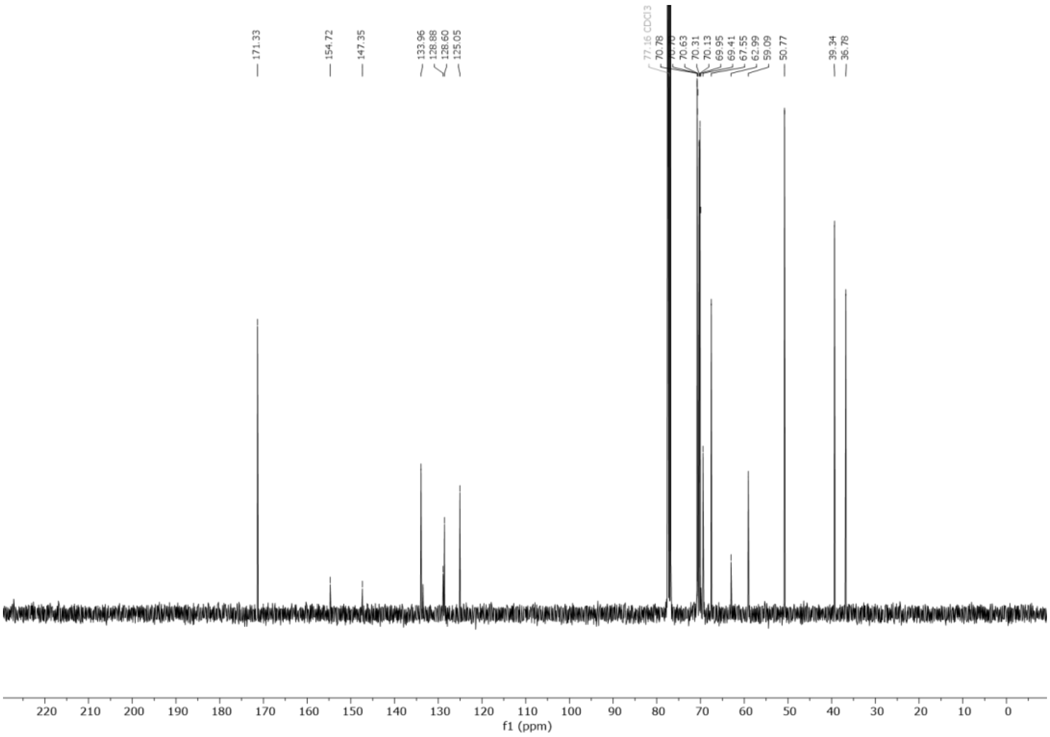

^{13}C-NMR (151 MHz, $CDCl_3$): *o*-Nitrobenzyl-(1,35-diazido-18-(17-azido-5-oxo-2,9,12,15-tetraoxa-6-azaheptadecyl)-13,23-dioxo-3,6,9,16,20,27,30,33-octaoxa-12,24-diazapentatriacontan-18-yl)-carbamate (**14**).

IV.3 NMR Spectra for the PEG spacer

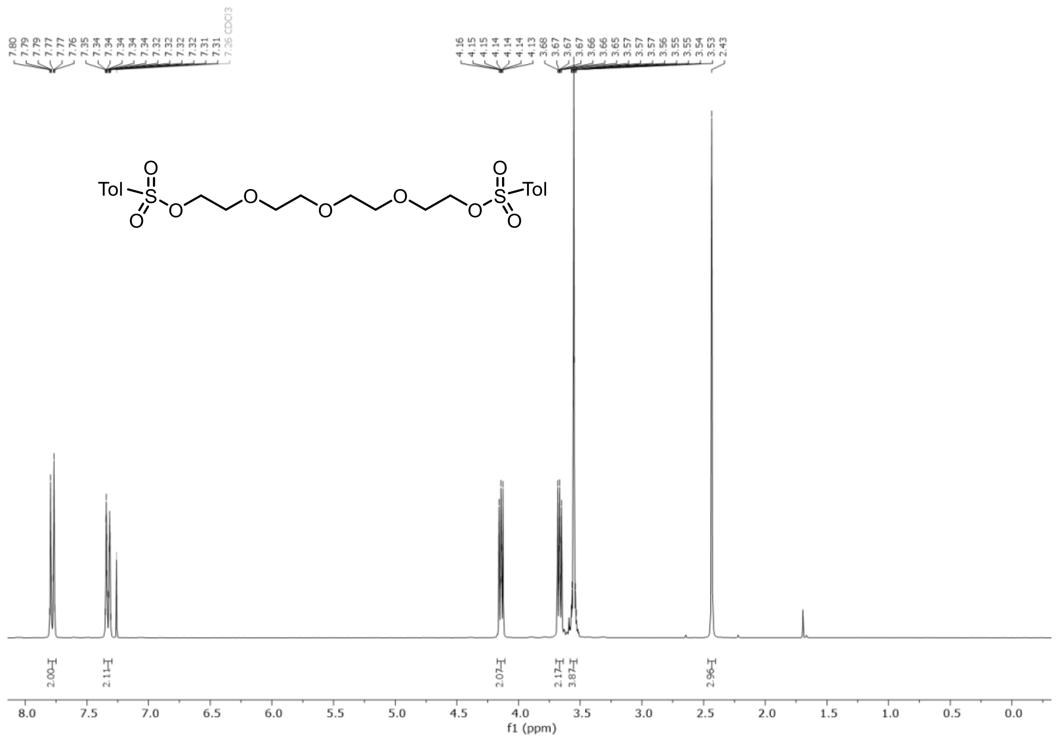

^{1}H-NMR (300 MHz, $CDCl_3$): 1,11-Ditosyl-3,6,9-trioxaundecane (**S5**).

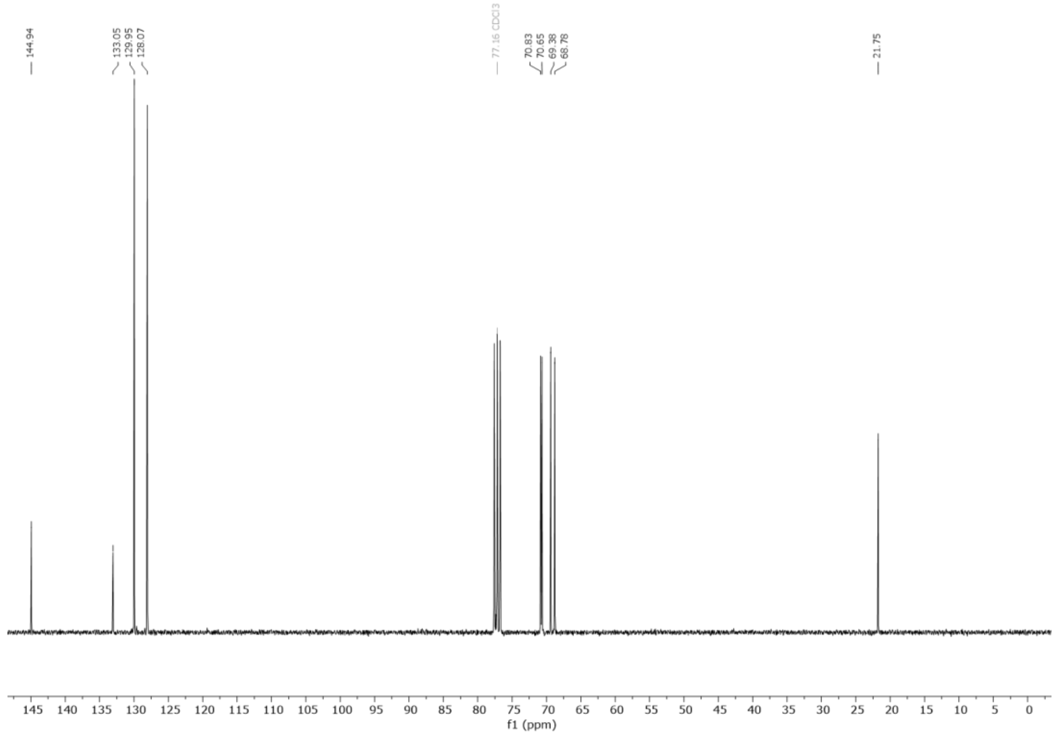

^{13}C-NMR (75 MHz, $CDCl_3$): 1,11-Ditosyl-3,6,9-trioxaundecane (**S5**).

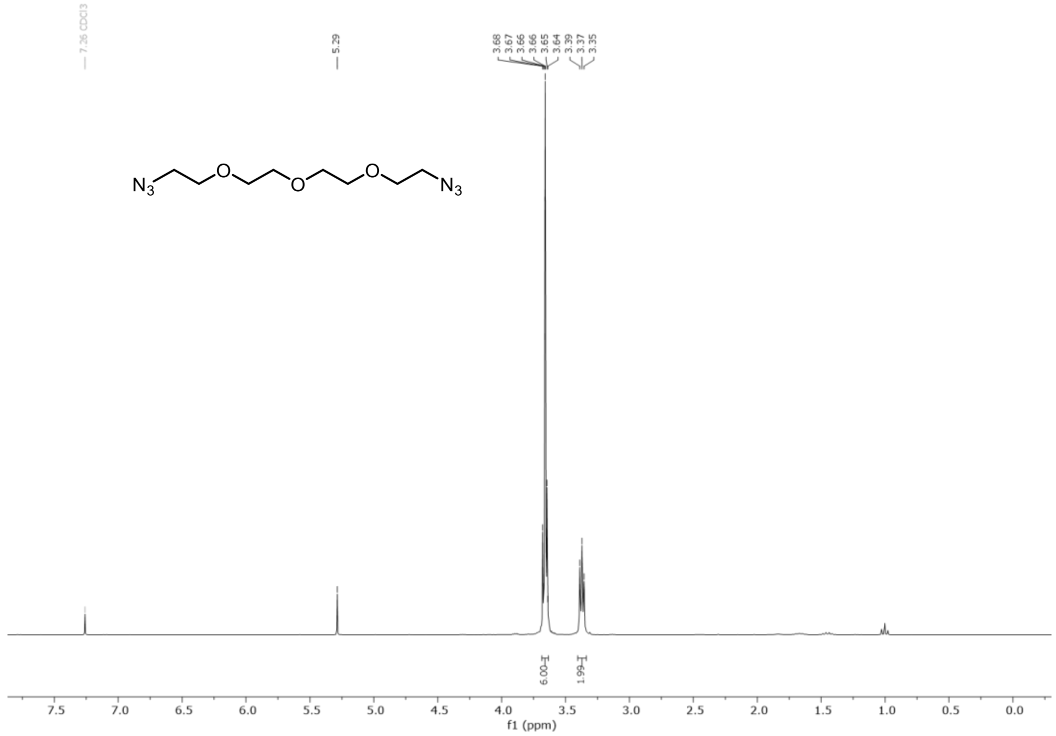

^{1}H-NMR (300 MHz, $CDCl_3$): 1-azido-2-(2-(2-(2-azidoethoxy)ethoxy)ethoxy)ethane (**9**).

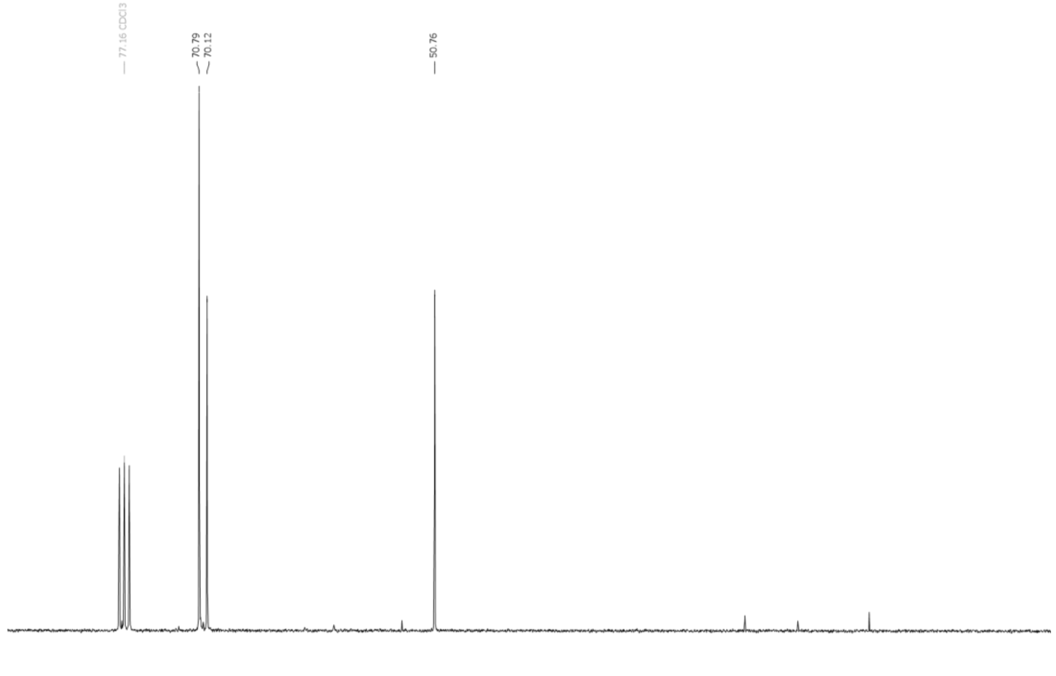

^{13}C-NMR (75 MHz, $CDCl_3$): 1-azido-2-(2-(2-(2-azidoethoxy)ethoxy)ethoxy)ethane (**9**).

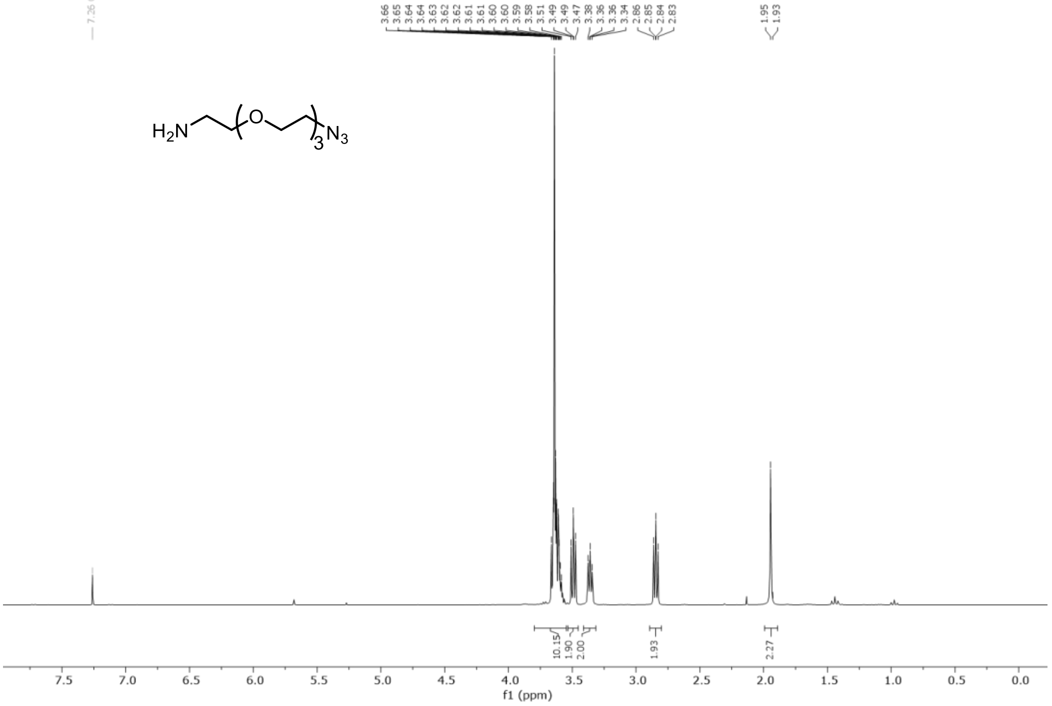

^{1}H-NMR (300 MHz, $CDCl_3$): 11-Amino-1-azido-3,6,9-trioxaundecane (**13**).

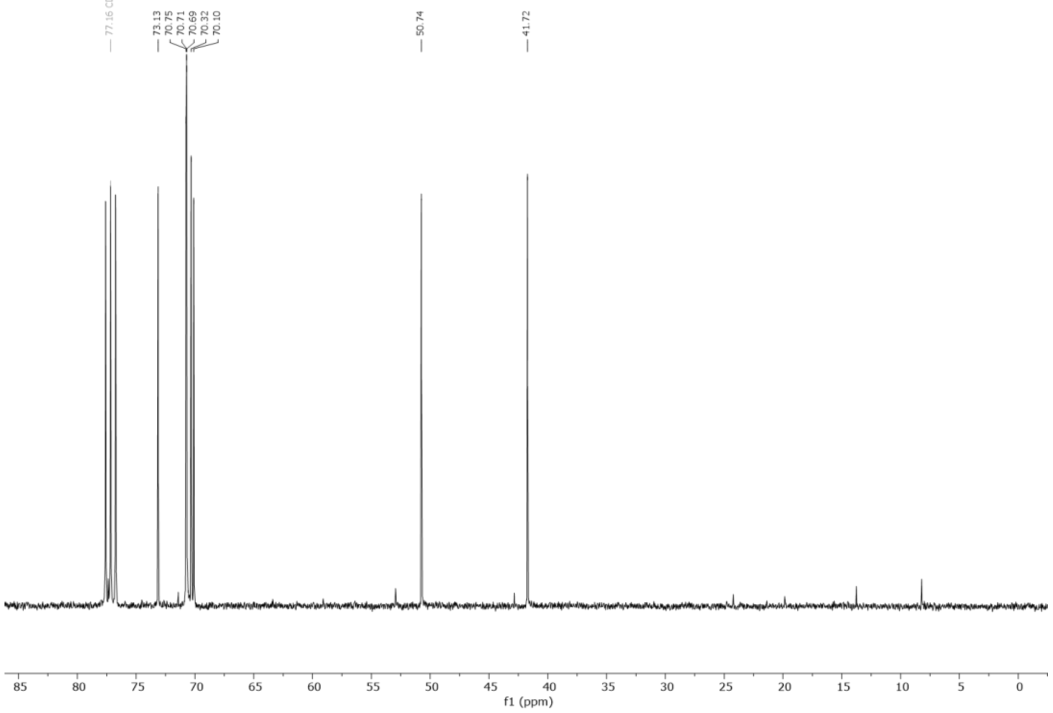

^{13}C-NMR (75 MHz, $CDCl_3$): 11-Amino-1-azido-3,6,9-trioxaundecane (**13**).

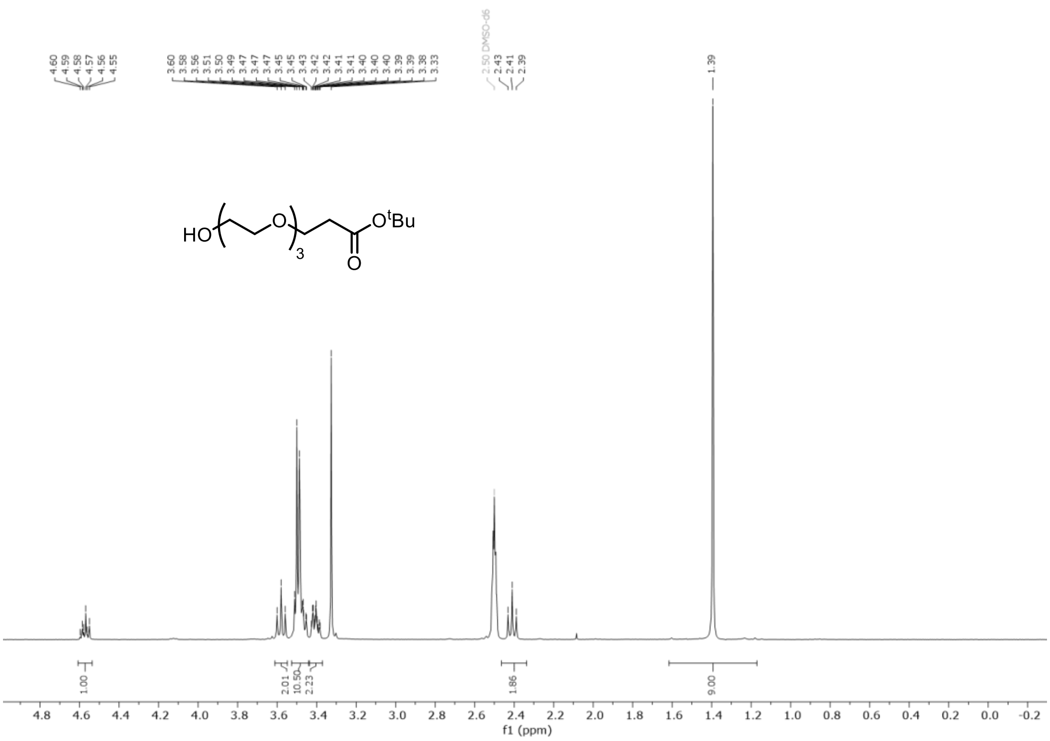

^{1}H-NMR (300 MHz, DMSO-*d*): 12-Hydroxy-4,7,10-trioxadodecanicacid-*tert*-butylester (**S7**).

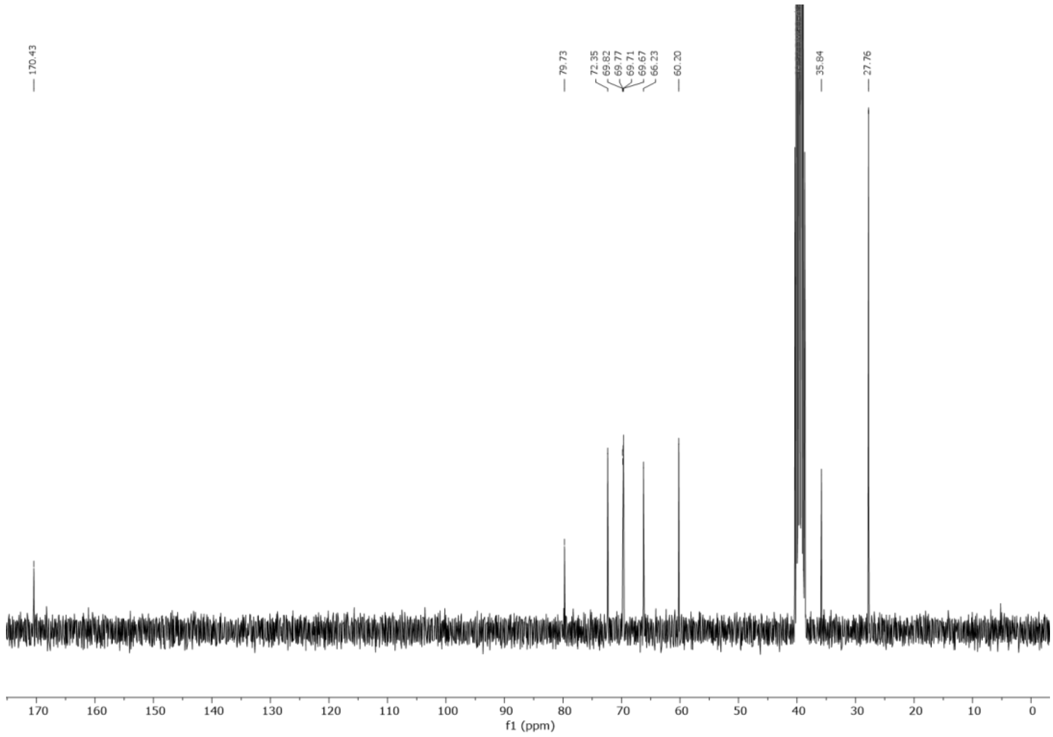

^{13}C-NMR (101 MHz, $CDCl_3$): 12-Hydroxy-4,7,10-trioxadodecanicacid-*tert*-butylester (**S7**).

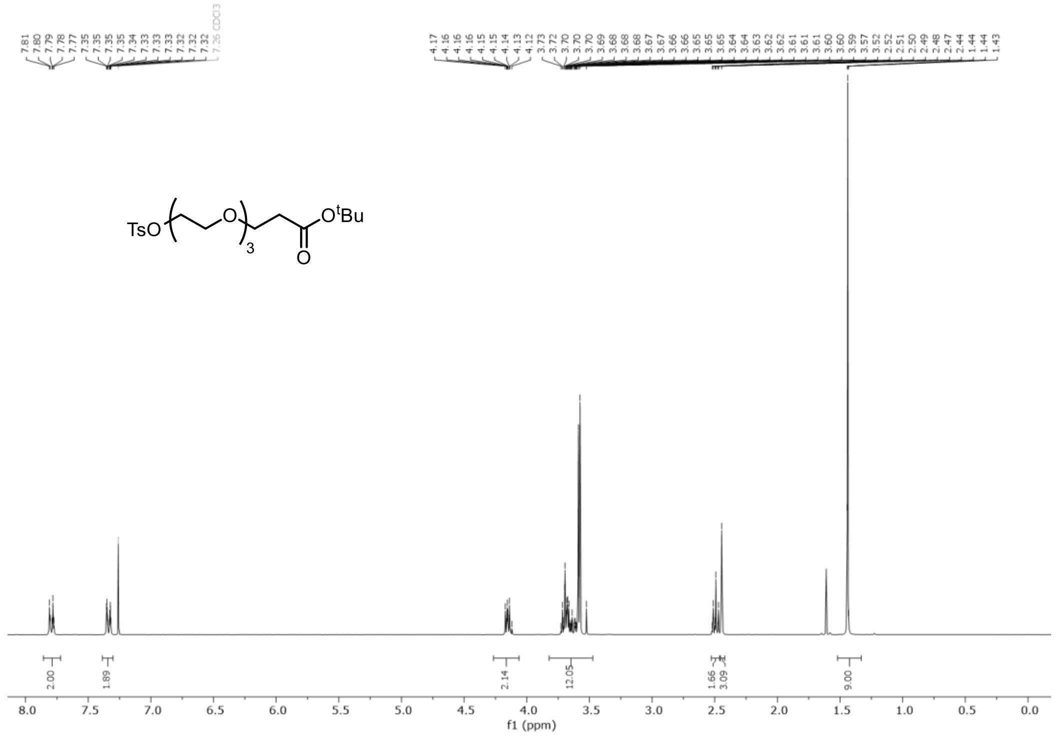

^{1}H-NMR (300 MHz, $CDCl_3$): 12-(*p*-toluenesulfonyl)-4,7,10-trioxadodecanicacid-*tert*-butylester (**S8**).

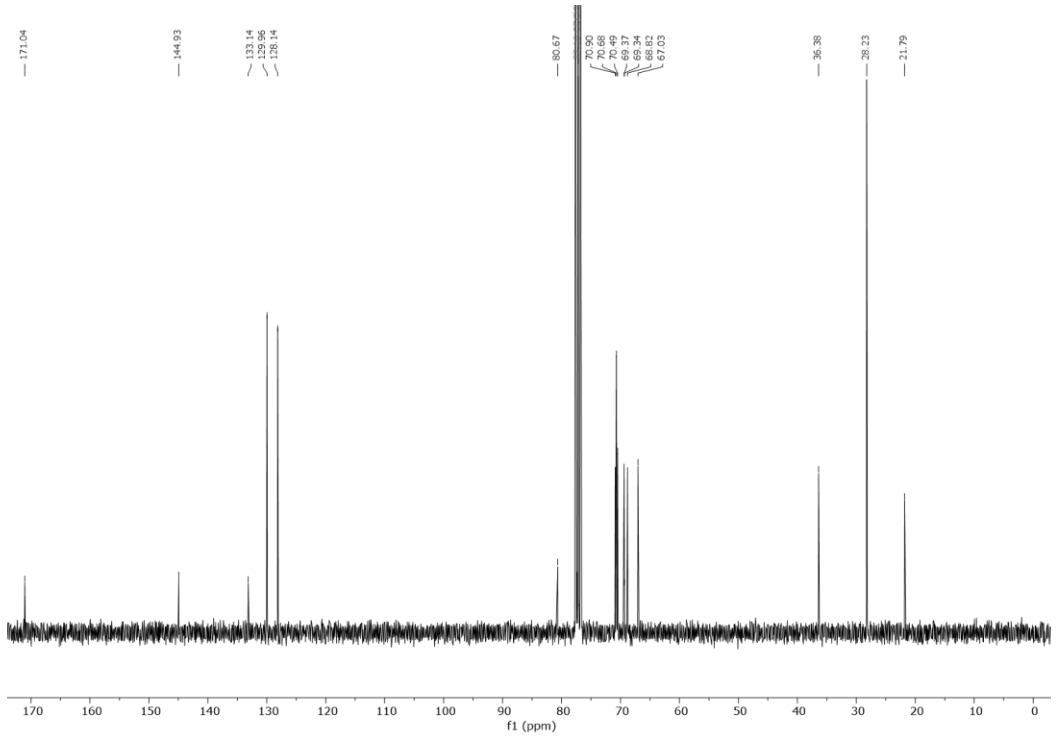

^{13}C-NMR (75 MHz, $CDCl_3$): 12-(*p*-toluenesulfonyl)-4,7,10-trioxadodecanicacid-*tert*-butylester (**S8**).

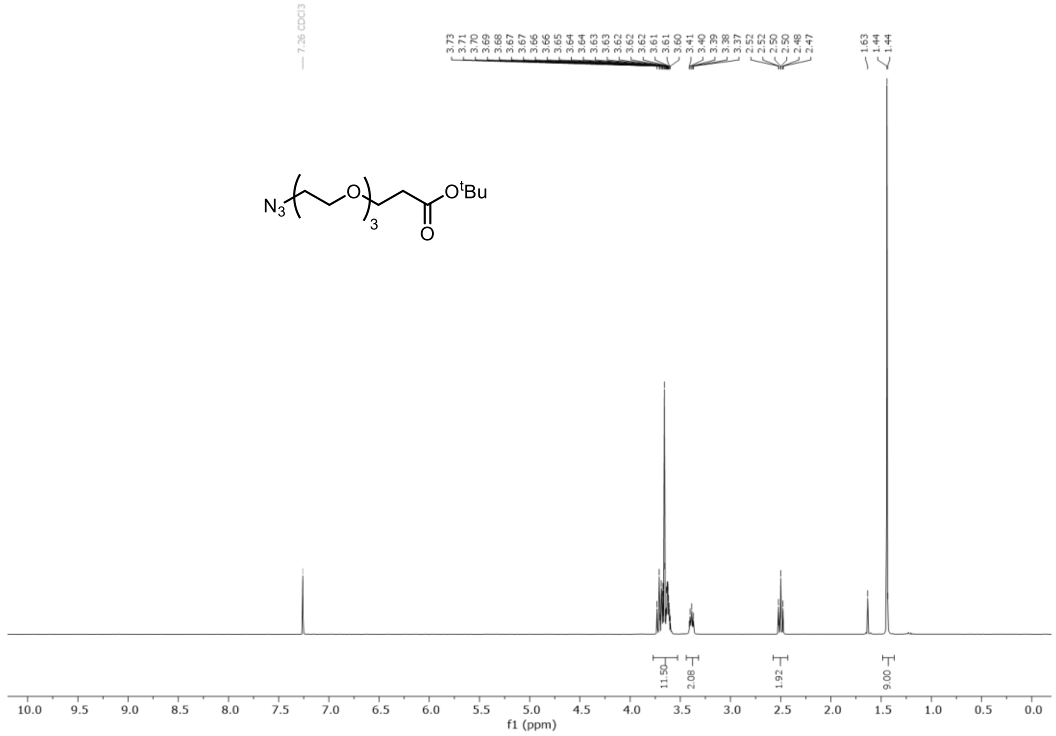

^{1}H-NMR (300 MHz, $CDCl_3$): 12-Azido-4,7,10-trioxadodecanic acid tert-butyl ester (**S9**).

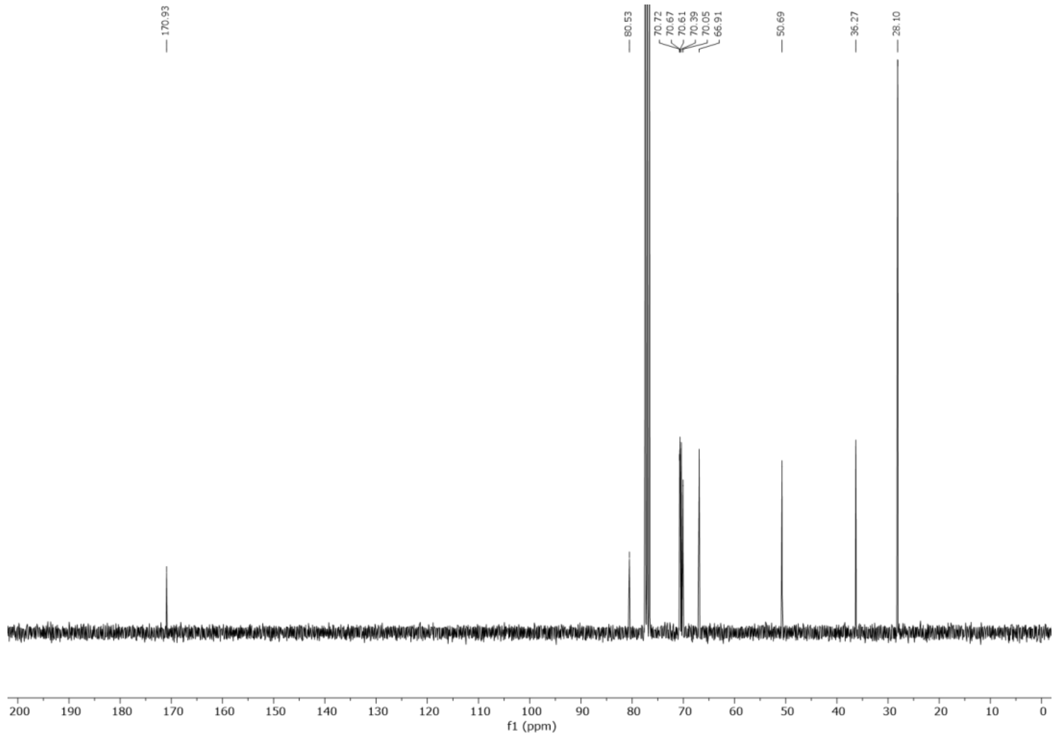

^{13}C-NMR (75 MHz, $CDCl_3$): 12-Azido-4,7,10-trioxadodecanic acid tert-butyl ester (**S9**).

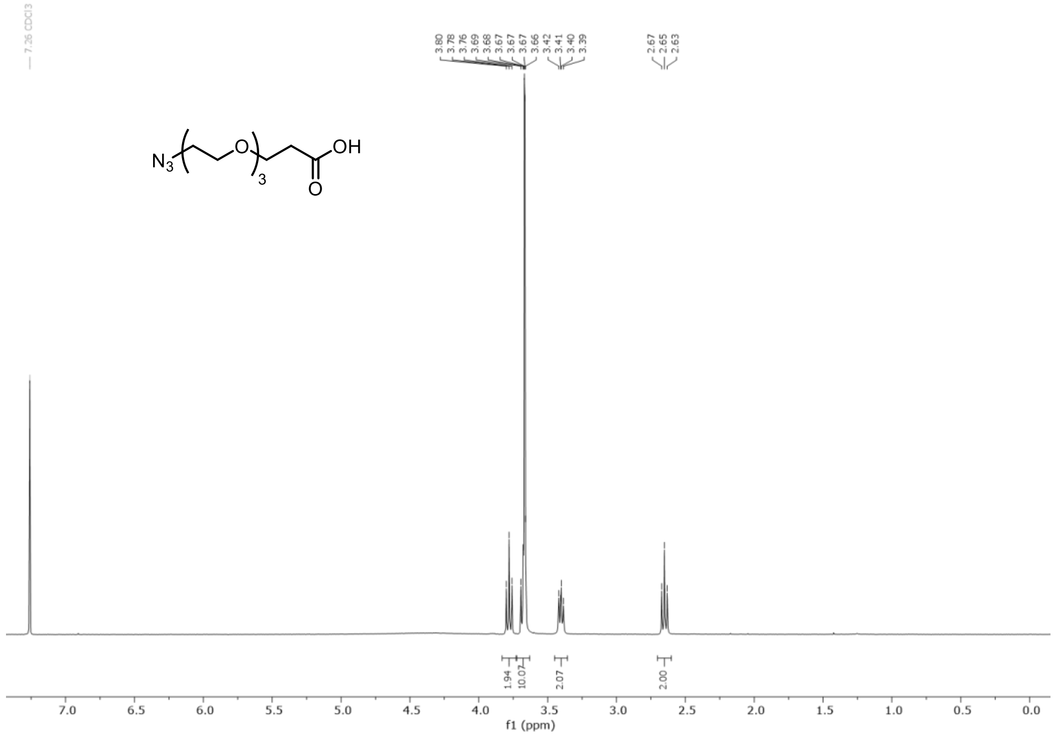

^{1}H-NMR (300 MHz, $CDCl_3$): 12-Azido-4,7,10-trioxadodecanic acid (**16**).

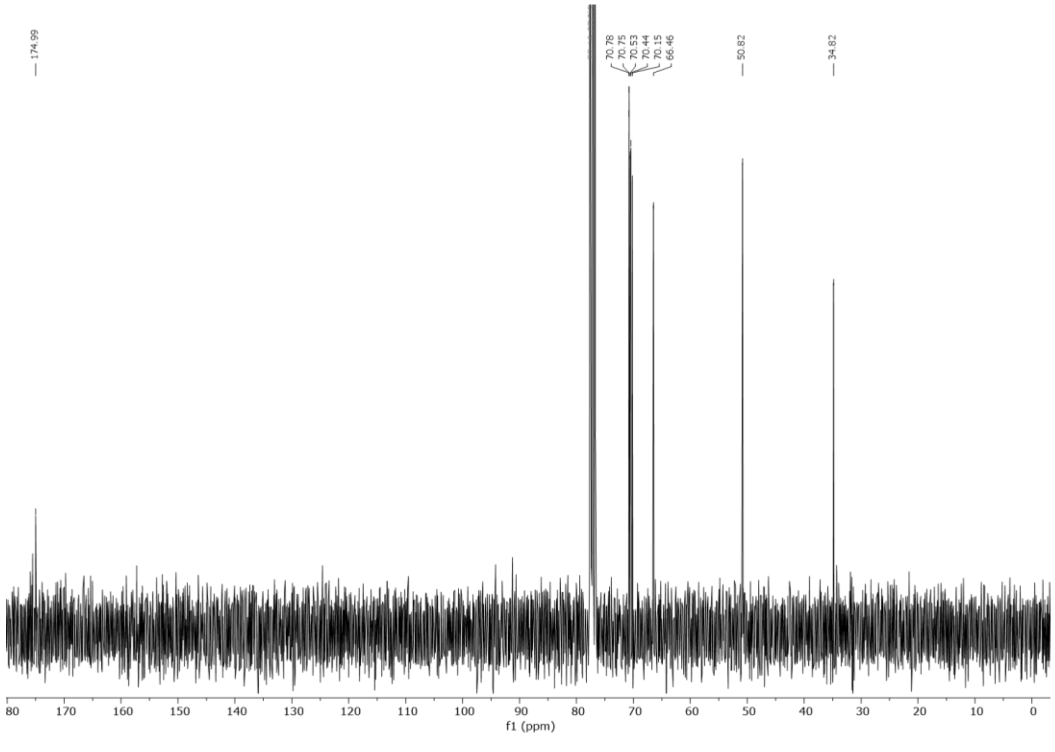

^{13}C-NMR (75 MHz, $CDCl_3$): 12-Azido-4,7,10-trioxadodecanic acid (**16**).

IV.X NMR spectra for the clickable fucose-*N*-acetyl-glucosamine-disaccharide

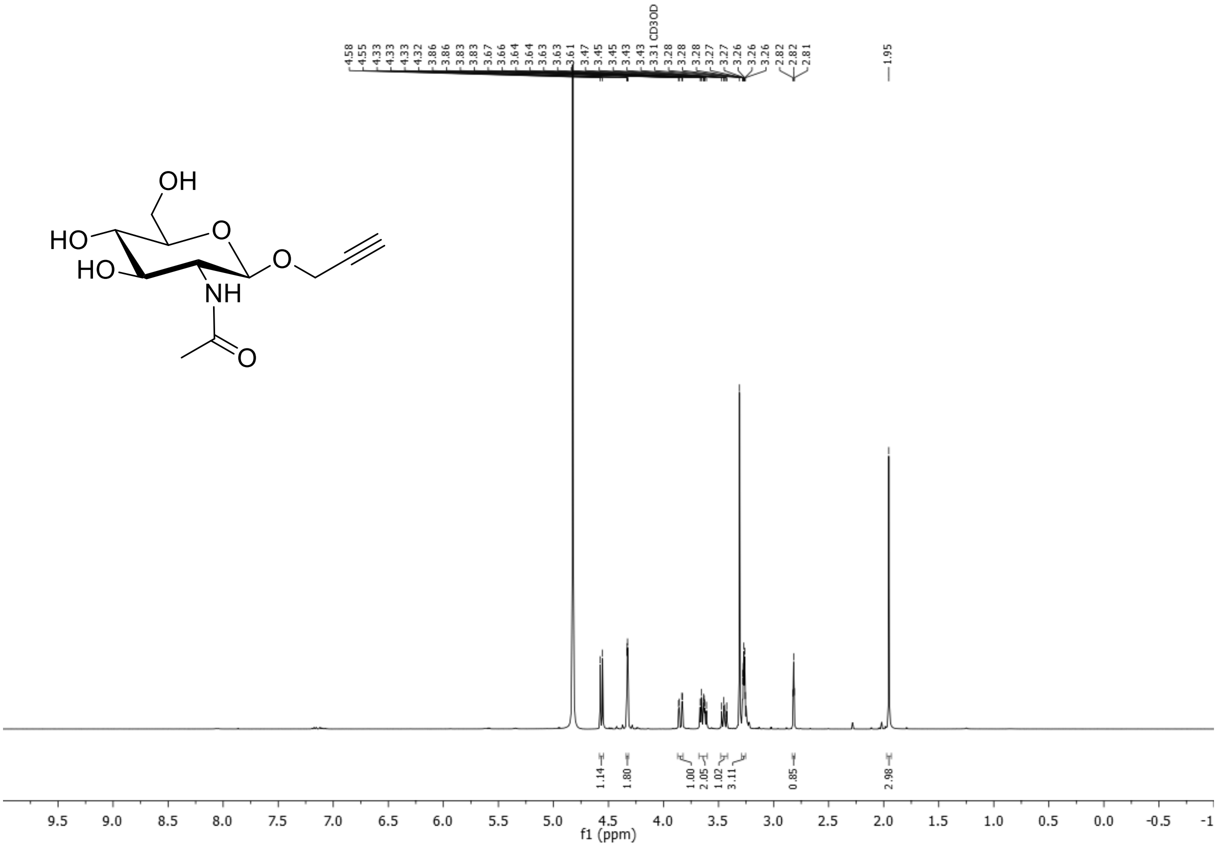

^{1}H-NMR (300 MHz, $CDCl_3$): Propargyl 2-acetamido-2-deoxy-β-D-glucopyranoside (**S14**).

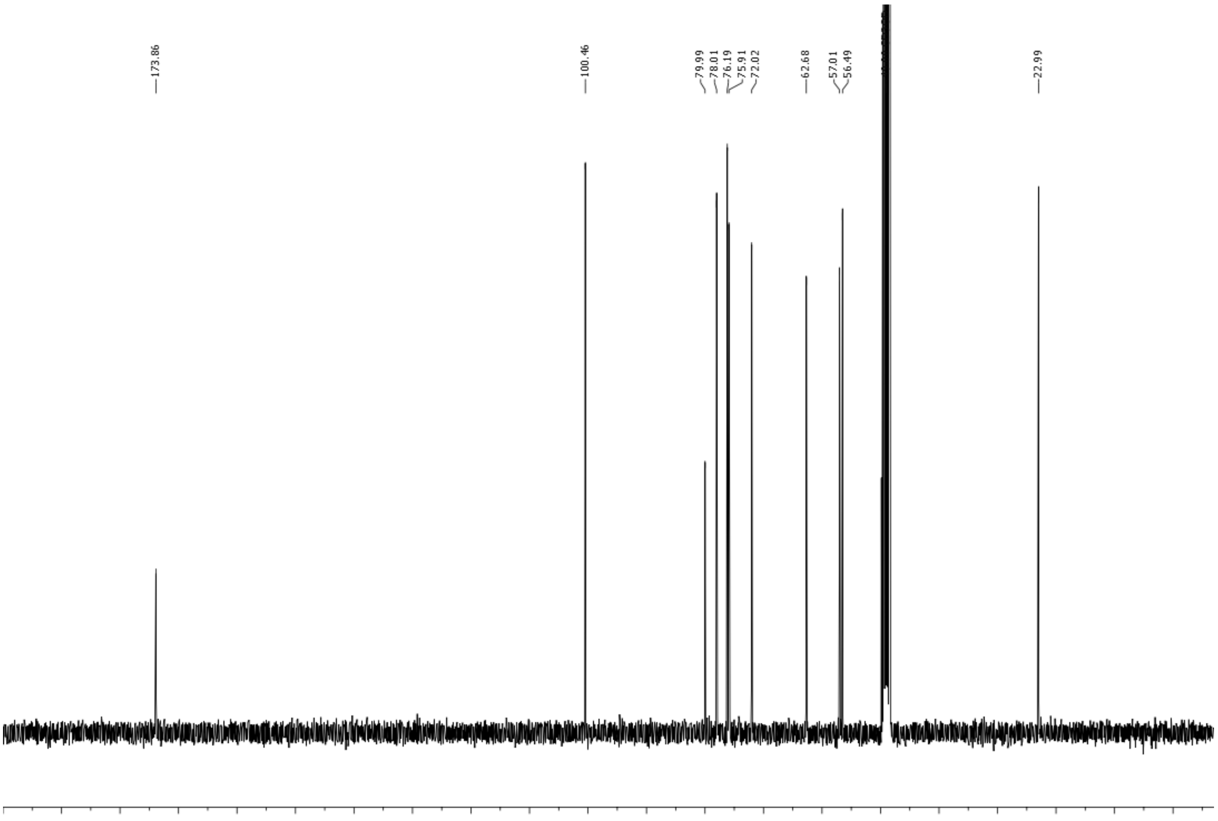

^{13}C-NMR (75.5 MHz, $CDCl_3$): Propargyl 2-acetamido-2-deoxy-β-D-glucopyranoside (**S14**).

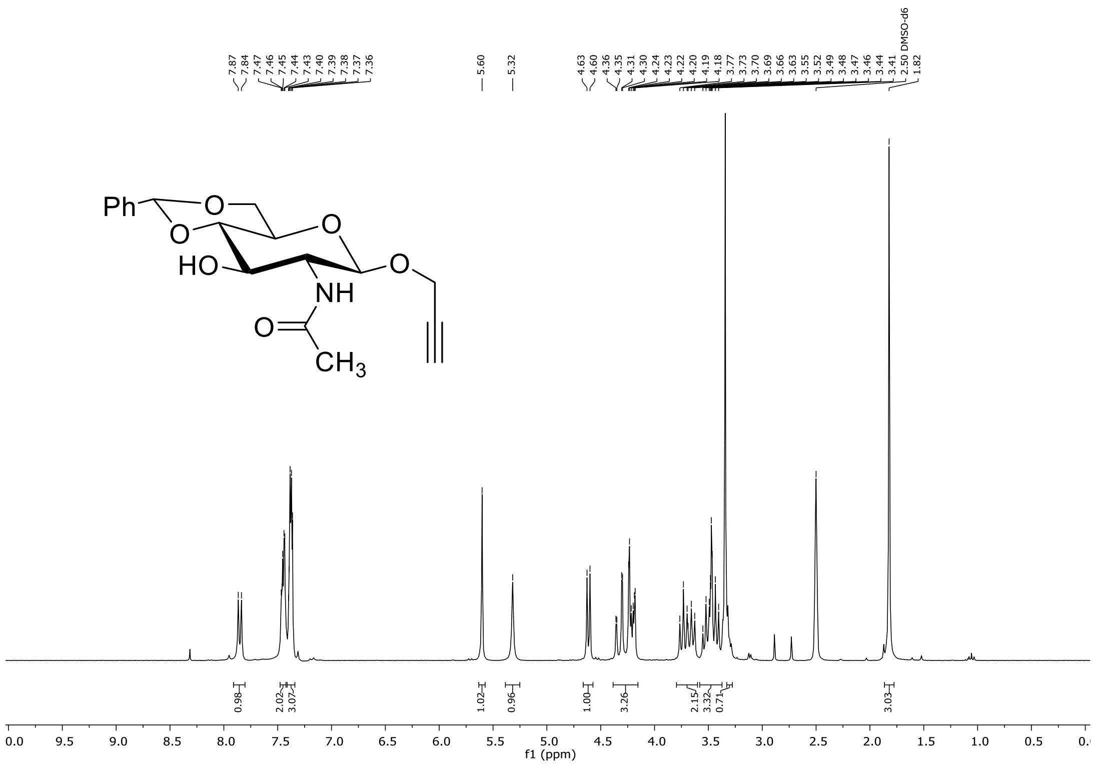

^{1}H-NMR (300 MHz, DMSO-d_6): Propargyl 2-acetamido-2-deoxy-4,6-*O*-benzylidene-β-D-glucopyranoside (**6**).

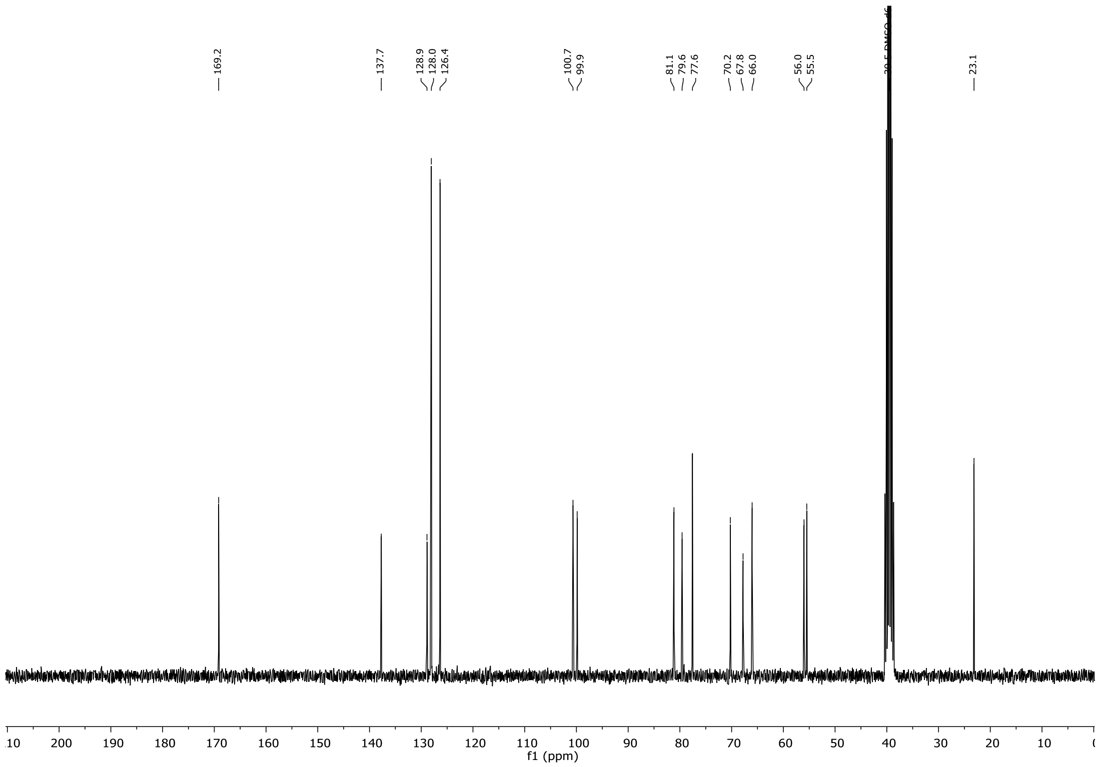

^{13}C-NMR (75.5 MHz, DMSO-d_6): Propargyl 2-acetamido-2-deoxy-4,6-*O*-benzylidene-β-D-glucopyranoside (**6**).

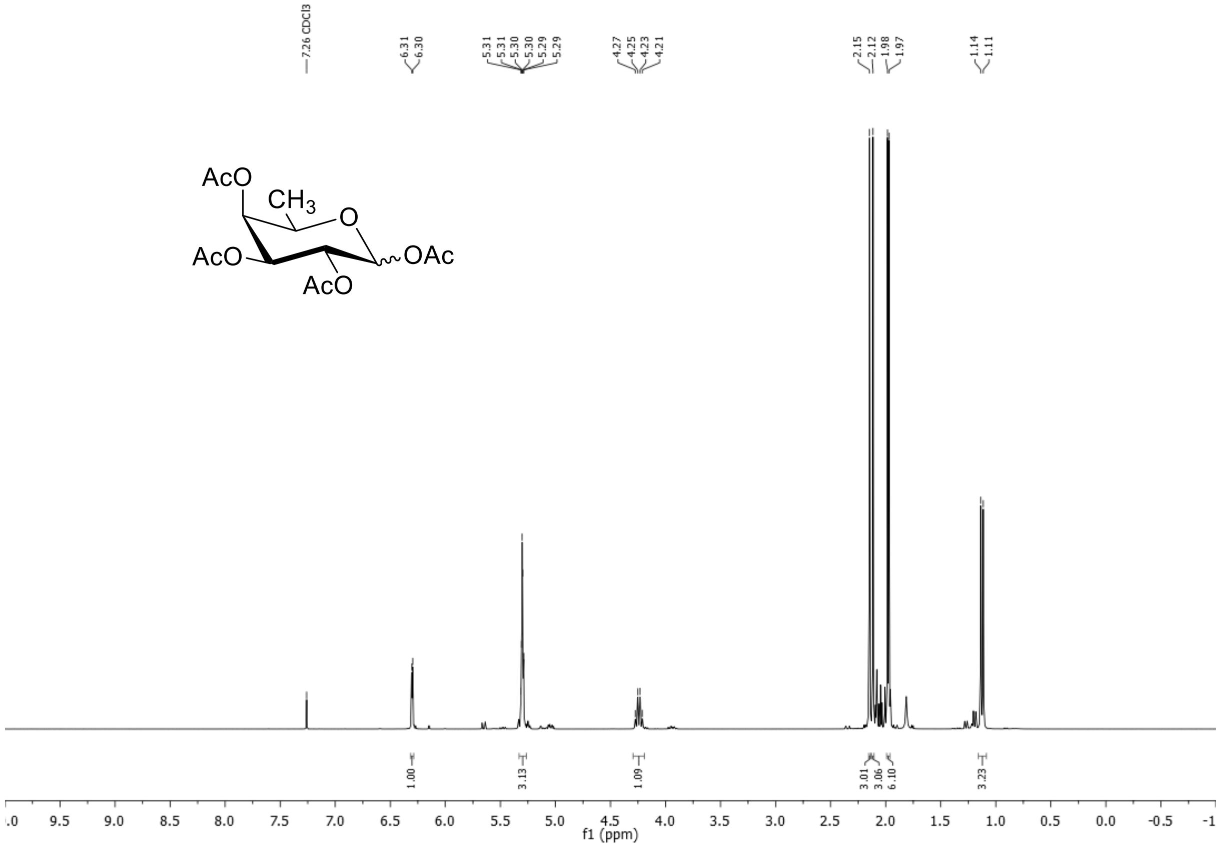

^{1}H-NMR (300 MHz, $CDCl_3$): 1,2,3,4-Tetra-*O*-acetyl-α-L-fucopyranoside (**S15**).

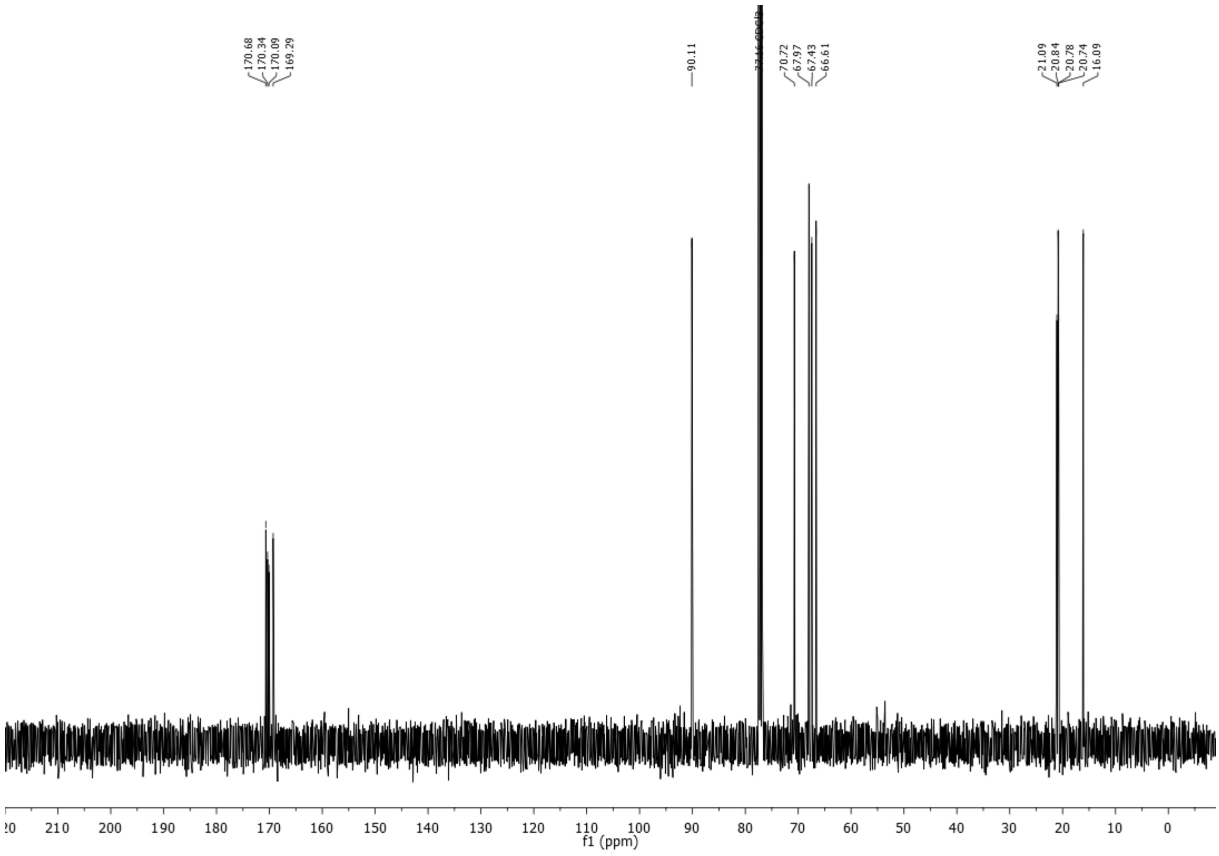

^{13}C-NMR (75.5 MHz, $CDCl_3$): 1,2,3,4-Tetra-*O*-acetyl-α-L-fucopyranoside (**S15**).

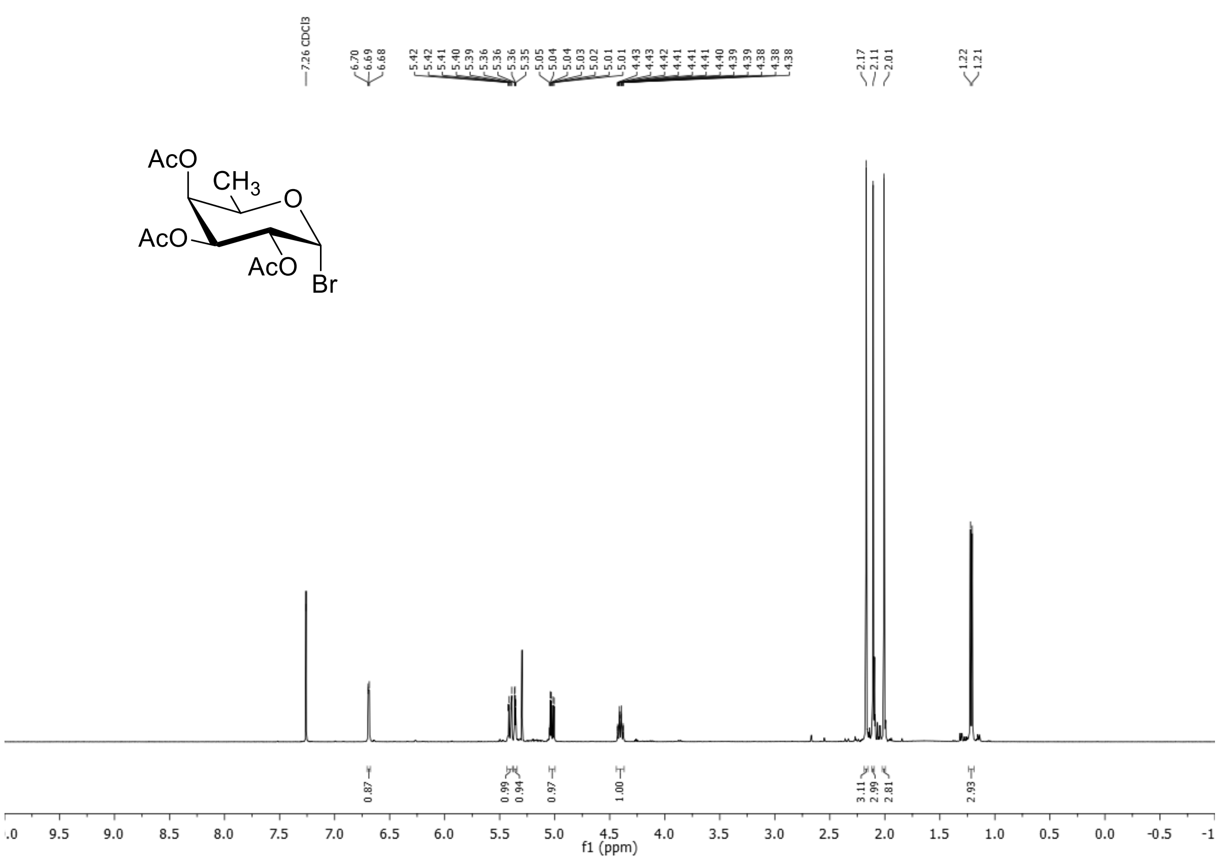

^{1}H-NMR (300 MHz, $CDCl_3$): 1,2,3-Tri-*O*-acetyl-α-L-fucopyranosyl bromide (**8**).

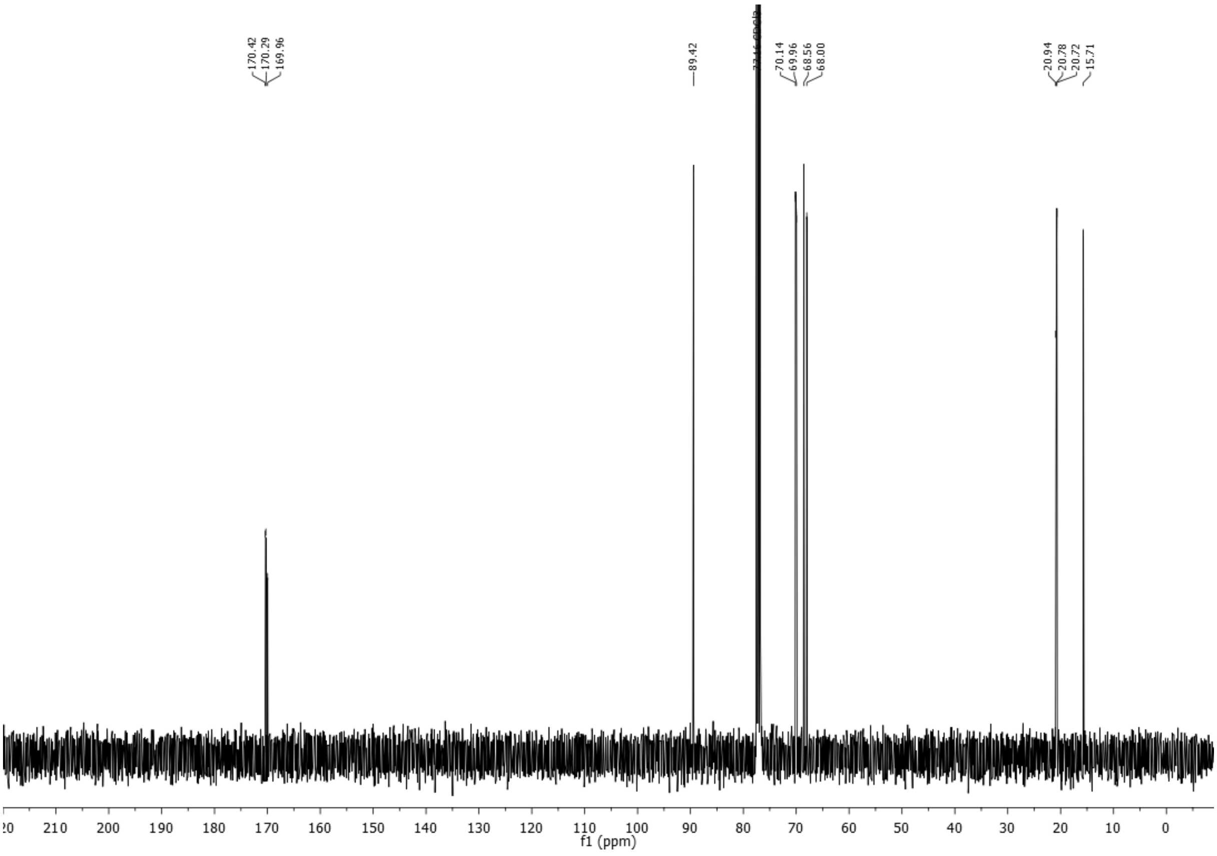

^{13}C-NMR (75.5 MHz, $CDCl_3$): 1,2,3-Tri-*O*-acetyl-α-L-fucopyranosyl bromide (**8**).

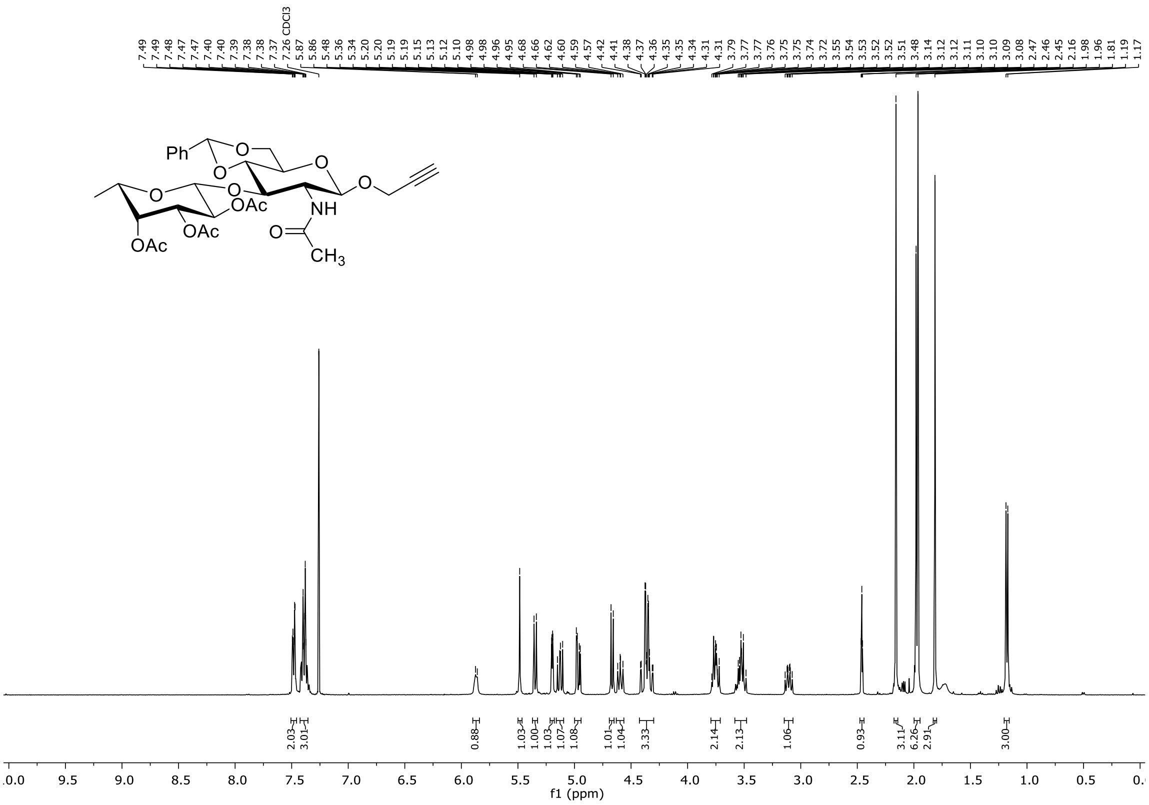

^{1}H-NMR (400 MHz, $CDCl_3$): Propargyl (2,3,4-tri-*O*-acetyl-β-L-fucopyranosyl)-(1→3)-2-acetamido-2-deoxy-4,6-*O*-benzylidene-β-D-glucopyranoside (**S16**).

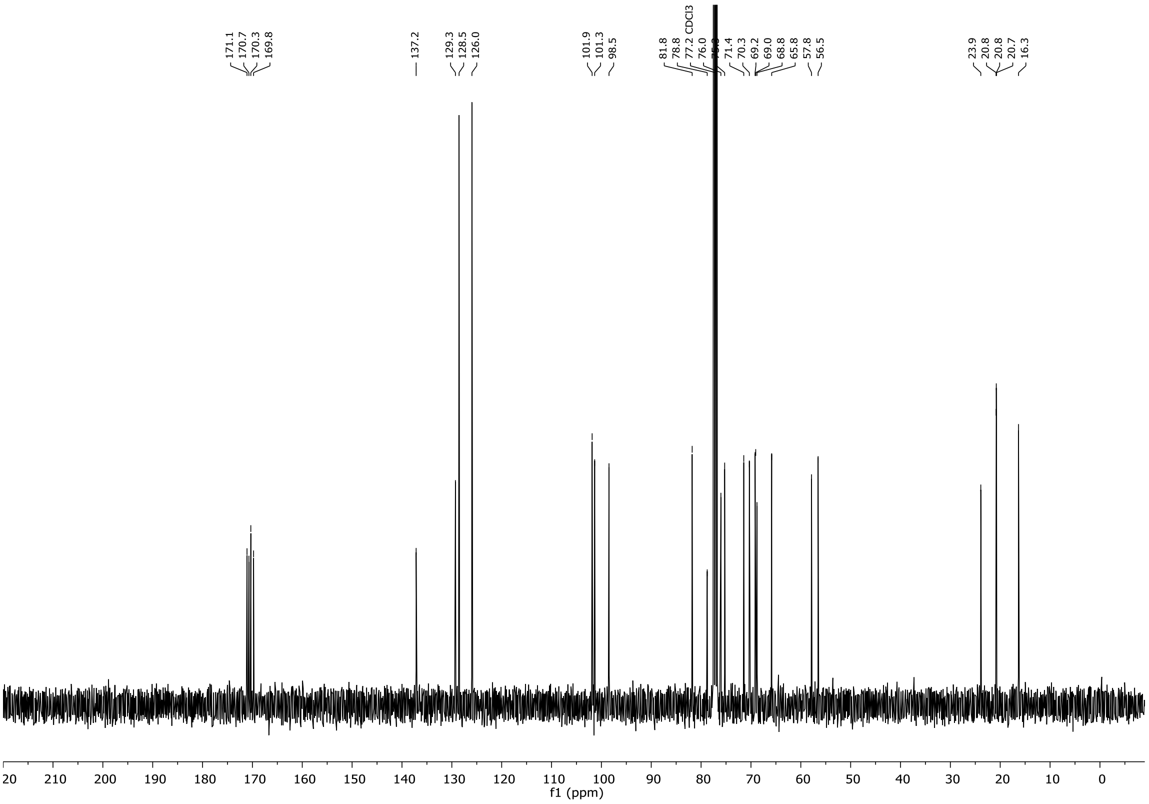

^{13}C-NMR (100.6 MHz, $CDCl_3$): Propargyl (2,3,4-tri-*O*-acetyl-β-L-fucopyranosyl)-(1→3)-2-acetamido-2-deoxy-4,6-*O*-benzylidene-β-D-glucopyranoside (**S16**).

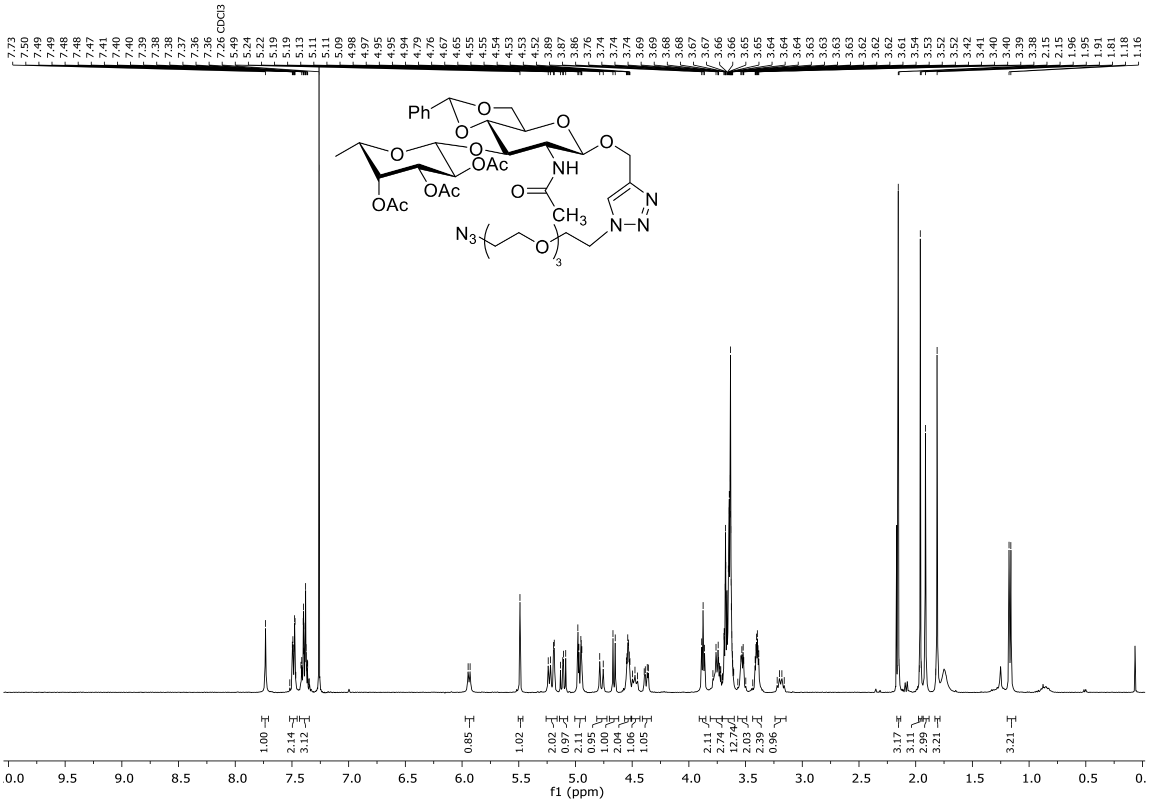

^{1}H-NMR (400 MHz, $CDCl_3$): (1-(2-(2-(2-(2-Azidoethoxy)ethoxy)ethoxy)ethyl)-1*H*-1,2,3-triazol-4-yl)methyl-(2,3,4-tri-*O*-acetyl-β-L-fucopyranosyl)-(1→3)-2-acetamido-2-deoxy-4,6-*O*-benzylidene-β-D-glucopyranoside (**S17**).

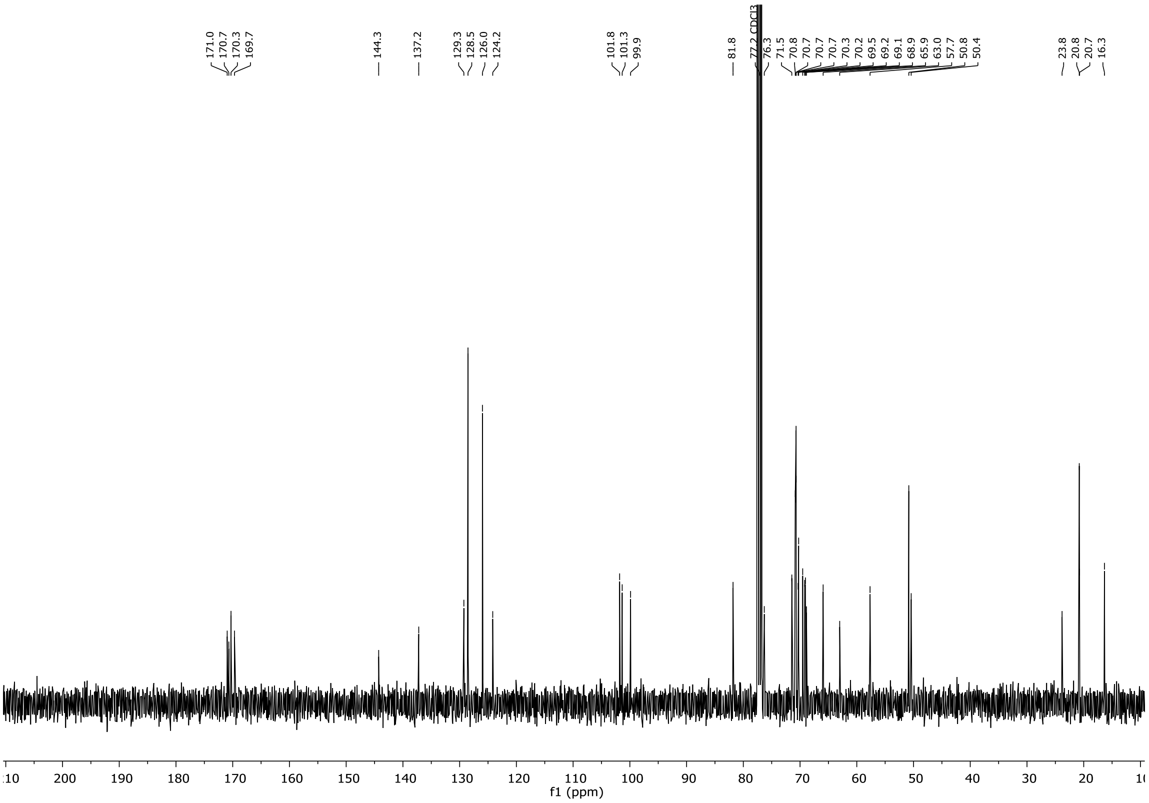

^{13}C-NMR (100.6 MHz, $CDCl_3$): (1-(2-(2-(2-(2-Azidoethoxy)ethoxy)ethoxy)ethyl)-1*H*-1,2,3-triazol-4-yl)methyl-(2,3,4-tri-*O*-acetyl-β-L-fucopyranosyl)-(1→3)-2-acetamido-2-deoxy-4,6-*O*-benzylidene-β-D-glucopyranoside (**S17**).

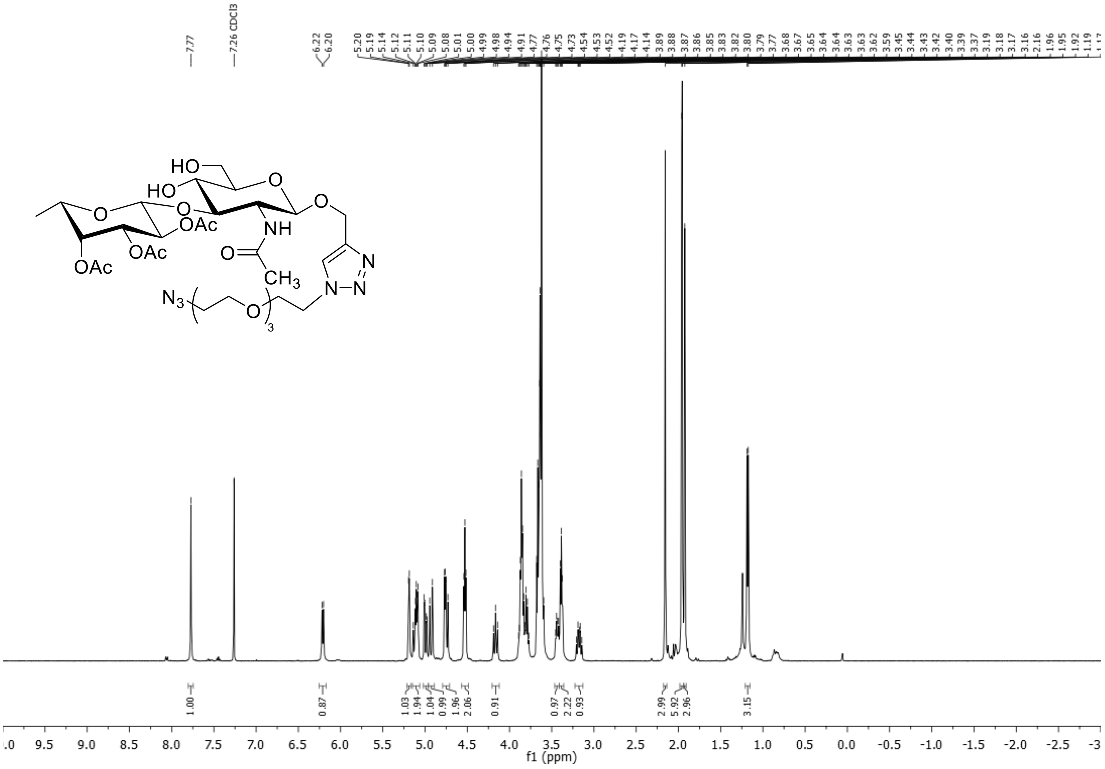

^{1}H-NMR (600 MHz, CD_3OD): (1-(2-(2-(2-(2-Azidoethoxy)ethoxy)ethoxy)ethyl)-1*H*-1,2,3-triazol-4-yl)methyl-(2,3,4-tri-*O*-acetyl-β-L-fucopyranosyl)-(1→3)-2-acetamido-2-deoxy-β-D-glucopyranoside (**S18**).

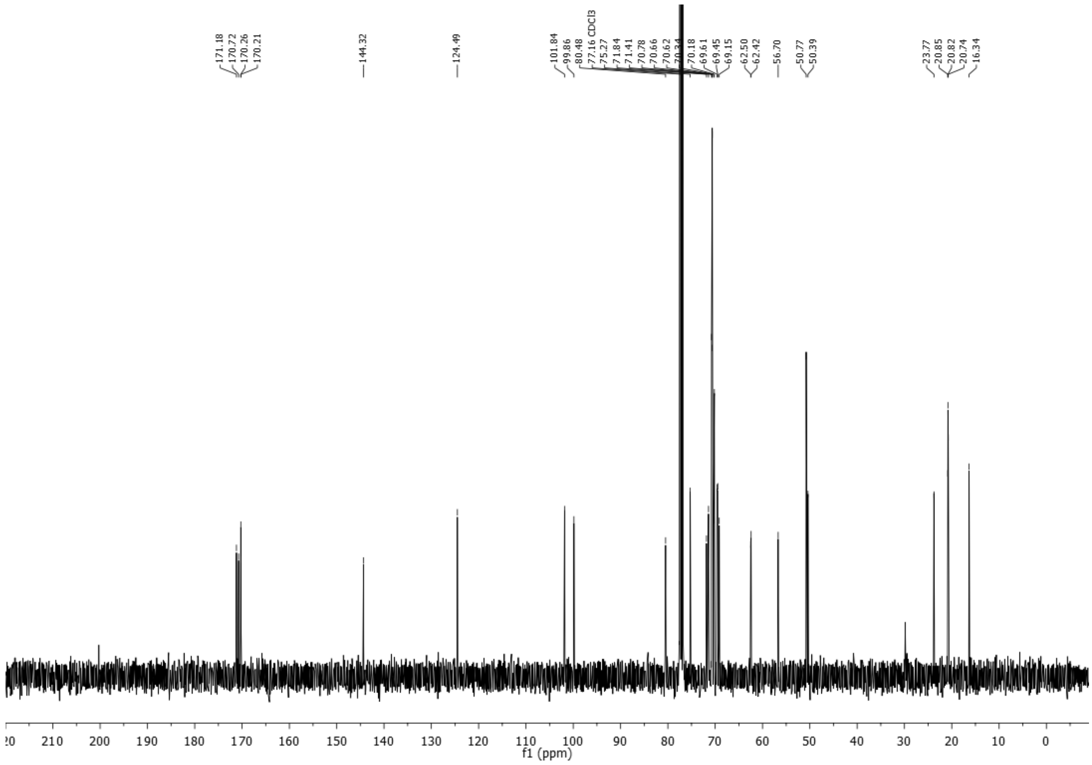

^{13}C-NMR (151 MHz, CD_3OD): (1-(2-(2-(2-(2-Azidoethoxy)ethoxy)ethoxy)ethyl)-1*H*-1,2,3-triazol-4-yl)methyl-(2,3,4-tri-*O*-acetyl-β-L-fucopyranosyl)-(1→3)-2-acetamido-2-deoxy-β-D-glucopyranoside (**S18**).

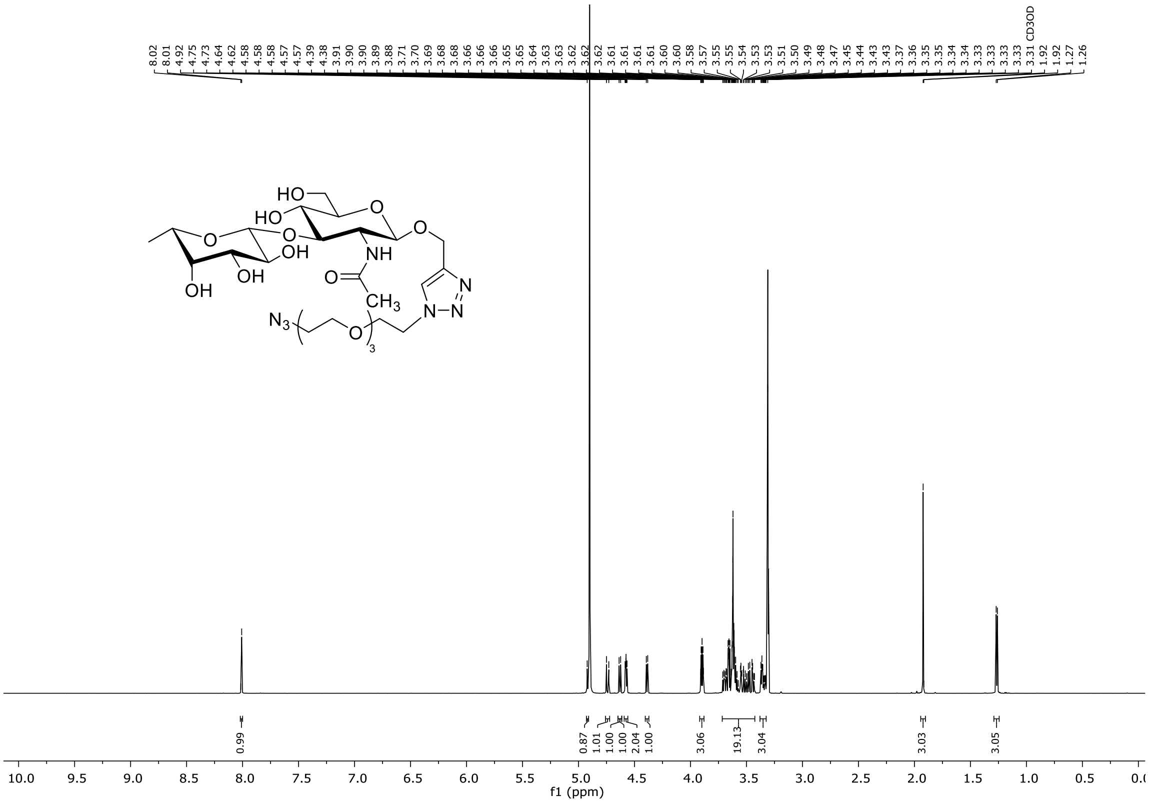

^{1}H-NMR (600 MHz, CD_3OD): (1-(2-(2-(2-(2-Azidoethoxy)ethoxy)ethoxy)ethyl)-1*H*-1,2,3-triazol-4-yl)methyl-(β-L-fucopyranosyl)-(1→3)-2-acetamido-2-deoxy-β-D-glucopyranoside (**3**).

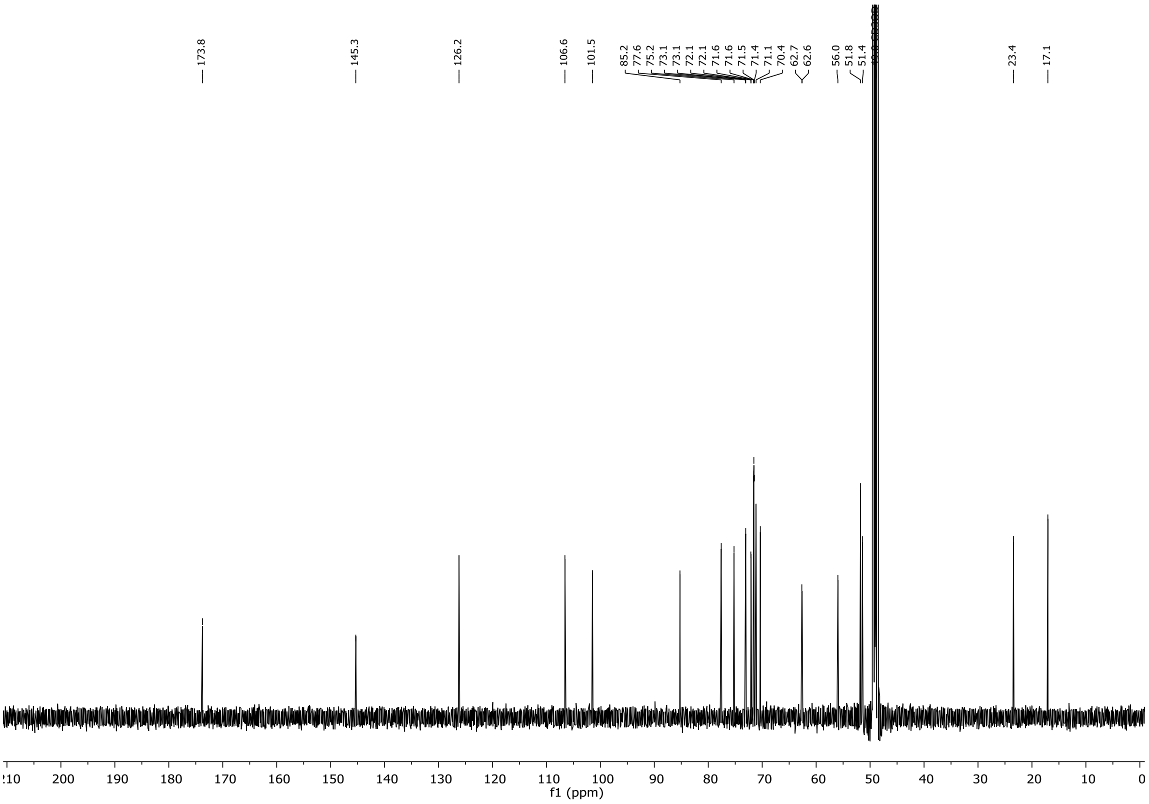

^{13}C-NMR (151 MHz, CD_3OD): (1-(2-(2-(2-(2-Azidoethoxy)ethoxy)ethoxy)ethyl)-1*H*-1,2,3-triazol-4-yl)methyl-(β-L-fucopyranosyl)-(1→3)-2-acetamido-2-deoxy-β-D-glucopyranoside (**3**).

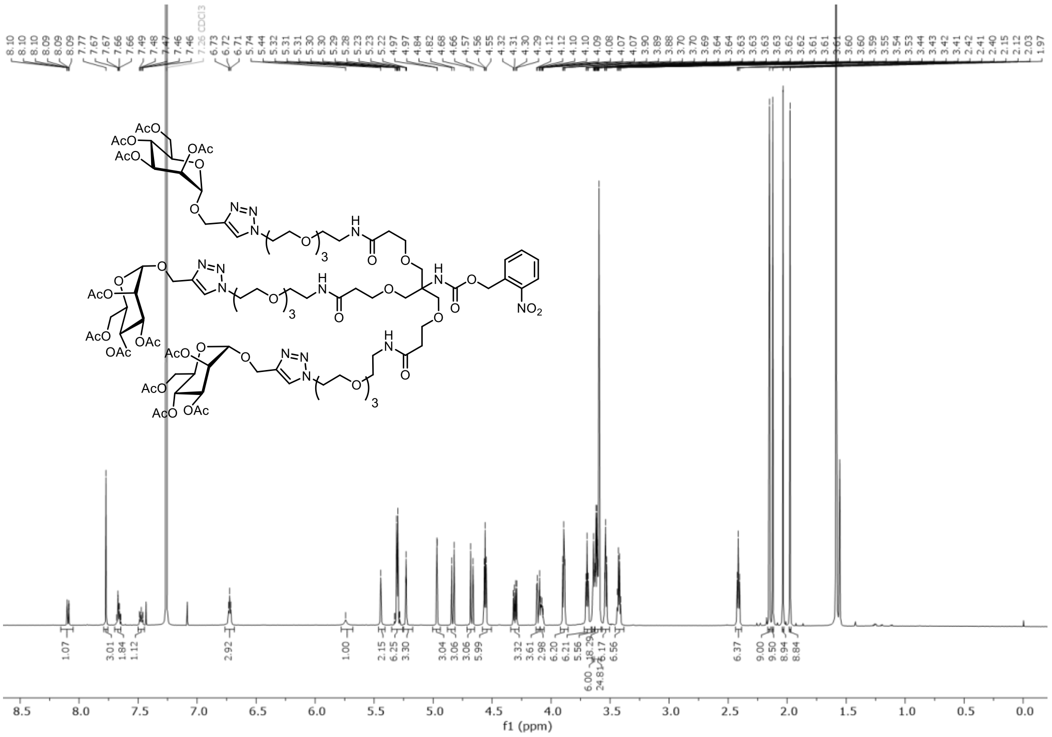

^{1}H-NMR (600 MHz, $CDCl_3$): *o*-Nitrobenzyl (1,3,5-bis((4-(2,3,4,6-tetra-*O*-acetyl-α-D-mannopyranosyl)-methyl)-1H-1,2,3-triazol-1-yl)-18-(17-((4-(2,3,4,6-tetra-*O*-acetyl-α-D-mannopyranosyl))-methyl)-5-oxo-2,9,12,15-tetraoxo-6-azaheptadec-1-yl)-13,23-dioxo-3,6,9,16,20,27,30,33-octaoxa-12,24-diazapentatriacontan-18-yl)-carbamate (**S25**).

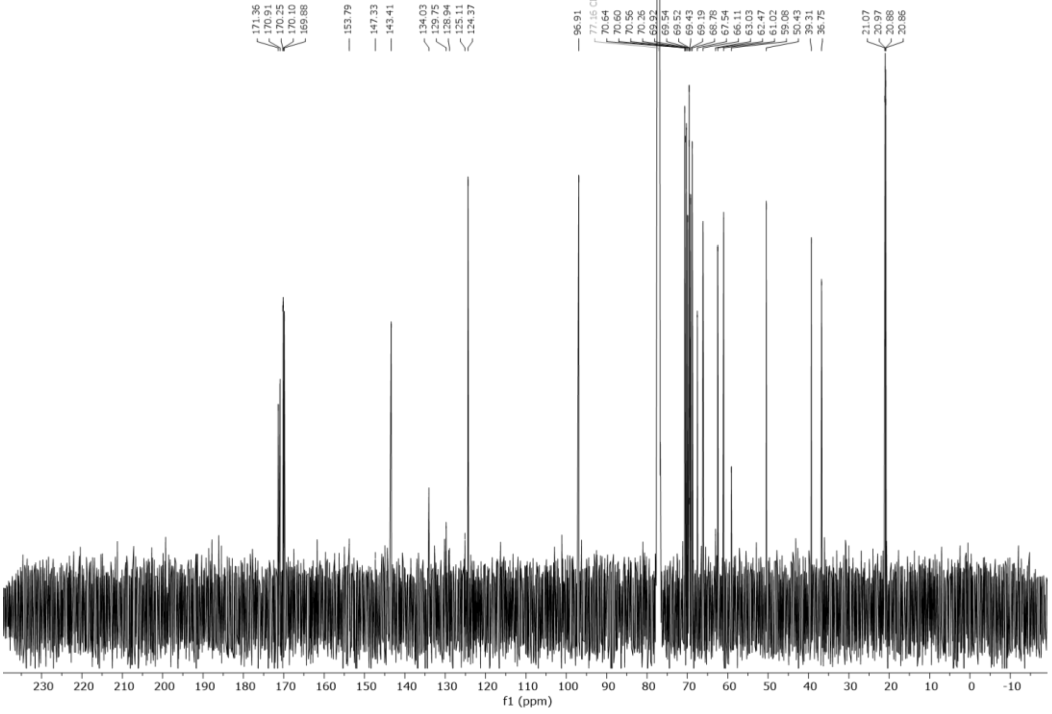

^{13}C-NMR (151 MHz, $CDCl_3$): *o*-Nitrobenzyl (1,3,5-bis((4-(2,3,4,6-tetra-*O*-acetyl-α-D-mannopyranosyl)-methyl)-1H-1,2,3-triazol-1-yl)-18-(17-((4-(2,3,4,6-tetra-*O*-acetyl-α-D-mannopyranosyl))-methyl)-5-oxo-2,9,12,15-tetraoxo-6-azaheptadec-1-yl)-13,23-dioxo-3,6,9,16,20,27,30,33-octaoxa-12,24-diazapentatriacontan-18-yl)-carbamate (**S25**).

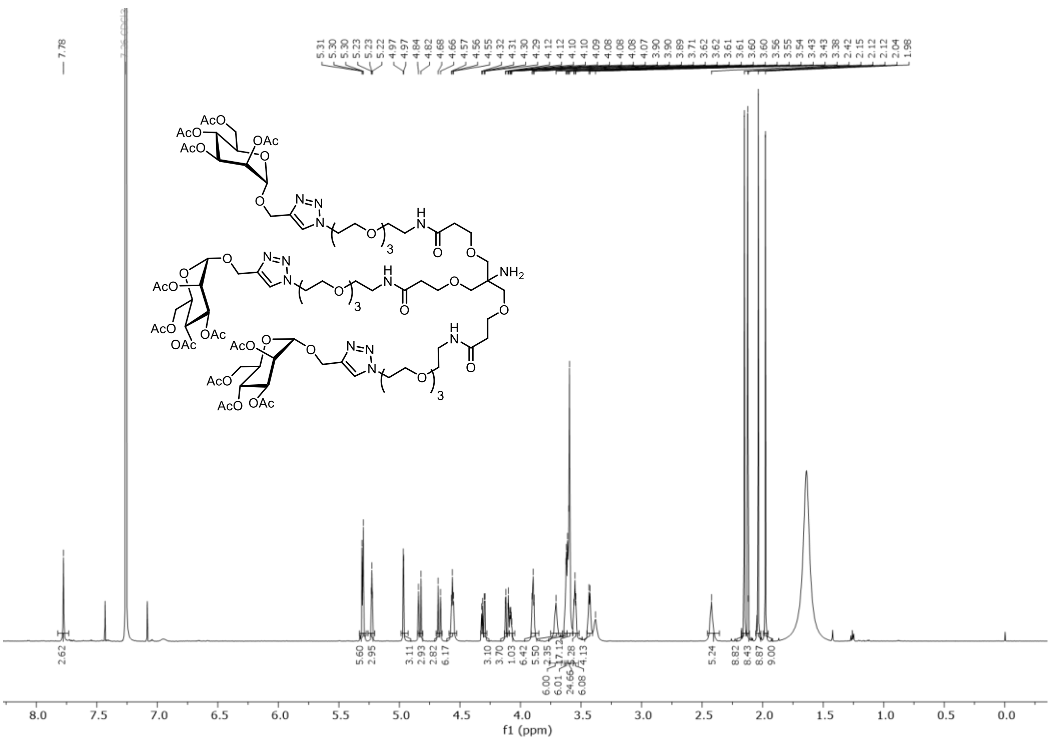

^{1}H-NMR (600 MHz, $CDCl_3$): *N*-(1-((4-(2,3,4,6-tetra-*O*-acetyl-α-D-mannopyranosyl)-methyl)-1*H*-1,2,3-triazol-1-yl)-3,6,9-trioxa-undec-11-yl)-6-amino-6-(17-((4-(2,3,4,6-tetra-*O*-acetyl-α-D-mannopyranosyl)-methyl)-1*H*-1,2,3-triazol-1-yl)-5-oxo-2,9,12,15-tetraoxa-6-azaheptadec-1-yl)-11-oxo-4,8,15,18,21-pentaoxa-12-azatricosanamide (**S26**).

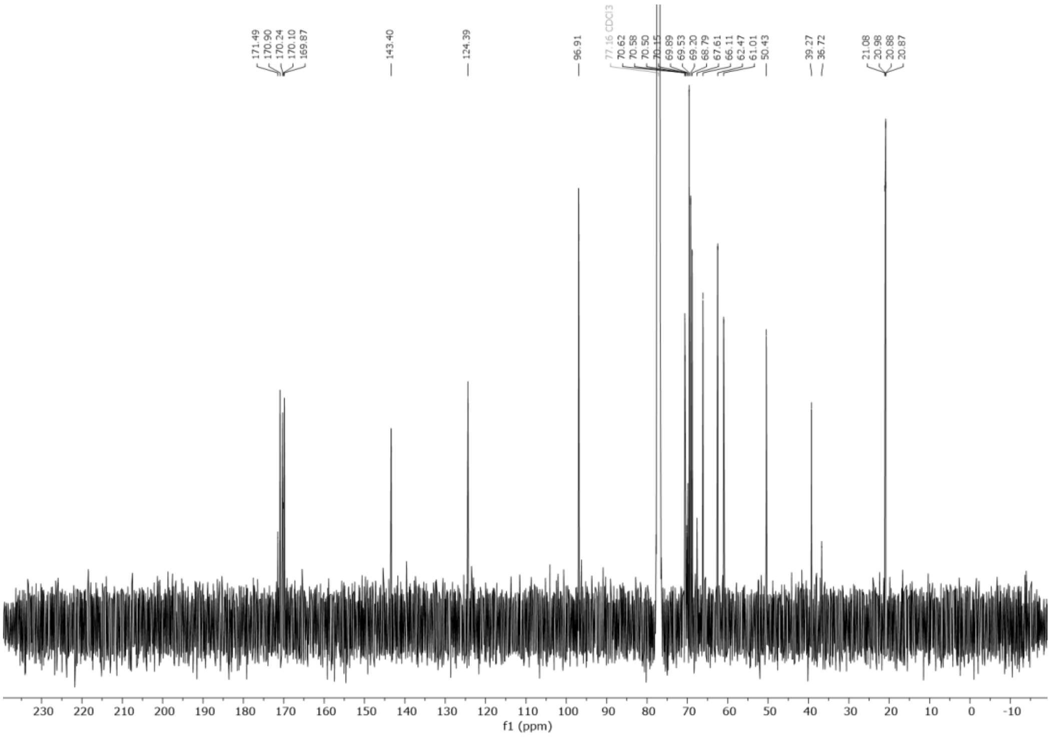

^{13}C-NMR (151 MHz, $CDCl_3$): *N*-(1-((4-(2,3,4,6-tetra-*O*-acetyl-α-D-mannopyranosyl)-methyl)-1*H*-1,2,3-triazol-1-yl)-3,6,9-trioxa-undec-11-yl)-6-amino-6-(17-((4-(2,3,4,6-tetra-*O*-acetyl-α-D-mannopyranosyl)-methyl)-1*H*-1,2,3-triazol-1-yl)-5-oxo-2,9,12,15-tetraoxa-6-azaheptadec-1-yl)-11-oxo-4,8,15,18,21-pentaoxa-12-azatricosanamide (**S26**).

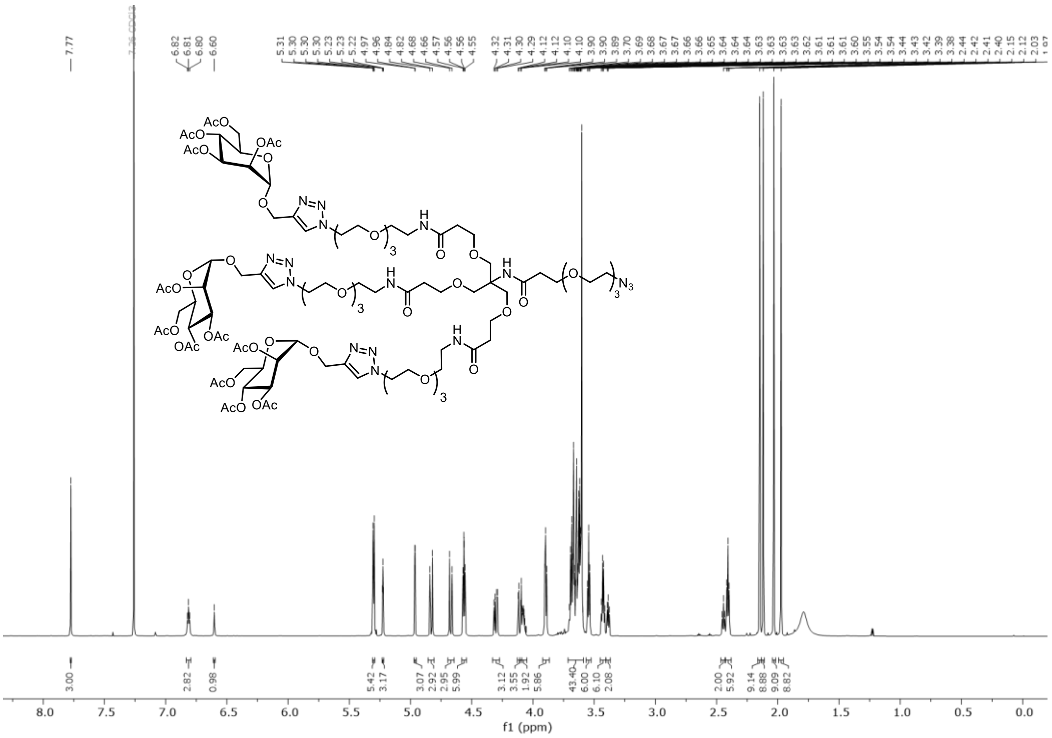

^{1}H-NMR (600 MHz, $CDCl_3$): *N*-(1-bis(17-((4-(2,3,4,6-tetra-*O*-acetyl-α-D-mannopyranosyl)-methyl)-1*H*-1,2,3-triazol-1-yl)-5-oxo-2,9,12,15-tetraoxa-6-azaheptadec-1-yl)-18-((4-(2,3,4,6-tetra-*O*-acetyl-α-D-mannopyranosyl)-methyl)-1*H*-1,2,3-triazol-1-yl)-6-oxo-3,10,13,16-tetraoxa-7-azaoctadec-1-yl)-12-azido-4,7,10-pentaoxadodecanamide (**S27**).

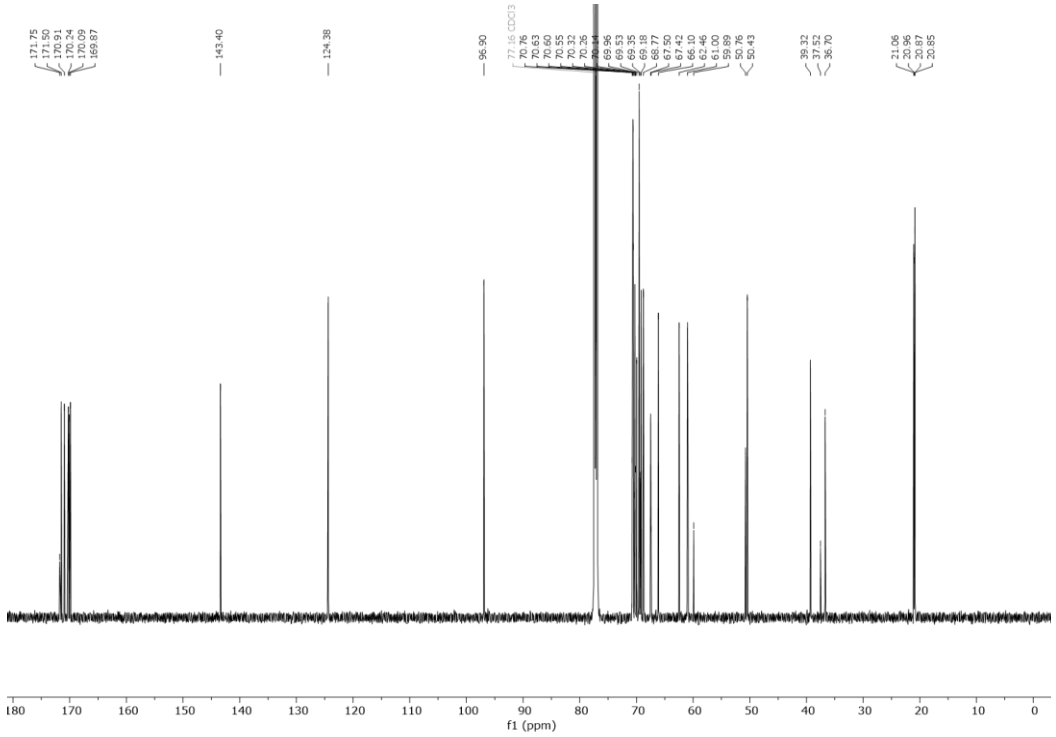

^{13}C-NMR (151 MHz, $CDCl_3$): *N*-(1-bis(17-((4-(2,3,4,6-tetra-*O*-acetyl-α-D-mannopyranosyl)-methyl)-1*H*-1,2,3-triazol-1-yl)-5-oxo-2,9,12,15-tetraoxa-6-azaheptadec-1-yl)-18-((4-(2,3,4,6-tetra-*O*-acetyl-α-D-mannopyranosyl)-methyl)-1*H*-1,2,3-triazol-1-yl)-6-oxo-3,10,13,16-tetraoxa-7-azaoctadec-1-yl)-12-azido-4,7,10-pentaoxadodecanamide (**S27**).

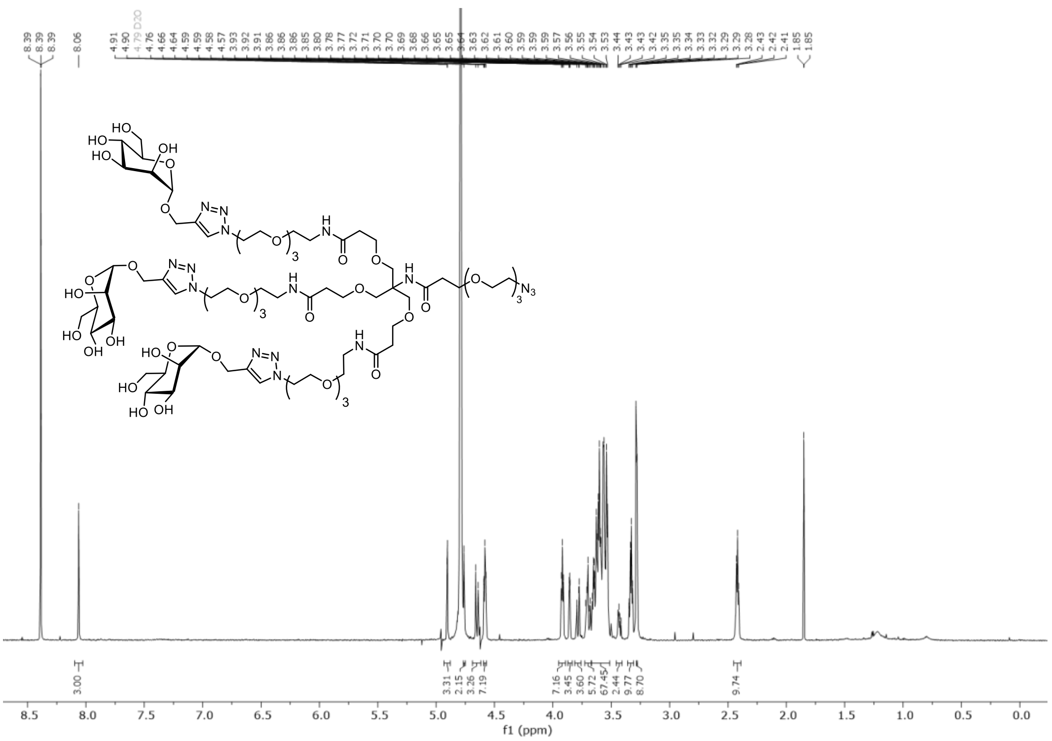

^{1}H-NMR (600 MHz, D_2O): *N*-(1-bis(17-((4-(α-D-mannopyranosyl)-methyl)-1*H*-1,2,3-triazol-1-yl)-5-oxo-2,9,12,15-tetraoxa-6-azaheptadec-1-yl)-18-((4-(α-D-mannopyranosyl)-methyl)-1*H*-1,2,3-triazol-1-yl)-6-oxo-3,10,13,16-tetraoxa-7-azaoctadec-1-yl)-12-azido-4,7,10-pentaoxadodecanamide (**4**).

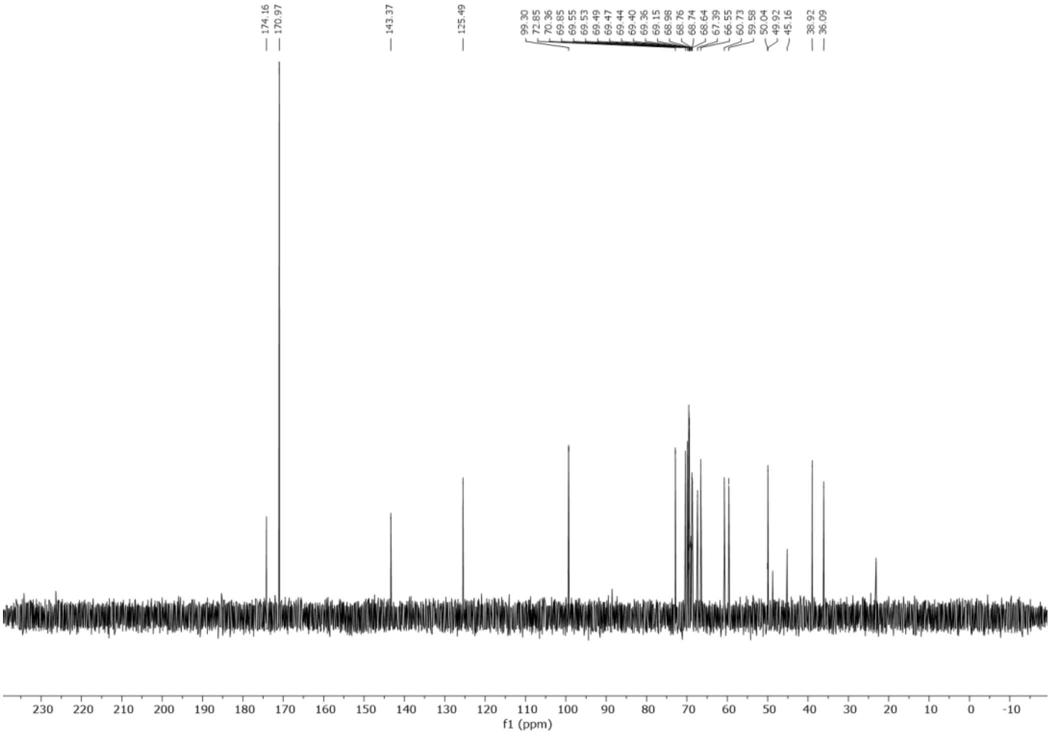

^{13}C-NMR (151 MHz, D_2O): *N*-(1-bis(17-((4-(α-D-mannopyranosyl)-methyl)-1*H*-1,2,3-triazol-1-yl)-5-oxo-2,9,12,15-tetraoxa-6-azaheptadec-1-yl)-18-((4-(α-D-mannopyranosyl)-methyl)-1*H*-1,2,3-triazol-1-yl)-6-oxo-3,10,13,16-tetraoxa-7-azaoctadec-1-yl)-12-azido-4,7,10-pentaoxadodecanamide (**4**).

V. Flow Cytometry Data

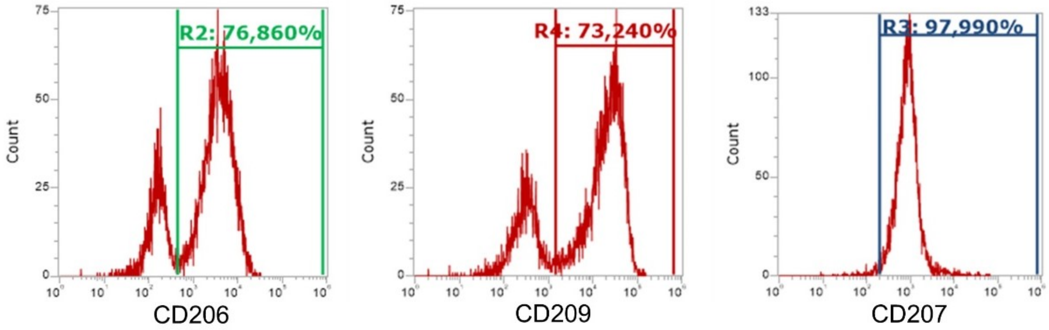

Figure S31: Flow cytometry analysis of the surface expression of the different receptors CD206, CD209 and CD207 on hDCs.

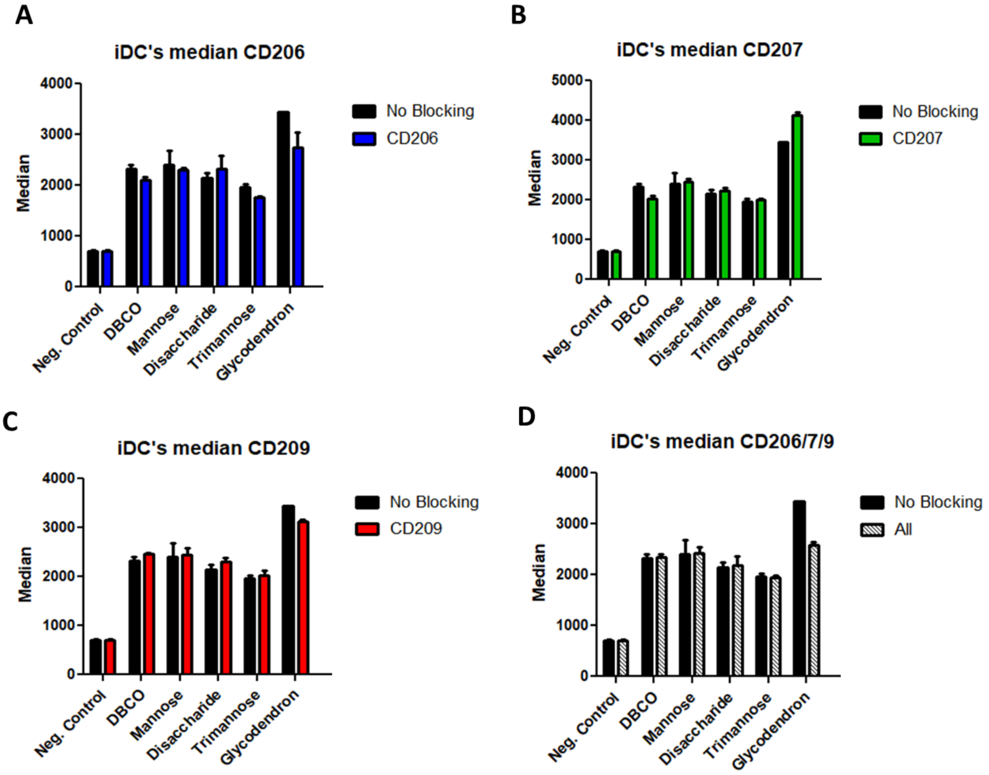

Figure S32: Uptake experiments of differently modified HES NC into hDCs with only one third of the originally used amount of glycodendron. The experiments were performed in duplicates using ANOVA for data evaluation with a p-value = 0.0056 (**A**), p-value = 0.0006 (**B**), p-value = 0.0283 (**C**) and p-value = 0.0003 (**D**).

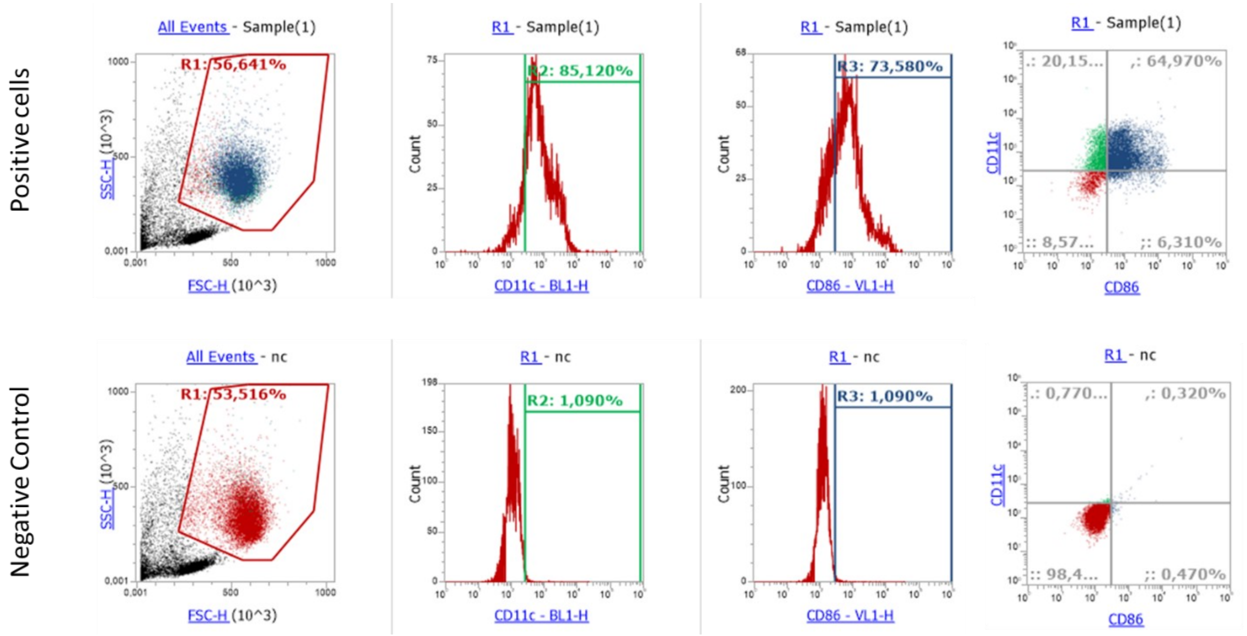

Figure S33: Flow Cytometry plots and histograms demonstrating the abundance of hDC surface markers CD11c and CD86. Positive cells (upper panel) showed 85.12% of CD11c positive cells, while 73.58% of the differentiated cells were positive for CD86.

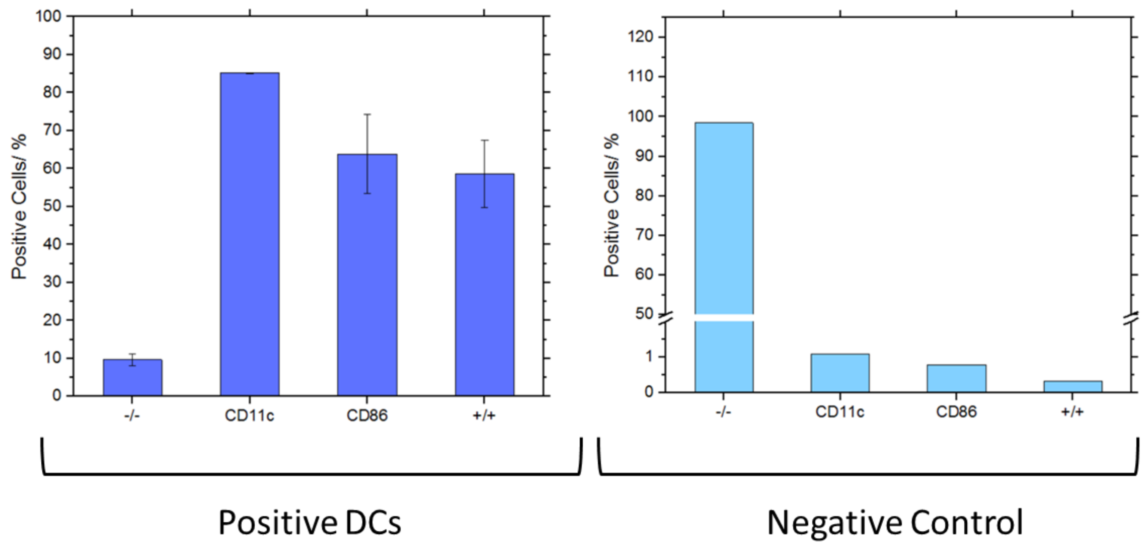

Figure S34: Quantitative amount of surface markers CD11c and CD86 as well as double positive cells (+/+) and double negative (-/-), respectively, present on hDCs after differentiation.

VI. Confocal Laser Scanning Microscopy (cLSM)

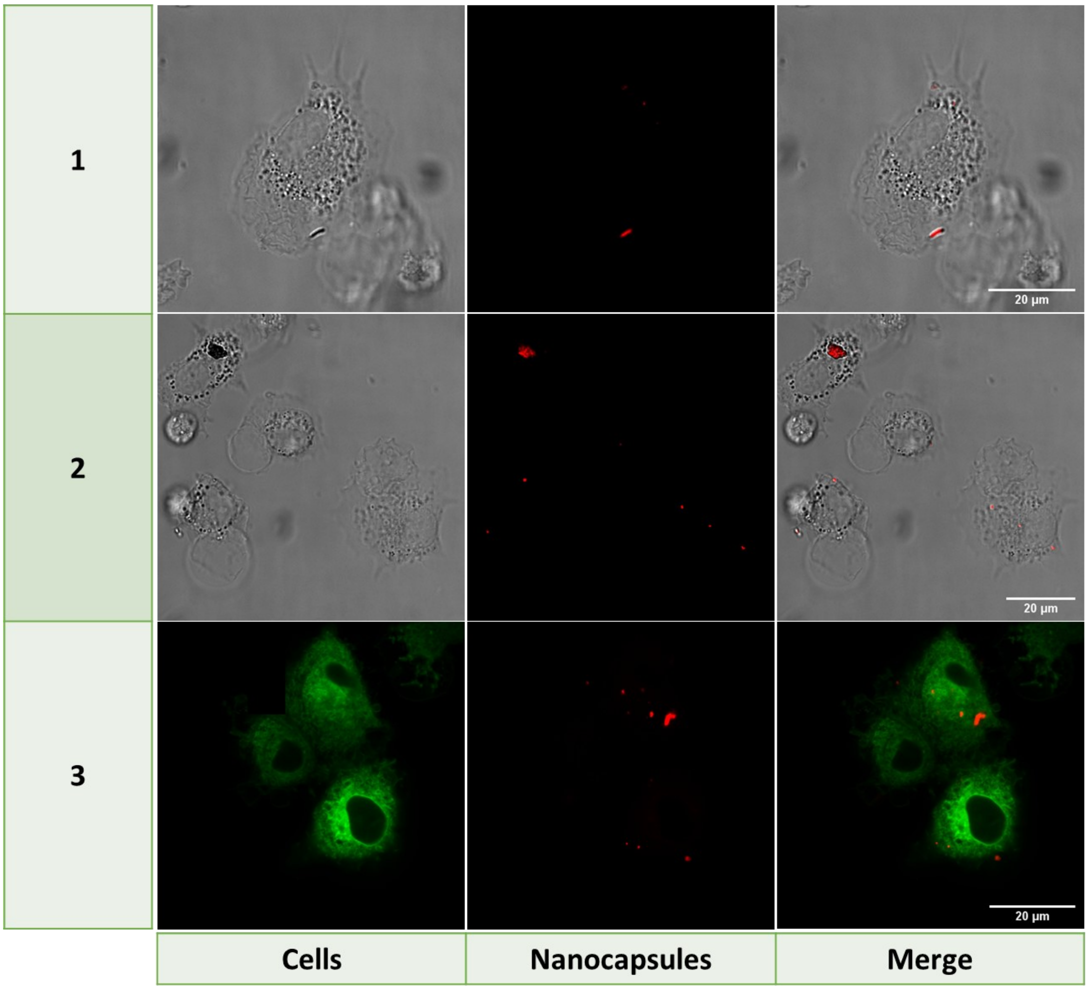

Figure S35: Confocal laser scanning microscopy (cLSM) of hDCs incubated with mannose-decorated HES nanocapsules at a concentration of 75 µg/mL. HCDs, shown in panel 3, were stained with CellMask Green. The cell membrane is pseudo-coloured in green and the nanocapsules are pseudo-coloured in red. Panel 1 and 2 depict the cells using transmission light. The scale bar represents 20 µm.

VII. References

[1] R. Appel, J. Fuchs, S. M. Tyrrell, P. A. Korevaar, M. C. A. Stuart, I. K. Voets, M. Schönhoff, P. Besenius, *Chem. Eur. J.* **2015**, *21*, 19257-19264.

[2] S.-C. Tsai, J. P. Klinman, *Bioorg. Chem.* **2003**, *31*, 172-190.

[3] J. M. Landeros, H. A. Silvestre, P. Guadarrama, *J. Mol. Struct.* **2013**, *1037*, 412-419.

[4] K. M. Bonger, R. J. B. H. N. van den Berg, L. H. Heitman, A. P. Ijzerman, J. Oosterom, C. M. Timmers, H. S. Overkleeft, G. A. van der Marel, *Bioorg. Med. Chem.* **2007**, *15*, 4841-4856.

[5] M. E. Bakleh, V. Sol, K. Estieu-Gionnet, R. Granet, G. Déléris, P. Krausz, *Tetrahedron* **2009**, *65*, 7385-7392.
[6] J. Davila, A. Chassepot, J. Longo, F. Boulmedais, A. Reisch, B. Frisch, F. Meyer, J.-C. Voegel, P. J. Mésini, B. Senger, M.-H. Metz-Boutigue, J. Hemmerlé, P. Lavalle, P. Schaaf, L. Jierry, *J. Am. Chem. Soc.* **2012**, *134*, 83-86.
[7] I. Tavernaro, S. Hartmann, L. Sommer, H. Hausmann, C. Rohner, M. Ruehl, A. Hoffmann-Roeder, S. Schlecht, *Org. Biomol. Chem.* **2015**, *13*, 81-97.
[8] M. Tsakama, Y. Shang, Y. He, B. Fan, F. Wang, W. Chen, X. Dai, *Tetrahedron Lett.* **2016**, *57*, 1739-1742.
[9] M. Glaffig, B. Palitzsch, S. Hartmann, C. Schüll, L. Nuhn, B. Gerlitzki, E. Schmitt, H. Frey, H. Kunz, *Chem. Eur. J.* **2014**, *20*, 4232-4236.
[10] S. Kramer, J. Langhanki, M. Krumb, T. Opatz, M. Bros, R. Zentel, *Macromol. Biosci.* **2019**, *19*, 1800481.
[11] B. Vauzeilles, B. Dausse, S. Palmier, J.-M. Beau, *Tetrahedron Lett.* **2001**, *42*, 7567-7570.
[12] R. Daly, G. Vaz, A. M. Davies, M. O. Senge, E. M. Scanlan, *Chem. Eur. J.* **2012**, *18*, 14671-14679.
[13] M. Serpi, R. Bibbo, S. Rat, H. Roberts, C. Hughes, B. Caterson, M. a. J. Alcaraz, A. T. Gibert, C. R. A. Verson, C. McGuigan, *J. Med. Chem.* **2012**, *55*, 4629-4639.
[14] H. Vankayalapati, G. Singh, *J. Chem. Soc., Perkin Trans. 1* **2000**, 2187-2193.
[15] M. Dowlut, D. G. Hall, O. Hindsgaul, *J. Org. Chem.* **2005**, *70*, 9809-9813.
[16] Y. Shota, N. Seiichi, Y. Kazuo, *Chem. Lett.* **2013**, *42*, 791-793.
[17] S. S. Iyer, S. M. Rele, S. Baskaran, E. L. Chaikof, *Tetrahedron* **2003**, *59*, 631-638.
[18] G. Zemplén, A. Gerecs, I. Hadácsy, *Ber. Dtsch. Chem. Ges.* **1936**, *69*, 1827-1829.
[19] S. Yamamoto, S. Nakahama, K. Yamaguchi, *Chem. Lett.* **2013**, *42*, 791-793.
[20] B. Amit, E. Hazum, M. Fridkin, A. Patchornik, *Int. J. Pept. Protein Res.* **1977**, *9*, 91-96.
[21] V. Percec, P. Leowanawat, H.-J. Sun, O. Kulikov, C. D. Nusbaum, T. M. Tran, A. Bertin, D. A. Wilson, M. Peterca, S. Zhang, N. P. Kamat, K. Vargo, D. Moock, E. D. Johnston, D. A. Hammer, D. J. Pochan, Y. Chen, Y. M. Chabre, T. C. Shiao, M. Bergeron-Brlek, S. André, R. Roy, H.-J. Gabius, P. A. Heiney, *J. Am. Chem. Soc.* **2013**, *135*, 9055-9077.
[22] G. Baier, A. Musyanovych, M. Dass, S. Theisinger, K. Landfester, *Biomacromolecules* **2010**, *11*, 960-968.
[23] G. Baier, J. M. Siebert, K. Landfester, A. Musyanovych, *Macromolecules* **2012**, *45*, 3419-3427.
